# Phylogenetic analysis of the genus *Laparocerus*, with comments on colonisation and diversification in Macaronesia (Coleoptera, Curculionidae, Entiminae)

**DOI:** 10.3897/zookeys.651.10097

**Published:** 2017-02-02

**Authors:** Antonio Machado, Eduardo Rodríguez-Expósito, Mercedes López, Mariano Hernández

**Affiliations:** 1 Chopin 1, 38208 La Laguna, Tenerife, Canary Islands, Spain; 2 Instituto Universitario de Enfermedades Tropicales y Salud Pública de Canarias; 3 Departamento de Bioquímica, Microbiología, Biología Celular y Genética. Universidad de La Laguna. Avda. Astrofisico Fco. Sánchez s/n 38207 La Laguna, Tenerife, Spain

**Keywords:** Back-colonisation, Bayesian inference, Canary Islands, dispersal, divergence rates, introgression, island evolution, Madeira, mitochondrial DNA, Moreiba Morocco, new subgenera, phylogeny, Selvagens Islands, speciation, weevils

## Abstract

The flightless Entiminae weevil genus *Laparocerus* is the species-richest genus, with 237 species and subspecies, inhabiting Macaronesia (Madeira archipelago, Selvagens, Canary Islands) and the continental ‘Macaronesian enclave’ in Morocco (one single polytypic species). This is the second contribution to gain insight of the genus and assist in its systematic revision with a mitochondrial phylogenetic analysis. It centres on the Canarian clade, adding the 12S rRNA gene to the combined set of COII and 16S rRNA used in our first contribution on the Madeiran clade (here re-analysed). The nuclear 28S rRNA was also used to produce an additional 4-gene tree to check coherency with the 3-gene tree.

A total of 225 taxa (95%) has been sequenced, mostly one individual per taxa. Plausible explanations for incoherent data (mitochondrial introgressions, admixture, incomplete lineage sorting, etc.) are discussed for each of the monophyletic subclades that are coincident with established subgenera, or are restructured or newly described. The overall mean genetic divergence (p-distance) among species is 8.2%; the mean divergence within groups (subgenera) ranks from 2.9 to 7.0% (average 4.6%), and between groups, from 5.4% to 12.0% (average 9.2%). A trustful radiation event within a young island (1.72 Ma) was used to calibrate and produce a chronogram using the software RelTime.

These results confirm the monophyly of both the Madeiran (36 species and subspecies) and the Canarian (196 species and subspecies) clades, which originated ca. 11.2 Ma ago, and started to radiate in their respective archipelagos ca. 8.5 and 7.7 Ma ago. The Madeiran clade seems to have begun in Porto Santo, and from there it jumped to the Desertas and to Madeira, with additional radiations. The Canarian clade shows a sequential star-shape radiation process generating subclades with a clear shift from East to West in coherence with the decreasing age of the islands. *Laparocerus garretai* from the Selvagens belongs to a Canarian subclade, and *Laparocerus susicus* from Morocco does not represent the ancestral continental lineage, but a back-colonisation from the Canaries to Africa. Dispersal processes, colonisation patterns, and ecological remarks are amply discussed. Diversification has been adaptive as well as non-adaptive, and the role of ’geological turbulence’ is highlighted as one of the principal drivers of intra-island allopatric speciation.

Based on the phylogenetic results, morphological features and distribution, five new monophyletic subgenera are described: *Aridotrox* **subg. n.**, *Belicarius* **subg. n.**, *Bencomius* **subg. n.**, *Canariotrox* **subg. n.**, and *Purpuranius* **subg. n.**, totalling twenty subgenera in *Laparocerus*.

## Introduction

*Laparocerus* Schönherr, 1834 are flightless Entimine weevils with free-living edaphic larvae, and most are oligophagous and climb vegetation to feed upon the leaves (Machado 2003). The adults of some species live in the leaf-litter, and there are even some which are edaphic or are adapted to dwell in the volcanic underground environment. All species are endemic to the oceanic islands of Macaronesia (Madeira, Selvagens, and Canary Islands), with the exception of one polytypic species, endemic to west Morocco, on the mainland. They are not known from the Cape Verde Islands, while the species from the Azores originally attributed to *Laparocerus* as subgenus *Drouetius* Méquignon, 1942 represent a separate Azorean endemic and rather distant genus (Machado 2009a).

**Figure 1.**
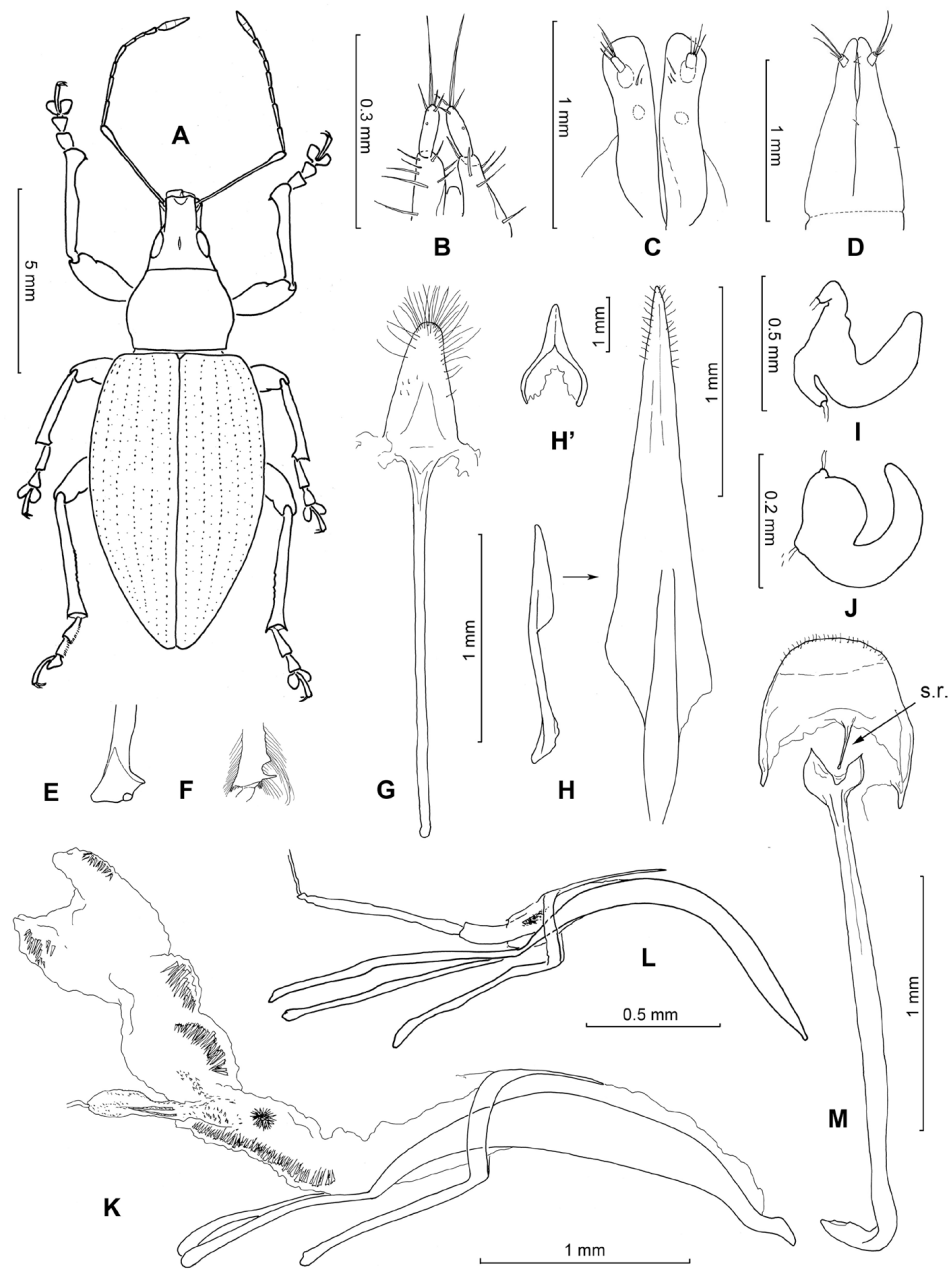
Morphological details of *Laparocerus* Schoenherr, 1834. **A** Imago of Laparocerus (Bencomius) undatus Wollaston,1864 **B** Gonostyli of Laparocerus (Purpuranius) longipennis Machado, 2011 **C** Gonostyli of Laparocerus (Machadotrox) excavatus Wollaston, 1864 **D** Gonostyli of Laparocerus (Bencomius) undatus Wollaston, 1864 **E** Male metatibia of Laparocerus (Atlantis) noctivagans Wollaston, 1854 **F** Male metatibia of Laparocerus (Aridotrox) rasus rasus Wollaston, 1864 **G** Female sternite VIII of Laparocerus (Pecoudius) grayanus Wollaston, 1864 **H** Female sternite VIII and H’ terguite VIII of *L* (*Canariotrox*) *estevezi* Machado, 2012 **I** Spermatheca of Laparocerus (Guanchotrox) tafadensis Machado, 2016 **J** Spermatheca of Laparocerus (Laparocerus) morio Boheman, 1834 **K** Aedeagus of Laparocerus (Belicarius) longiclava Lindberg, 1953 **L** Aedeagus of Laparocerus (Pseudatlantis) abditus (Woll. 1864) **M** Male sternites IX and VII of Laparocerus (Fernandezius) impressicollis Wollaston, 1864 (s.r = spiculum relictum).

The external morphological disparity within this Entimine lineage is extraordinary and explains why several species groups were originally attributed to other genera (e.g. *Omias* Germar, 1826) or established as separate genera: *Atlantis* Wollaston, 1854, *Cyphoscelis* Wollaston, 1864, *Lichenophagus* Wollaston, 1854 or *Anillobius* Fauvel, 1907. At present all of them are lumped in *Laparocerus* (Machado 2013). However, the morphological characterisation of such a wide concept of *Laparocerus* is not easy and still poses a challenge. In addition to its restricted distribution, there are only a few shared features that characterise the species of this group: (a) the presence of a spiculum relictum in the post-tegminal membrane representing the VIII male sternite (Fig. 1M), (b) the insertion of the seminal duct at a secondary poach (gonoporal diverticulum) of the internal sac of the aedeagus, which detaches either laterally or from the tip of the internal sac (Fig. 1K–L), (c) the metanepisternum narrow and basally protruding over the outer angle of the metacoxa hiding its contact with the elytral margin, and (d) the elytral declivity not overhanging the abdominal apex.

**Figure 2.**
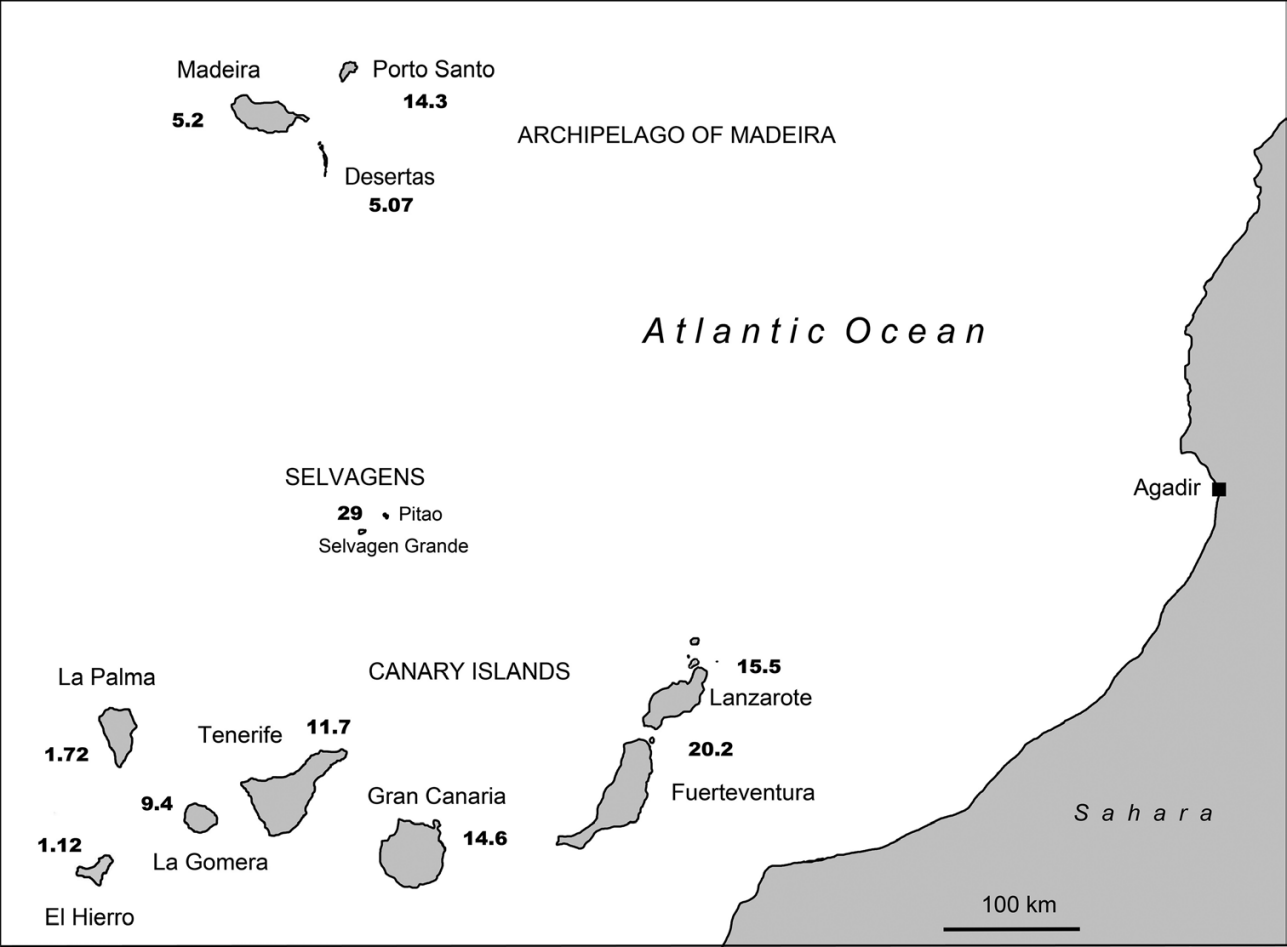
Geological ages of the Canary Islands and Selvagens (Carracedo 2011), Madeira island (Schmincke 1998), Porto Santo (Geldmacher et al. 2000), and the Desertas (Schwartz et al. 2005) in million of years (Ma).

Nearly one decade ago, Machado et al. (2008a) published an analysis of the Madeiran clade based on mitochondrial DNA to assist in the taxonomic revision of the genus. Since that publication, the first author has described several new species, mainly from the Canary Islands (Machado 2007b, 2008a, 2008b, 2009b, Machado and García 2010, Machado 2011a, 2011b, 2012a, 2013, 2015, 2016), increasing the number of species level taxa from 117 to 237. These descriptions were necessary before addressing the molecular analysis of the Canarian clade, which is the main purpose of the present work. As before, the molecular analysis centred on mitochondrial markers aims at inferring a species phylogeny to gain insight of the group, and to better support the systematic decisions, principally at the genus and subgenus levels.

For the molecular analysis of the Madeiran clade, only one specimen per taxon and two mitochondrial genes were chosen: fragments of cytochrome oxidase subunit II and of ribosomal 16S RNA subunit. These are frequent markers used in many phylogenetic studies (Gómez Zurita and Galián 2005; Sequeira et al. 2008). In addition, a fragment of the nuclear elongation factor 1-alpha gene was analysed for a representative taxon of each morphological group in order to elucidate deeper nodes or for checking controversial results.

The number of Canarian OTUs (219) is much higher than in the Madeira study (35) and a larger character set was needed to increase phylogenetic information. Sequences of mitochondrial 12S rRNA gene were added to expand the signal, and to check for consistency we opted for the nuclear 28S rRNA gene (regions D2-D3), covering all OTUs.

We conceive the genus as a phylogenetic unit with biogeographical consistency in prevalence to its morphological distinctiveness. Therefore, it is also a purpose of this contribution to clarify if the extant abundant *Laparocerus* evolved by radiation within Macaronesia after a single colonisation event in Madeira and in the Canary Islands; or whether we are facing the result of several phyletic lines of Laparocerini that arrived to the islands and went extinct in the continent thereafter. The genus *Laparocerus* could either be organised in several subgenera, or split in many genera, depending on which of the respective hypothesis is better supported by the molecular analysis and concurrent information. Consequently, we address here a time analysis of the whole group.

There are many limitations imposed on our study by analysing mostly only one individual from each species (*vide* Funk and Omland 2003), but this should have a low impact on the deeper nodes of the phylogram and on our main objectives.

The present phylogenetic analysis includes both the Canarian and the Madeiran clades, but the latter whose analysis has been previously addressed (Machado et al. (2008), will only be partially discussed on this occasion. In order to facilitate the presentation of our results and the overall discussion, we use subgeneric names for the monophyletic subclades, and some new subgenera are here described in a separate section. Moreover, some information about the species distribution and ecology that will be presented in a future monograph has been slightly introduced here to enrich the comments and place results in the general context.

## Materials and methods

### Sampling

Approximately 46,500 specimens of *Laparocerus* were collected in the field and identified by the first author, unless otherwise specified in Appendix 1. At least two specimens of each presumably distinct species or subspecies were directly introduced in absolute ethanol, and in many cases this was repeated for different localities. Ethanol was replaced the next day and samples preserved at -20°C. Voucher card-mounted dry specimens and specimens preserved in ethanol, from the same locality and date, share the same database collection number. This information for the specimens included in the present study is provided in Appendix 1. All this material is kept in the Machado Collection, La Laguna, Tenerife.

Eleven known species were not found alive in nature and for this reason they were excluded from the present analysis. From Madeira: Laparocerus (Lichenophagus) acuminatus (Wollaston, 1854), Laparocerus (Atlantodes) navicularis (Wollaston, 1854), Laparocerus (Atlantodes) lanatus (Wollaston, 1854), and Laparocerus (Anillobius) porctosantoi (Franz, 1970); from the Selvagens *Laparocerus garretai albosquamosus* Machado, 2011; and from the Canaries: Laparocerus (Purpuranius) fraterculus Machado, 2012 and several hypogean species or subspecies of the subgenus *Machadotrox*, which are normally scarce and difficult to obtain: *Laparocerus zarazagai zarazagai* García and Oromí, 1997; *Laparocerus iruene* García and Machado, 2011; *Laparocerus machadoi* García and González, 2006; *Laparocerus idafe* García and Alonso Zarazaga, 2011, and *Laparocerus cavernarius* Machado, 2011. This set of missing taxa in the analysis represents nearly about 5% of the total of known *Laparocerus* (237).

Plant genera mentioned in the text, and their respective families (Mabberley 1997) are listed in Appendix 3.

### DNA isolation, amplification and sequencing

In our first contribution (Machado et al. 2008) fragments of the mitochondrial cytochrome c oxidase subunit II (COII, 598 bp), the 16S ribosome large subunit RNA (16S rRNA, 427 bp), and, only for representatives of species groups, the nuclear elongation factor 1 alpha (EF-1α, 611 bp) were used as genetic markers. In addition to the aforementioned molecular markers, in this study we also use the mitochondrial 12S ribosomal RNA (12S rRNA, 344 bp) and the nuclear 28S ribosomal RNA (28S rRNA, 762 bp) genes both sequenced for all Madeiran and Canarian OTUs (see Appendix 1).

DNA was extracted using DNeasy Blood & Tissue Kit (Qiagen, Valencia, CA Inc) following the instructions of the manufacturer. All PCR reactions were carried out in a Veriti Thermal Cycler (Applied Biosystems, USA) in a final volume of 25 µl containing 1× buffer (GeneAll, Korea), 150 µM of each dNTP, 0.2 µM of each primer, 0.5 U AmpONE™ Taq DNA polymerase (GeneAll, Korea) and 10–20 ng of DNA template. Thermal profile for COII, 16S rRNA and EF-1α fragments were as described in Machado et al. (2008). PCR conditions for 12S rRNA and 28S rRNA were as follows: 2 min at 94°C followed by 35 cycles of denaturation at 94°C for 10 s, annealing at 54 or 56°C respectively for 20 s, and extension at 72°C for 30 s, with a final extra extension step at 72°C for 5 min. Subsequently, the primers and nucleotides were removed with Illustra^TM^ ExoProStar^TM^ 1-Step (GE Heathcare, Life Sciences) according to the manufacturer’s instructions. Primers used for the PCR are listed in Table 1. Sequencing was carried out by the sequencing facilities of the company MacroGen Europe (Amsterdam).

**Table 1.** Sequence of primers used.

COII	TL-J-3037 (TED)	5'-TAATATGGCAGATTAGTGCATTGGA-3'	Simon et al. 1994
	TK-N-3785 (EVA)	5'-GAGACCATTACTTGCTTTCAGTCATCT-3'	Gómez-Zurita et al. 2000
16S rRNA	16SBr’	5'-CCGGTCTGAACTCAGATCATGT-3'	Machado et. al. 2008
	16SM	5'-CCAATGAAGTTTTAAATGGCCGC-3'	Simon et al. 1994
12S rRNA	SR-J-14233 (f)	5'- AAGAGCGACGGGCGATGTGT-3'	Kergoat et al. 2004
	SR-N-14588	5'- AAACTAGGATTAGATACCCTATTA T-3'	Kergoat et al. 2004
28S rRNA	S3690	5'-GAGAGTTMAASAGTACGTGAAAC-3'	Sequeira et al. 2000
	A4394	5'-TCGGARGGAACCAGCTACTA-3'	Sequeira et al. 2000
EF-1α	EFA754	5'-CCACCAATTTTGTAGARATC-3'	Normark et al. 1999
	EFS149T	5'-AAGGAGGCTCARGAAATGGG-3'	Idem, modified

One specimen, preferential from the type locality, was analysed for each of the 225 taxa sampled, except when the taxon was present in different islands, in known putative vicariance regions within the same island (e.g. Teno/Anaga in Tenerife), or when morphological differences associated with marginal localities were noticed. Due to such situations, a total of 30 additional sequences was included in the analysis. Moreover, in order to minimise laboratory errors (contamination, mislabelling, etc.) sequencing was repeated for discordant results, particularly, with taxa strangely placed according to traditional morphology (occasionally a second specimen from the same locality was used). Sequencing with both the forward and reverse primers was performed only in cases of not clean or incomplete chromatograms. For a few species (*Laparocerus aethiops*, *Laparocerus auarita*, *Laparocerus canariensis*, *Laparocerus morio*, *Laparocerus vespertinus*), several individuals from the same locality were sequenced for COII to get a more accurate idea of the range of local intraspecific genetic divergence with this marker.

A total of 1425 sequences was obtained: 441 for the COII, 322 for the 16S rRNA, 294 for the 12S rRNA, 290 for the 28S rRNA, and 78 for the EF-1α. All duplicate and redundant sequences – from the same or different localities – were removed from the combined matrix of COII+16S rRNA+12S rRNA for the final analysis, which ended up with a total of only 256 OTUs, representing 223 different *Laparocerus* taxa and two outgroups. This final set of sequences has been deposited in GenBank (www.ncbi.nlm.nih.gov/Genbank) with following accession numbers: EF583315 – EF583371, FJ495251 – FJ495253, KX551687 – KX551907 for the COII; KX550955 – KX551210 for the 12S rRNA; FJ495254 – FJ495256, KX551211 – KX551431 for the 16S rRNA; KX551432 – KX551686 for the 28S rRNA; and EF583372 – EF583389, KX551908 – KX551958 for the EF-1α.

### Outgroup selection

The relationships among the many genera and tribes of Entiminae are still unsolved and pose long endeavour ahead (*vide* Oberprieler et al. 2007, Hundsdoerfer et al. 2009). Very few phylogenetic studies attempting to organise the present chaos include any *Laparocerus* or are little conclusive (e.g. Davis 2014, Stüben et al. 2015).

Alonso-Zarazaga and Lyal (1999) listed eight genera for the tribe Laparocerini Lacordaire, 1863, of which six have later been removed and assigned to other tribes (Machado 2010, Alonso-Zarazaga 2013). The only genus left besides *Laparocerus*, was *Straticus* Pascoe, 1886 which presumably belongs also to the African Peritelini as do some of the other putative Laparocerini. *Moreiba* Alonso-Zarazaga, 2013 recently described from the Canary Islands, is a true Laparocerini with presence of *spiculum relictum* in the male sternite VIII, but the internal sac of aedeagus has the gonoporus in normal position and the tibiae are lacking mucro. It was tested as an outgroup and will be commented upon below.

In the Madeiran clade analysis (Machado et al. 2008) *Rhyncogonus excavatus* Van Dyke, 1937 from the island of Rurutu in the French Polynesia, was selected and used as a formal outgroup. In Machado (2010) several other genera of Entiminae were checked for closer relationship with *Laparocerus* based on 16S rRNA sequences obtained by us or taken from the NCBI (National Centre of Biotechnology Information). Unfortunately, the number of different genera sequenced is limited and *Laparocerus* does not relate with confidence (Bayesian support) to any other genus. Nonetheless, we selected new outgroups in order to avoid long-branch attraction (s. Anderson and Swofford 2004), those being *Barypeithes indigens* (Boheman, 1834) (Sciaphilini), and *Brachyderes rugatus* Wollaston, 1864 (Brachyderini), two of the less divergent (p-distance) genera tested.

*Moreiba* was also tested directly as outgroup, but it showed unstable behaviour jumping from the Madeiran clade to the Canarian clade or outside both of them, depending on the individual gene or combination of genes used. *Moreiba* is clearly related to *Laparocerus* from the morphological point of view (Alonso-Zarazaga 2013), but lacking DNA from other genera of Laparocerini that could help fixing its position, we disregarded it as an outgroup and excluded it from the final analysis.

### Data analysis

DNA sequences were viewed, edited and assembled using MEGA 6 (Tamura et al. 2013). Alignments were achieved using the program Muscle (Edgar 2004) with default parameters as implemented in MEGA 6 and tuned by eye. Each marker was tested for hipervariational loci with GBlocks (Castresana 2000); no fragments were removed. The plausibility of the alignment of COII and EF-1α sequences was verified at the amino acid level. The entropy-based index as implemented in Dambe 5.2.78 (Xia et al. 2003) was used to assess substitution saturation within the mtDNA and 28S rRNA sequences, with negative results.

Alignment of 16S rRNA and 28S rRNA included 5 and 17 indels, respectively. These positions were considered as missing data for all analyses. In the case of the 12S rRNA, shared indels seemed to express relations judging from the known taxonomy (e.g. same subgenus) and were coded (1 or 0) with FastGap 1.2 (Borchsenius 2009), increasing the sequences from 344 bp to 364 bp.

Genetic divergence of all sequence pairs (genetic distance, gamma distributed with invariant sites G+I), the p-distance means between and within each subgenus, and the means between and within the Canarian and Madeiran subsets were calculated with MEGA7 (Kumar et al. 2016) after removal of all positions containing gaps and missing data, and eliminating duplicate taxa by keeping only the sequence of the type-locality specimen or that with the higher divergence value (223 taxa in the final dataset). In the calculation of within subgenera divergence, species grouped as *incertae sedis* and *Lichenophagus* (only one species available) were not considered.

Phylogenetic relationships were reconstructed using Bayesian inference (BI). Nucleotide substitution model parameters were obtained with jModelTest 2.1.4 (Darriba et al. 2012) using the Bayesian Information Criterion (Schwarz 1978), with the following results: TIM1+I+G for the mtCOII; TIM3+I+G for the 16S rRNA; HKY+I+G for the 12S rRNA, and for the nuclear markers TVM+I+G for the 28S rRNA, and TIM2+IU+G for the EF-1α.

Analyses were conducted using Bayesian Markov chain Monte Carlo inference (Yang and Rannala 1997) as implemented with MrBayes 3.2.3 (Ronquist et al. 2012) running on the facility Mobyle SNAP Workbench at the North Carolina State University (Monacell and Carbone 2014). We used the combined dataset of the mitochondrion markers partitioned (256 OTUs, 1389 bp), and the previously determined models of nucleotide evolution. Parameters were treated as unknown variables with equal *a priori* probability and subsequently estimated by the programme during the analysis. Starting trees were randomly chosen. Two independent 10,000,000 generation runs of eight Monte Carlo Markov chains ‒ two cold, six heated at 0.02 ‒, were conducted (nswap = 5), and trees being sampled every 100 generations for a total of 100,000 trees in each of the initial samples. Variations in likelihood scores were examined graphically with the Tracer v1.6 application (Rambaut et al. 2014) and the first 2,500,000 generations were discarded, having ensured that stationarity was reached. Accordingly, the first 25,000 trees were discarded as burn-in, and the following 75,000 trees were used to estimate topology and tree parameters.

Similar BI analysis were repeated, 16,000,000 generations, adding to the mitochondrial matrix the 28S rRNA sequences (total 2.151 bp) and, for some selected OTUs (78), the EF-1α (total 2.762 bp). The trees obtained were used to check consistency with the mitochondrial only based results. We confirmed that there is no significant incongruence between the information provided by each gene using the partition homogeneity test of Farris et al. (1994) as implemented in PAUP*4.0b10 (Swofford 2002), with 500 replicates. The nuclear genes were the most incongruent (with p = 0.10, but lower than p = 0.05) and the values obtained for the three mitochondrial genes (p = 0.894) and for the whole set of five mitochondrial and nuclear genes (p = 0.868) reflect total congruency.

Maximum likelihood trees for all markers and combined sets were also reconstructed using RAxML (Stamatakis 2014) as implemented in Mobyle SNAP Workbench under the above-mentioned models. No differences with the BI trees were found.

The BI final phylogram was edited with TreeGraph 2 (Stöver and Müller 2010) and, due to its length, the tree was fully collapsed or divided in separate pieces for presentation. Clades and subclades are organised from older (bottom of tree) to younger (top of tree). Species names are coloured in the phylograms to indicate the island of origin of the specimen sequenced (a legend is provided with each subtree). In most cases, *Laparocerus* species are single-island endemics and that gives an overall idea of their distribution. Species that live in more than one island have been sequenced for each island, with the exception of *Laparocerus ellipticus* from El Hierro, which failed.

The colonisation pathways have been inferred from the tree topology under criterion of parsimony, assuming the uncertainty derived from having analysed mostly one specimen per species, and lacking total knowledge about extinctions.

### Dating

A molecular clock test was performed with MEGA7 by comparing the ML value for the given topology with and without the molecular clock constraints under GTR model. The null hypothesis of equal evolutionary rate throughout the tree was rejected at a 5% significance level (P = 0) for all individual markers. Consequently, a timetree was built using the program RelTime (Tamura et al. 2012) under relaxed molecular clock hypothesis (local clocks) and using the combined mitochondrial tree obtained from the BI analysis. This module of MEGA7 calculates divergence times using the Maximum Likelihood method based on the General Time Reversible model (Nei and Kumar 2000). The relative times are converted to absolute divergence times based on constraints of the calibration point supplied.

Twenty potential calibration points were tested giving preference to the nodes of vicariant species present in El Hierro or La Palma, or radiations within these islands which are the youngest in the Canaries, with a geological age of 1.12 Ma (Carracedo 2011) and 1.72 Ma respectively (Guillou et al. 2001). Species with a plausible ancestor from a source-island that was not sequenced or is unknown were disregarded. For instance, Laparocerus (Amyntas) incomptus is endemic to El Hierro and shows a basal position as sister taxon to the rest of species in this subgenus that inhabit much older islands. However, there is no *Amyntas* known from La Gomera (9.4 Ma) from where it probably originated. In the case of Madeira, nodes implying radiations within the main island (4.8–5.2 Ma, f. Schmincke 1998) were chosen. Following the idea of Near and Sanderson (2004), but without fossils, we crosschecked the calibration points, disregarding those chronograms when any of the points surpassed the age of their island.

Finally, a single calibration point (see black triangle in Fig. 7) was selected, namely the radiation event of four species of the subgenus *Machadotrox* which are endemic to La Palma (two of them hypogean and blind), not allowing it to be older than the island age of 1.72 Ma, or younger than 0.21 Ma. This minimum age constrain was obtained by starting with 0.01 Ma and increasing it until the estimated age of Node P of Madeira (*Atlantis* and *Pseudatlantis* species) dropped below the age attributed to this island. That gives an ample margin for colonisation of La Palma to happen after the island emerged.

In the chronogram obtained (see Suppl. materials 1–3) bars around each node represent 95% confidence intervals which were computed using the method described in Tamura et al. (2013). The estimated log likelihood value of the topology shown is -32807.3327. A discrete Gamma distribution was used to model evolutionary rate differences among sites (6 categories (+G, parameter = 0.5622)). The rate variation model allowed for some sites to be evolutionarily invariable ([+I], 42.7318% sites).

In the summarized Table 2 mean values of age estimates are included for the main nodes and MRCA (most recent common ancestor) of subclades representing subgenera, as they should be more reliable than divergence times at leaf levels subject to individual variability. Having worked mostly with one specimen per species implies risk of depicting relations linked to the particular individual sequenced, and that may inflate or deflate inferred times and rates of evolutionary divergence if there is some kind of underlying polyphyly in the species (Funk and Omland 2003). Our date estimates must to be taken with some caution.

**Figure 3.**
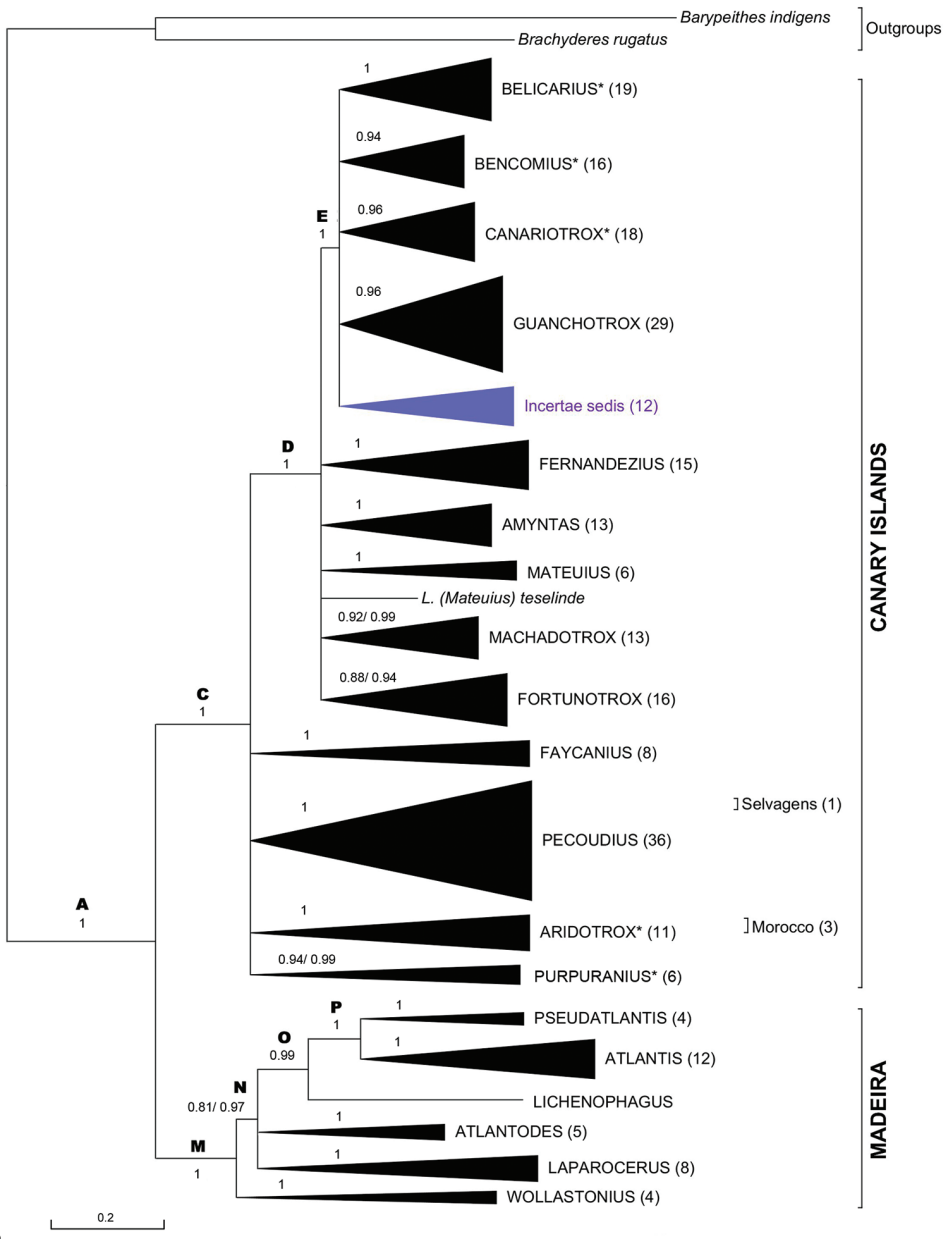
Bayesian 50% majority rule consensus tree for COII, 12S rRNA, and 16S rRNA of genus *Laparocerus* Schönherr, 1834. Nodes showing Bayesian posterior probabilities (after slash, when adding 28S rRNA to dataset). Subclades collapsed and named after subgenera, with number of OTUs in brackets. Total OTUs = 256. Genetic divergence in scale bar.

**Table 2.** Ages in million years of *Laparocerus* subgenera calculated with RelTime. Basal nodes codes after the phylogram in Fig. 3.

Clades with ages						TMRCA	Distribution											
Node **A** 11.2 Ma	Node **C** Canarian Clade 7.7 Ma	Node **D** 5.3 Ma	Node **E** 4.6 Ma	***Belicarius*** subg. n.		2.8 Ma					H	P	G	T	C			
				***Bencomius*** subg. n.		3.1 Ma						P	G	T				
				***Canariotrox*** subg. n.		3.3 Ma					H	P		T	C			
				***Guanchotrox*** Alonso-Zarazaga & Lyal, 1999		4.2 Ma					H	P	G	T	C			
				*Incertae sedis*		–					H	P	G	T	C			
			***Fernandezius*** Roudier, 1957			2.2 Ma					H	P		T				
			***Amyntas*** Wollaston, 1865			2.5 Ma					H	P		T	C			
			***Mateuius*** Roudier, 1957			3.3 Ma					H		G					
			***Machadotrox*** Alonso-Zarazaga & Lyal, 1999			4.7 Ma					H	P	G	T				
			***Fortunotrox*** Machado, 2011			5.1 Ma					H	P	G	T				
		***Faycanius*** Machado, 2012				1.9 Ma									C			
		***Pecoudius*** Roudier, 1957 s.l.				4.2 Ma				S	H	P	G	T	C			
		***Aridotrox*** subg. n.				4.8 Ma										F	L	A
		***Purpuranius*** subg. n.				5.9 Ma								T		F	L	
	Node **M** Madeiran Clade 8.5 Ma	Node **N** 7.2 Ma	Node **O** 6.3 Ma	Node **P** 5.1 Ma	***Pseudatlantis*** Machado, 2008	2.6 Ma	M		Ps									
					***Atlantis*** Wollaston, 1854	5.1 Ma	M											
				[Anillobius* Fauvel, 1907]		?	M		Ps									
				***Lichenophagus*** Wollaston, 1854		?		D	Ps									
			***Atlantodes*** Machado, 2008			5.5 Ma	M		Ps									
			***Laparocerus*** Schoenherr, 1834			5.8 Ma	M	D	Ps									
		***Wollastonius*** Machado, 2008				3.3 Ma	M	D										

* not included in the phylogram

Distribution codes: **M** Madeira, **D** Desertas, **Ps** Porto Santo, **S** Selvagens, **H** El Hierro, **P** La Palma, **G** La Gomera, **T** Tenerife, **C** Gran Canaria, **F** Fuerteventura, **L** Lanzarote, **A** Africa (Morocco).

## Results with comments

### DNA sequence variation

For the COII, 326 out of 598 positions (54.5) were variable and 272 informative (45.5%); for the 12S rRNA raw fragment 184 out of 344 positions (53.5%) were variable and 145 informative (42.28%), and for the 16S rRNA fragment, 163 out of 427 positions (38.2%) were variable and 122 informative (28.6%). Third positions of COII showed low G composition (1.7%) as is typical in insect mitochondrial DNA coding genes.

Xia’s index for substitution saturation in COII produced values of 0.074 (first and second codon positions) and 0.46 (third codon position) which were significantly lower than the critical value for symmetric topologies (0.69–0.78, P < 0.001; 0.69–0.77, P < 0.001 respectively), suggesting that sites have reached little saturation and sequences can be reliably used for phylogenetic reconstruction. In the case of the 28S rRNA and the other genes saturation was also amply disregarded (data not shown).

The overall mean genetic divergence (p-distance) among species is 11.7% in COII, 5.4% in 12S rRNA, 5.2% in 16S rRNA, 1,0% in 28S rRNA, and 8.2% in the combined set. To gain a rough idea of the local genetic divergence, we sequenced the COII of five species with four specimens each collected in the same locality, obtaining the highest value of 1.3% in the case of *Laparocerus canariensis* from El Portillo, Tenerife.

The overall mean p-distance in the mitochondrial 3-gene combined matrix is 8.5% in the Madeiran subset and 7.3% in the Canarian subset, with a maximum of 13.3% between a Canarian and a Madeiran species in two cases: *Laparocerus colonnellii*/*Laparocerus calcatrix* and *Laparocerus rugosivertex*/*Laparocerus chaoensis* (from Bugio).

Mean p-distance within subgenera ranks from 2.9% in *Fernandezius* to 7.0% in *Atlantis*, with an overall mean average of 4.6%. Mean p-distance between groups ranks from 5.4% between *Bencomius* or *Belicarius* and *Canariotrox* to 12.0% between *Aridotrox* and *Atlantis*, with a global average value of 9.2% (9.4% in Madeiran groups and 7.6% in Canarian groups). For more information on genetic divergence, see Appendix 2.

### Global phylogenetic tree

In order to facilitate readability and exposition of results, the global phylogenetic tree obtained for the combined set of three mitochondrial markers is displayed in Figure 3 in a collapsed summary form, and thereafter expanded trees of subclades or groups of them are presented individually. The complete phylogram, as well as the 4-gene tree (with 28S rRNA added to the set), are available as supplementary materials to this paper.

In Figure 3 compact subclades are collapsed and labelled after subgenera (established or newly described here). There is one case of paraphyly in the Madeiran clade (one species of *Atlantis* Wollaston, 1854 clusters with species of *Pseudatlantis* Machado, 2008), and the subclade ‘Pecoudius’ has not been divided in subgenera pending further study, so we adopt in this contribution a wide sense (s.l.) for *Pecoudius* Roudier, 1957. Basal nodes are identified with letters for referring purposes.

The Bayesian support is high (BPP > 0.95) in most cases and it rises up from 0.81 to 0.97 in Node N, from 0.94 to 0.99 in *Purpuranius*, from 0.88 to 0.94 in *Fortunotrox*, and from 0.92 to 0.99 in *Machadotrox*, when the nuclear marker 28S rRNA marker is added to the analysis.

A general picture of the estimated ages of the main lineages expressed as mean values is provided in Table 2, with indication of the island distribution of each of lineage (see the complete chronogram with confidence bars in the supplementary files).

For the combined set of three mitochondrial markers we have calculated an overall divergence rate of 3.1 Ma^-1^ by dividing the between groups mean divergence (12.2%) by the average group age (3.98 Ma). In this case, the divergence values between sequences used to obtained the means have been corrected following the Maximum Composite Likelihood model (Tamura et al. 2004), with a Gamma distribution (shape parameter = 0.6) for the rate variation among sites (Appendix 2). Its equivalence in nucleotide substitution rate is 0.0153 Ma^-1^.

The phylogram of the genus *Laparocerus* has two basal branches originating in Node A (age 11.2 Ma): one gives rise to the Madeiran clade (Node M), and the other to the Canarian clade (Node C), which contains also species from the Selvagens and from Morocco. Both clades show sequential polytomies that group together a few or several subgenera with the lineage that splits the next. These solid polytomies represent basal star-shaped radiation events.

### The Madeiran clade

**Figure 4.**
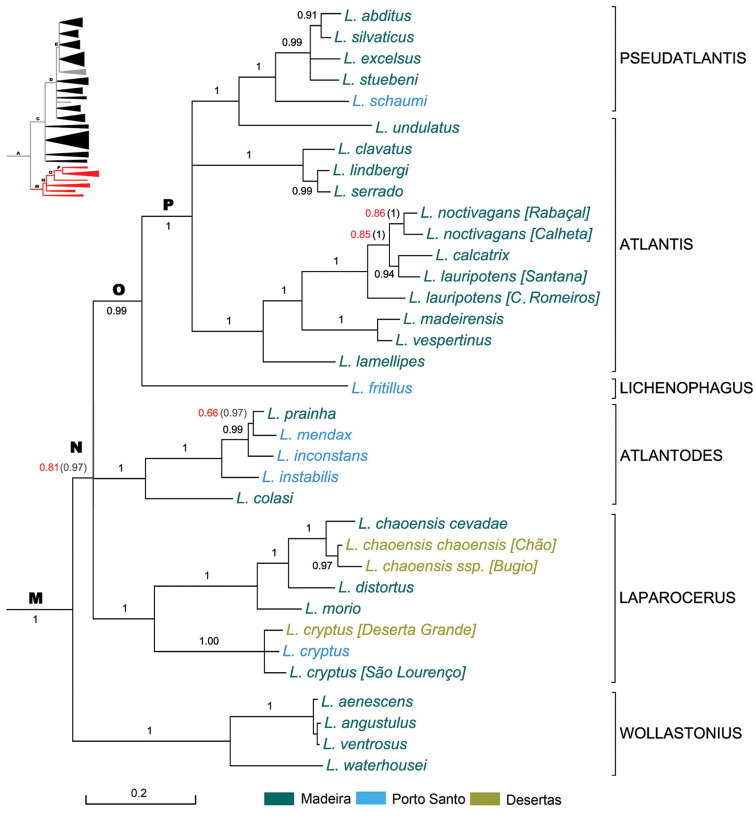
Expanded mitochondrial phylogram of the *Laparocerus* Madeiran clade (Node M). Bayesian posterior probabilities above the branches (in red < 0.95, in brackets when adding 28S rRNA to the analysis). Genetic divergence in scale bar.

The Madeiran clade was presented and discussed in Machado et al. (2008) and Machado (2008a). The addition of the 12S rRNA gene modified slightly the topology and increased the support values, plus the dating performed, and a few extra OTUs justify including here the new version of the tree (Figure 4) and some comments.

The Madeiran clade splits sequentially in time starting with Node M (8.5 Ma), followed by Node N (7.2 Ma), Node O (6.3 Ma), and Node P (5.1 Ma), each giving rise to one or two monophyletic subclades recognised as subgenera and morphologically identifiable; six in total plus *Anillobius* (not included in the tree). Mean p-distance within subgenera ranks from 3.3% in *Wollastonius* to 7.0% in *Atlantis* (average 4.6%).

Subgenus *Wollastonius*, four small sized species (< 4 mm), showed a solid basal position (BPP 1) in the COII–16S rRNA phylogram; clustered with *Atlantodes* when adding the 12S rRNA, and recovers its basal position if the 28S rRNA is added. It is the oldest individual lineage (7.2Ma) in our tree and originated probably in Porto Santo, which was the only emerged island at that times, but it radiated within Madeira much more recently (3.3 Ma). *Laparocerus waterhousei* has been also recorded from Deserta Grande (Wollaston 1854, Roudier 1958) and without analysing specimens from that dismantled islet (we searched for it in 2000 and 2008 without success), it is impossible to infer whether the route followed was from Madeira to the Desertas or in the opposite direction.

Subgenera *Laparocerus* and *Atlantodes* cluster together at Node N (Fig. 4). We missed sequencing *Laparocerus navicularis* from Porto Santo, which is, from the morphological point of view, the sister species of *Laparocerus colasi*, endemic to Madeira. Its absence in the tree could explain the abnormal basal position of *Laparocerus colasi* within *Atlantodes*, a group that presumably started also in Porto Santo. *Laparocerus* (s. str.) is the only subgenus that is distributed in the whole archipelago, and the role of Ponta de São Lourenço, in the extreme east of Madeira, was already discussed in Machado et al. (2008: 423). The fauna of this peninsula in the eastern extreme of Madeira has greater affinity with its extending arc of islets of the Desertas and with Porto Santo (*Laparocerus cryptus*, *Laparocerus schaumi*), than with the rest of Madeira, supporting the hypothesis that the Ponta de São Lourenço was a separate islet that recently fused with Madeira along the valley of Machico.

*Laparocerus undulatus* clusters with *Pseudatlantis* but not with *Atlantis*, the subgenus to which it was attributed by Machado (2008a) based on phenetical consistency of the shape of male metatibiae (Fig. 2E) and the structure of aedeagus. This position is maintained when the nuclear 28S rRNA is added to the analysis. However, in the previously published COII-16S rRNA phylogram, it joined within *Atlantis* with low support (PPB 0.79), and when it was excluded from the analysis the PPB of *Atlantis* raised up to 0.99. If *Laparocerus undulatus* is also removed from our 3-gene analysis, the separate group of *Laparocerus clavatus* clusters with that of *Laparocerus lamellipes*, shaping *Pseudatlantis* and *Atlantis* as monophyletic subgenera. This ‘disturbing’ effect of *Laparocerus undulatus* is likely linked to an old hybrid origin.

The nominal species of *Pseudatlantis* Machado, 2008 have a very characteristic aedeagus structure similar to that of *Atlantodes* and *Wollastonius*, with the gonoporal poach inserted apically (Fig. 2L), not laterally, in what has to be assumed as a case of parallelism, not of homology. Moreover, they do not show sexual differences in the tibiae and have a more rounded body shape, etc, that justified its separate subgenus status. Consequently, we maintain the concept of *Pseudatlantis* and *Atlantis* as established in 2008, including in the latter subgenus the four now ‘outplaced’ species, with a MRCA at 5.1 Ma. This is the only case of paraphyly in subgenera of *Laparocerus*.

After our first Madeiran phylogram was published (Machado et al. 2008), we were able to sequence one specimen of the rare and blind tiny *Anillobius solifuga* Fauvel, 1907 but only for the COII and 16S rRNA, that being the reason it was excluded from the 3-gene phylogram. Nonetheless, taking into account these two markers it clearly falls within the equivalent of Node O (BPP 0.99) in parallel to *Lichenophagus* and *Atlantis*+*Pseudatlantis*, supporting the attribution of *Anillobius* as a subgenus of *Laparocerus* as proposed by Machado (2013). The former species is just a strongly modified *Laparocerus* adapted to endogean life.

The same problem was faced with *Laparocerus hobbit* because only COII and 16S rRNA sequences were available, and both differ only in one nucleotide each from those of *Laparocerus lamellipes*, questioning the validity of the former species. The peculiar characters of the tarsi highlighted in the description (Machado 2008a) may represent a hoxgen-mutation, but mitochondrial introgression or incomplete lineage sorting could as well be a plausible explanation for this case.

*Laparocerus noctivagans* and *Laparocerus lauripotens* are widespread and endemic to Madeira, variable in their morphology, and very difficult to separate. Wollaston described both species in 1854, synonymised them a few years later (Wollaston 1857) and re-established them in 1871 after a careful morphological examination. In our phylogram both species are clearly separated. In addition, specimens of *Laparocerus lauripotens* from the type locality (Curral das Romeiras), in the lee side of the island do not join with specimens from Santana, in the North (incomplete lineage sorting/introgression?). Also, *Laparocerus noctivagan*s from the extreme west region of Madeira are more strongly striated and of black colour, representing perhaps a separate taxon (p-distance 1.6%). This group of *Atlantis* seems to be in active speciation and merit a deep and detailed phylogeographic analysis before taking further taxonomic decisions.

### The Canarian clade

The Canarian clade of *Laparocerus* shows its first radiation event (Node C, Fig. 3) at ca. 7.7 Ma ago, shortly (0.8 Ma) after the Madeiran radiaton. The clade overall mean genetic distance of species is 7.3%, slightly lower than in the Madeiran clade (8.5%), and the mean distance between both clades is 11.0% (net distance 3.1%).

Subsequent radiations in the Canaries occurred at 5.3 Ma (Node D) and 4.6 Ma (Node E), each generating several monophyletic subclades, interpreted here as subgenera (Figure 3). The final outcome of these star-shape branching processes are 13 subgenera in the Canaries versus 7 in Madeira, in coherence with the lesser number of islands and overall minor surface of the latter archipelago. The average within subgenera genetic p-distance in the Canaries is 4.5%, ranking from 2.9% in *Fernandezius* to 5.7% in *Purpuranius*.

Basal lineages from Node C like *Purpuranius* and *Aridotrox* inhabit Fuerteventura and Lanzarote, which are the oldest islands, while younger subgenera like *Belicarius* and *Bencomius* (Node E) are restricted to the most distant and younger Western Canaries. There is a general shift from East to West, with some back-colonisations, which seems to follow the pattern of decreasing island ages and increasing distance to continental Africa associated with the prevailing hypothesis of a hot-spot origin for this archipelago (Carracedo 2011).

*Laparocerus garretai* from the Selvagens has a basal position (2.8 Ma) in one of the groups of species of the subclade ‘Pecoudius’, in agreement with the hypothesis of a Canarian origin for the extant Selvagens Islands’ biota (cf. Machado, 1992). These residual Macaronesian islets are very old in origin (29 Ma), but went through a large submerged phase in the Miocene/Oligocene (Geldmacher et al. 2001), the actual emerged land being younger, approximately 14 Ma.

*Laparocerus susicus*, the only known species from the continent (NW Morocco) join with Canarian endemics in the subgenus *Aridotrox*, and not in a basal position. This would indicate a back-colonisation event at nearly 1.2 Ma, justifying the name of Canarian clade used in this study.

**Figure 5.**
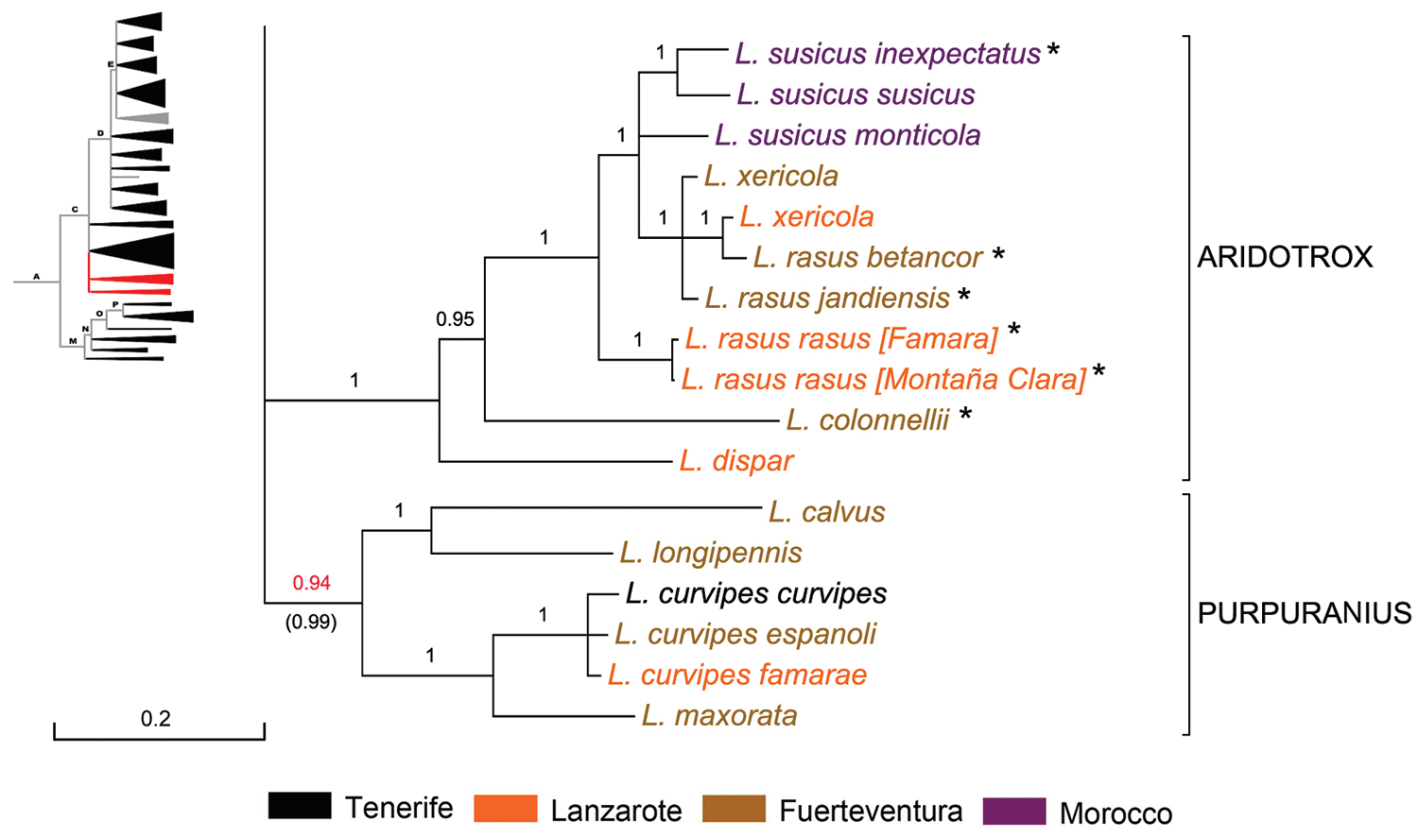
Expanded mitochondrial phylogram of *Laparocerus* Node C: subclades ‘Purpuranius’ and ‘Aridotrox’. Bayesian posterior probabilities above the branches (in red < 0.95, in brackets when adding 28S rRNA to the analysis). Genetic divergence in scale bar. Taxa marked with * have preapically notched male metatibiae.

**Subclade ‘Purpuranius’** (Fig. 5). This subclade (BPP 0.94 (0.99)) of Node C was the first that radiated within the Canarian clade ca. 5.9 Ma ago, and it includes five rather distinct species endemic to Fuerteventura (*Laparocerus fraterculus* not sequenced) and *L curvipes* with one subspecies in Fuerteventura, one in Lanzarote, and another one in Tenerife (*Laparocerus curvipes curvipes*). This is the single case of a *Laparocerus* present simultaneously in the Eastern and the Western Canaries. More striking is its presence in the leeward side of Tenerife and not inhabiting the intermediate island of Gran Canaria, where it may have gone extinct or is waiting to be found. The genetic divergence among the three subspecies ranks from 0.6% (Fuerteventura- Lanzarote) to 1.6% (Fuerteventura-Tenerife).

The mean p-distance of 5.7% within this new subgenus is the highest recorded in the Canarian clade. *Laparocerus calvus* and *Laparocerus longipennis* are adelphotaxa and the oldest Canarian species (4.9 Ma), but they are quite different morphologically (with/without scales and hairs, female ovipositor, body shape, etc.). This may represent the outcome of a long lasting parallel anagenesis or, more likely, that extinction has been most severe in this group, only a relictual set of few species remaining.

Some species of this group dwell in xeric and semi-arid lowland with Chenopodiaceae and *Launaea*, while others are restricted to the sheer summits of the oldest and highest mountains of Fuerteventura (807 m) and Lanzarote (671 m) where remnants of the past thermo-sclerophyllous vegetation persist.

The new subgenus is described in the next section.

**Subclade ‘Aridotrox’** (Fig. 5). This subclade of Node C (BPP 1) radiated ca. 4.8 Ma ago and clusters four species from the eastern Canary Islands and one from western Morocco, all of similar outlook and living in xerophilus mountain or flatland habitats.The Moroccan *Laparocerus susicus* clusters with the Canarian species *L. rasus – L. xericola* with high support (BPP 1) and does not take a basal position within the clade like *Laparocerus dispar* or *Laparocerus colonnelli*. This supports the hypothesis of a back-colonisation from the eastern Canaries to Morocco some 1.2 Ma ago.

The presence of preapically notched male metatibiae in *Laparocerus susicus inexpectatus* is likely to be related to it being a plesiomorphy in *Laparocerus colonnellii* and the group of *Laparocerus rasus* (species bearing this character are marked with an asterisk in Fig. 5). Nonetheless, there are contradictory associations of conspecific taxa within the *Laparocerus rasus* group that suggest incomplete lineage sorting in peripheral isolates and deserve a deeper phylogeographic analysis to clarify their relationships and dispersal routes. The group seems to have started in Lanzarote.

Mean p-distance within *Aridotrox* is 5.3%. The description of this new subgenus is in the next section.

**Figure 6.**
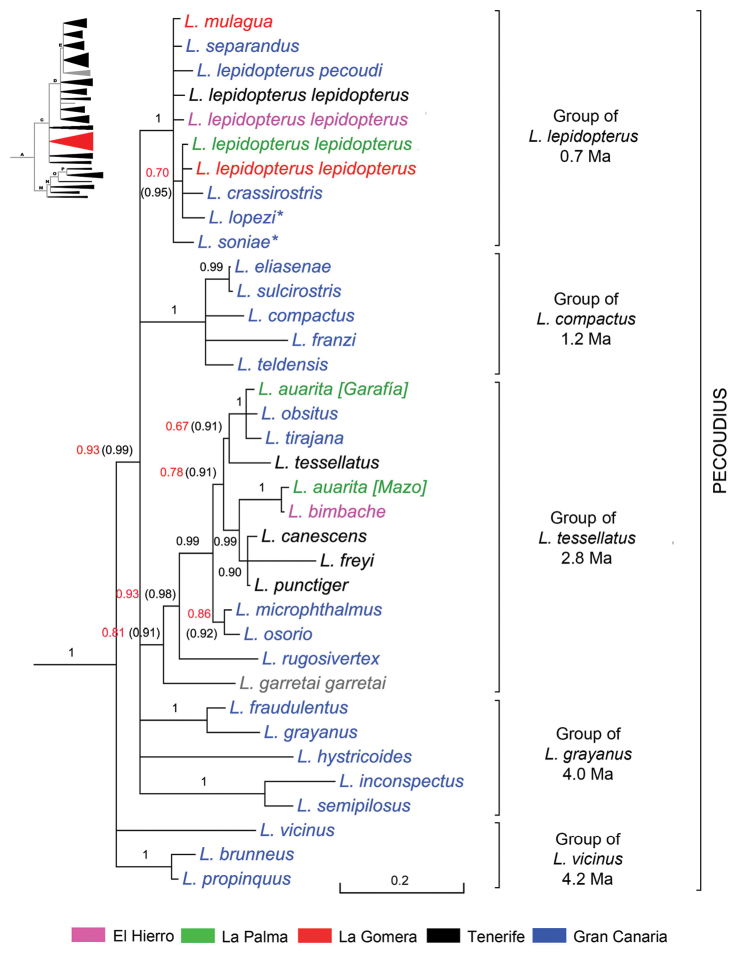
Expanded mitochondrial phylogram of *Laparocerus* Node C: subclade ‘Pecoudius’. Bayesian posterior probabilities above the branches (in red < 0.95, in brackets when adding 28S rRNA to the analysis). Taxa marked with * are subterranean species. Genetic divergence in scale bar. TMRCA of species groups in million years.

**Subclade ‘Pecoudius’** (Fig. 6). This branch of Node C is always fully consistent (BPP 1) regardless of which marker or combination of markers is used. It first radiated ca. 4.2 Ma ago and clusters five morphologically disparate groups of species which could merit a subgenus each. Nonetheless, we opted for naming the subclade after the subgenus *Pecoudius* Roudier, 1957, type species *Laparocerus compactus* Wollaston, 1864, and expand its concept until an expanded morphological and genetic study is conducted.

The evolution of this clade is likely to reflect the convulsive geological history of Gran Canaria, an island with an age of 14.6 Ma that underwent catastrophic volcanic activity between 3–3.5 Ma ago, and a later much milder re-activation of it (Carracedo 2011). The species groups seem to have started at an age (3.3–4.2 Ma) before or close to the volcanic paroxysm, but they radiated only after it ceased: 0.6–2.7 Ma. The ancestral species of these groups are possibly the survivors of the mass extinction, and the somewhat loose position of some of the extant taxa in the tree may be the effect of missing species that disappeared.

*Laparocerus propinquus*, *Laparocerus fraudulentus*, *Laparocerus semipilosus*, *Laparocerus microphthalmus* and *Laparocerus crassirostris* – each belonging to a different ‘Pecoudius’ group have apparently formed in the Tamadaba massif (*vide* Machado 2012a), and have their respective sister species outside, representing a remarkable case of asynchronous parallel vicariance.

Species of the basal group, *Laparocerus vicinus*, *Laparocerus propinquus*, *Laparocerus brunneus*, live in high mountain scrubland and in the understory of the pine forest (e.g. *Cistus*, *Artemisia*). By its aedeagus structure, it may be somewhat related with the group of *Laparocerus tessellatus*, if any. The similarity of aedeagus is more clearly shown in the set of *Laparocerus semipilosus*, *Laparocerus inconspectus*, *Laparocerus grayanus*, *Laparocerus fraudulentus*, and *Laparocerus hystricoides*. They should constitute a second group on a morphological basis even though they do not cluster together in our tree. Species are distributed along the northern coast, in the central mountains, or almost all around the island (*Laparocerus grayanus*). The small monticolous *Laparocerus hystricoides* has preference for *Cistus*, but the other and much larger species are clearly more polyphagous (*Kleinia*, *Rumex*, *Salvia*, *Periploca*, *Argyranthemum*, etc.).

The group of *Laparocerus compactus*, or *Pecoudius* sensu stricto, is formed by five species that dwell in the leaf-litter and do not climb on the vegetation as is the dominant behaviour in *Laparocerus*. Their broadened and compact body, with mesothoracic inter-coxal process abnormally swollen and protruding, is apparently an adaptation to move between the vegetal debris. The extreme case is the dynamic boat-shaped form of *Laparocerus eliasenae*, similar to that of *Laparocerus distortus* in Madeira, clearly useful to push and navigate through the fallen leaves (convergence). The former species and *Laparocerus sulcirostris* live in the laurel forest or humid environments, while the other species dwell in the coastal spurge formations or subhumid sclerophyllous forest. The radiation of the group occurred in the northern side of Gran Canaria at ca. 1.2 Ma, and the last Pleistocenic volcanic activity phase may have played a role in isolating *Laparocerus franzi* in the peninsula of La Isleta, in the NE.

The other two species groups are not exclusive to Gran Canaria. The widely spread species complex of *Laparocerus tessellatus* has *Laparocerus rugosivertex* from Gran Canaria as sister species with BPP 0.93, and both join with *Laparocerus garretai* from the Selvagens Islands with a low BPP 0.81. All these species show the same type of aedeagus, and when adding the 28S rRNA to the analysis, the above referred support values raise up to BPP 0.98 and 0.91, respectively. The group is present in Gran Canaria (four species), Tenerife and the western Canaries (five species) except La Gomera, perhaps reflecting an original association to the pine forest plant community, which is missing in this island. However, they live in intermediate zones of the leeward and windward sides of the islands, in habitats where *Adenocarpus*, *Chamaecytisus* or *Cistus* grow, but also in the interior of the laurel forest. Faria et al. (2015) postulated its origin in Gran Canaria and presented evidence for multiple founding lineages and genetic admixture in their evolution, which is consistent with our results (see disjunct position of two *Laparocerus auarita* specimens). The complexity of this group shows adaptive and non-adaptive radiations that deserve more intense studies, particularly with the nuclear genes (*vide* Machado 2016).

The last group, that of *Laparocerus lepidopterus* (BPP 1; 0.7 Ma), represents an outstanding case in the evolution of *Laparocerus*. It is formed by seven species and one of them, namely *Laparocerus lepidopterus*, is present in the western and central Canaries, with minor morphological variations per island. In the 3-gene phylogram, all ten OTUs sequenced split directly from the clustering node without any pairing. The overall mean p-distance within the group is extremely low: 1.2% (maximum 1.7%) and contrasts with the average of 4.5% within the rest of ‘Pecoudius’. With such a low divergence one would expect a very morphologically homogenous group, but this is only the case in the superspecies *Laparocerus lepidopterus* - *pecoudi* - *separandus*, which are large broad *Laparocerus* with silky hairs on the elytra living in the laurel forest and ecotones with the pine forest (mixed forest). In contrast, *Laparocerus crassifrons* has no hairs and a flattened body adapted to hide below the bark of the Canary pine whose needles it feeds on (it resembles more the pine weevil *Brachyderes rugatus* than a *Laparocerus*); secondly, *Laparocerus lopezi* and *Laparocerus soniae* – species apparently not directly related – are adapted to subterranean life, with reduced eyes, loss of vestiture, and narrower body (the p-distance between *Laparocerus soniae* and its epigean parent *Laparocerus separandus* is 0.7%); and lastly, *Laparocerus mulagua* from La Gomera looks like a half-sized and cylindrical *Laparocerus lepidopterus* with very prominent eyes. Such remarkable morphological differences within the group do not match with the reduced genetic divergence of the mitochondrial loci examined. When adding the 28S rRNA to the analysis, some OTUs cluster with high support values (PPB 0.95) but with little geographical logic. However, cases like *Laparocerus mulagua* joining *Laparocerus separandus* with 1 BPP reflects a direct link from Gran Canaria to La Gomera that has been found also in ground beetle vicariants of the genera *Gomerina* and *Cymindis* (Machado 1992), in the darkling beetle genus *Pimelia* (Contreras Díaz et al. 2003), or in bush-crickets of the genus *Calliphona* (Arnedo et al. 2007). If bad taxonomy is disregarded, there is no simple explanation (admixture, incomplete sorting, paralogy, convergence, etc.) for the high-morphological/low-divergence discrepancies observed in this group other than inadequate phylogenetic information, or that we are missing some unknown underlying genetic process that is worth investigation. The age estimates (0.3–0.7 Ma) obtained for this challenging group is probably unrealistic.

**Figure 7.**
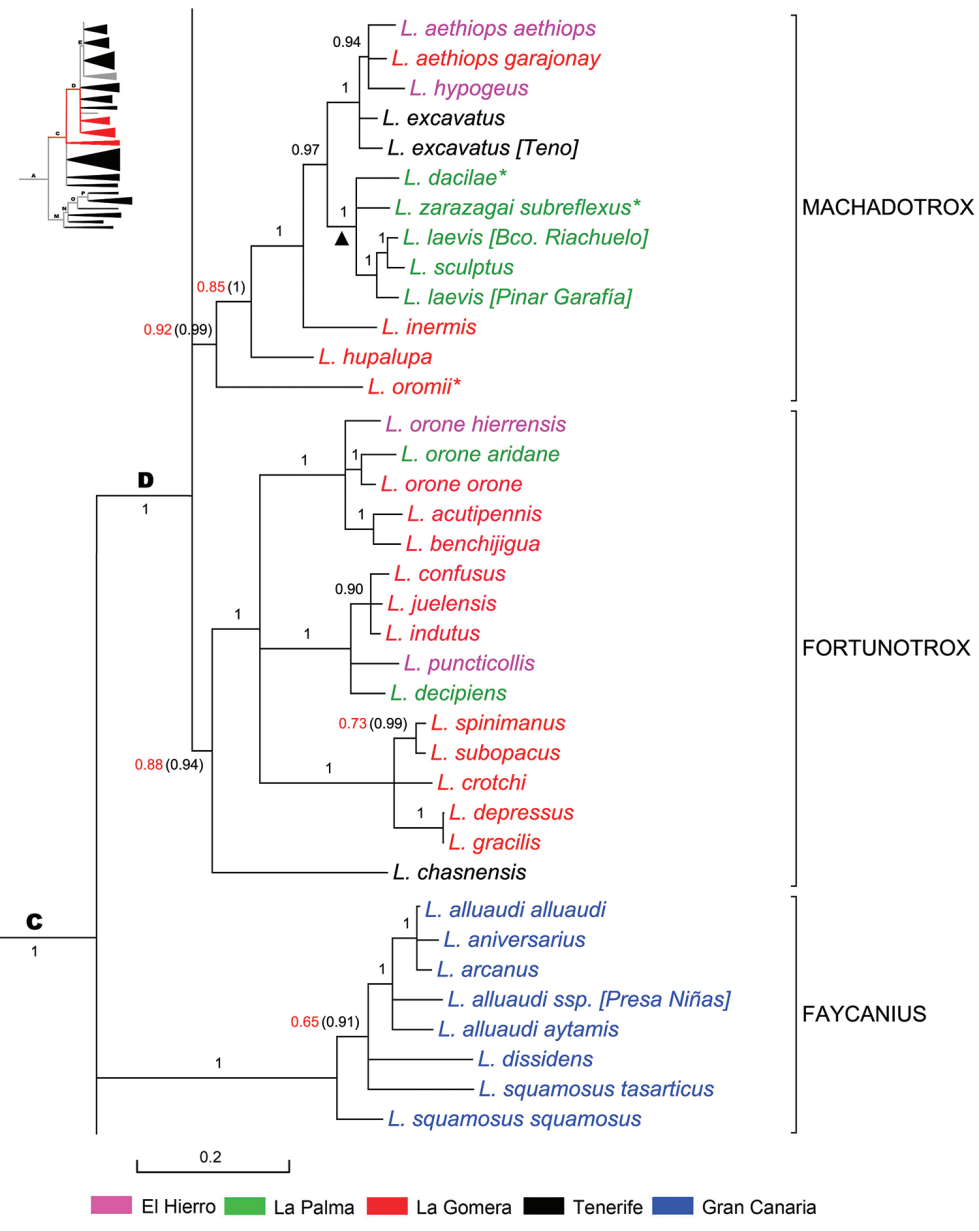
Expanded mitochondrial phylogram of *Laparocerus* Node D: subclades `Faycanius’, ‘Machadotrox’ and ‘Fortunotrox’. Bayesian posterior probabilities above the branches (in red < 0.95, in brackets when adding 28S rRNA to the analysis). Genetic divergence in scale bar. A black triangle (▲) marks the calibration point used for the timetree. An asterisk (*) denotes subterranean species.

**Subclade ‘Faycanius’** (Fig. 7).This subclade of Node C with BPP 1, has five species (two of them with subspecies) all endemic to Gran Canaria. The radiation of *Faycanius* is estimated in 1.9 Ma ago and is likely to have occurred also after the violent volcanic activity (3–3.5 Ma ago) of Roque Nublo Complex which sterilised great part of the island (Marrero 2004).

Species of *Faycanius* live on low plants and bushes (e.g. *Artemisia*, *Argyranthemum*) always in open land avoiding forest and shady areas. They are distributed over the whole island separated in different watersheds or by altitude, but not always.

Mean p-distance within *Faycanius* is 3.5%.

**Subclade ‘Fortunotrox’** (Fig. 7). This subclade of Node D radiated ca. 5.1Ma in the western Canaries forming three well supported groups of species (each with one BPP): that of *Laparocerus puncticollis* (five spp), that of *Laparocerus orone* (three spp., two ssp.), and that of *Laparocerus gracilis* (five spp) exclusive to La Gomera, with the addition of *Laparocerus chasnensis* that is responsible for the low BPP 0.88 of the whole subclade (support increases to BBP 0.94 when the 28S rRNA is added). This latter species, endemic to Tenerife, is basal and the adelphotaxon of the other groups, thus representing the hypothetical source lineage. It is located at mid-elevation in the SW slope near the outcrop of the oldest island shield in Adeje (Roque del Conde), that remained uncovered during the Pleistocene eruptions of the Teide volcano complex (Carracedo and Pérez-Torrado 2013). The mean p-distance within *Fortunotrox* is 5.4%.

The group of *Laparocerus orone* lives in lowland xerophilous vegetation and in the intermediate zone dominated by *Euphorbia* and *Kleinia*, feeding mainly on *Artemisia*, *Argyranthemum* or *Rumex* when it comes closer to the forest zone. It seems to have generated three species in La Gomera following the radial watershed system in the N/NW/W section of the island, and from there the nominal species produced vicariants (subspecies) in El Hierro and La Palma; same habitat.

The group of *Laparocerus gracilis* is richer in species and exclusive to La Gomera, occupying the other half of the island (E and SE slopes), almost separated from the previous group. Species live in the same habitat characterised by dendroid spurges, but also in the remnants of the sclerophyllous forest. Segregation is likely to have taken place in parapatry. Remarkable is the case of *Laparocerus gracilis* and *Laparocerus depressus*, two sister species that live almost in contact in Barranco de La Villa, at different altitudes. Their morphological distinctiveness is not under discussion, larger and depressed body versus smaller and subcylindrical body, but their sequences are almost identical (p-distance 0.07%). We checked and disregarded mitochondrial introgression with nuclear markers. The mitochondrial genes analised simply seem to have not yet differentiated.

The group of *Laparocerus indutus* is linked to the sclerophyllous and the laurel forests in La Gomera and El Hierro, but the vicariant species from La Palma shifted to the high mountain scrub vegetation. *Fortunotrox* seems to have also undergone a mix of ecologically adaptive and vicariant based radiation. The abundance of *Laparocerus confusus* and related species in La Gomera could be an explanation for the absence of a representative of the *Laparocerus tessellatus* group in this island by competitive exclusion (similar size and ecological requirements), but this has not been the case at least with *Laparocerus puncticollis* and L. *bimbache* in El Hierro, where they overlap sharing habitats and often the same plant. In La Palma *Laparocerus decipiens* has apparently shifted to the high mountain domains (> 1800 m) avoiding in part the bulk area of *Laparocerus auarita*.

**Subclade ‘Machadotrox’** (Fig. 7). This subclade of Node D (BPP 0.92/0.99) radiated ca. 4.7 Ma ago in the western Canaries giving rise to 13 species (incl. 2 subspecies). Half of them are adapted to subterranean life (lava tubes, mesocavernous shallow substratum (MSS), and soil): *Laparocerus hypogeus* and *Laparocerus cavernarius* (not sequenced) in El Hierro, *Laparocerus oromii* in La Gomera, and *Laparocerus zarazagai*, *Laparocerus machadoi* (not sequenced), *Laparocerus iruene* (not sequenced), and *Laparocerus dacilae* in La Palma. The epigean species are large forest *Laparocerus* that climb the vegetation to chew the leaves.

The subgenus *Machadotrox* was established by Alonso-Zarazaga and Lyal (1999) to replace the non-available name of *Wollastonicerus* Uyttenboogaart, 1937 (= *Wollastonia* Uyttenboogaart, 1936 non Heer, 1852) and was characterised by the male protibiae being enlarged at the apex both to the interior and to the exterior (fan-like) (Uyttenboogaart 1936, 1937). Our molecular analysis has revealed that this shape of the protibiae is present in several lineages and is not an autapomorphy of the subclade `Machadotrox’. Several species formerly considered *Machadotrox* show up, for instance, in *Faycanius* Machado, 2012, in *Purpuranius* subg. n., or in *Bencomius* subg. n. A good common and unique characteristic of the new concept of *Machadotrox* is the blade truncated form of the female hemisternites (Fig. 2C) with gonostyli placed distant from apex and being very short (not surpassing the hemisternite).

The La Palma endemic *Machadotrox* clustering in our tree (two epigean and two hypogean species) have obviously evolved in situ and therefore were selected as a trustful calibration point for our chronogram, not allowing their most common ancestor to be older than the estimated age of the island (1.72 Ma). The estimated age finally obtained for that node is 1.0 Ma.

The sequenced specimen of *Laparocerus laevis* from the north of La Palma (Pinar de Garafía) does not cluster with a conspecific specimen collected in the type locality and younger part (Barranco del Riachuelo) that joins with *Laparocerus sculptus*. This may represent one more case of incomplete lineage sorting or, perhaps, of poor taxonomy (specimens from the north are on average of a larger size).

The mean p-distance within subgenus *Machadotrox* is 4.8%.

**Figure 8.**
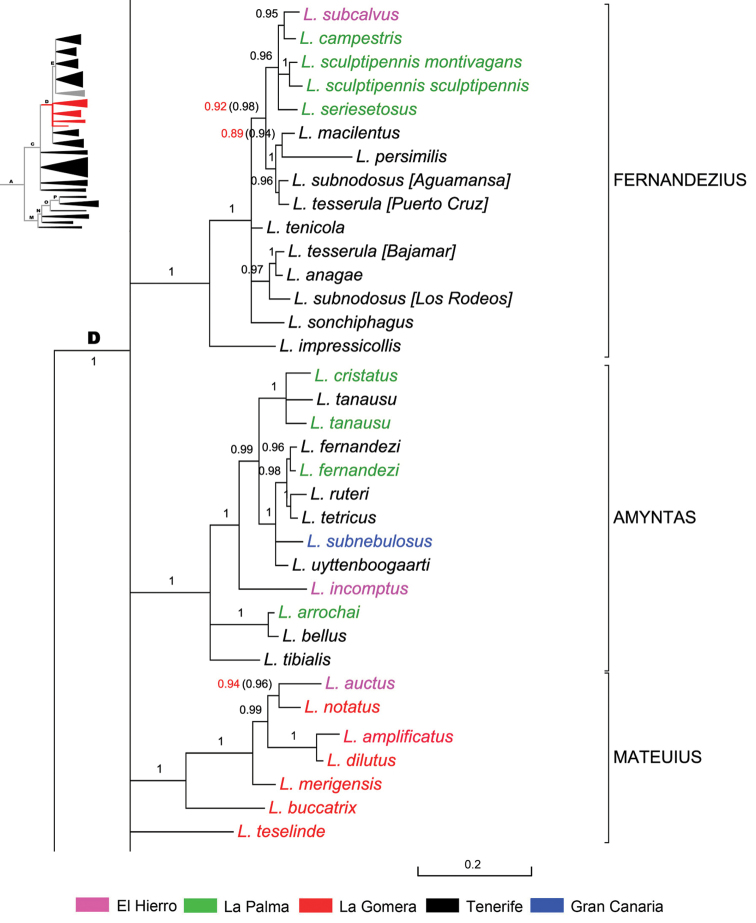
Expanded mitochondrial phylogram of *Laparocerus* Node D: subclades ‘Mateuius’, ‘Fernandezius’, and ‘Amyntas’. Bayesian posterior probabilities above the branches (in red < 0.95, in brackets when adding 28S rRNA to the analysis). Genetic divergence in scale bar.

**Subclade ‘Mateuius’** (Fig. 8). This subclade (BPP 1) of Node D radiated ca. 3.3 Ma ago. Six species cluster together, but *Laparocerus teselinde* does not join the group, branching directly from the Node D. This outside position does not contradict its belonging to *Mateuius*, an attribution that cannot be disputed morphologically, as recently revised by Machado (2015). Except for *Laparocerus auctus* inhabiting El Hierro, all other species are endemic to La Gomera. They live camouflaged in the leaf-litter below scrubs from the lower semiarid zone (*Euphorbia*, *Rubia*, etc.) but *Laparocerus merigensis* and *Laparocerus buccatrix* (and possibly *Laparocerus quadratus*) dwell under trees and shrubs from the sclerophyllous forest or humid laurel forest, where they often find refuge in the dead leaves hanging from woody *Sonchus* species, or under the rossetes of rupicole plants. On the whole, *Mateuius* resemble the *Fernandezius* species which live in the same way and habitats, but on the other islands.

The mean p-distance within subgenus *Mateuius* is 5.0%.

**Subclade ‘Amyntas’** (Fig. 8). This subclade (BPP 1) of Node D radiated at nearly the same time (2.5 Ma ago) as *Fernandezius*. It is equally distributed in Tenerife, La Palma, El Hierro, and absent in La Gomera, but present in Gran Canaria with only the species *Laparocerus subnebulosus*, which, due to its position in the tree, clearly represents a back-colonisation to this island, source of the parental lineage of Node D.

This group seems to have originated in Tenerife, inhabited by seven of the eleven species known. Two of them are shared with La Palma: *Laparocerus fernandezi* and *Laparocerus tanausu*. In the first case a potential introduction to La Palma cannot be disregarded (specimens scarce and located in anthropic sites), but in the second case we postulate a back-colonisation from La Palma to Tenerife with no apparent differentiation. *Laparocerus tanausu* is found in Anaga – much less abundant than in La Palma – where it replaces *Laparocerus tibialis*, and lives also in the offshore uninhabited Roque de Anaga. *Laparocerus tibialis* and *Laparocerus tanausu* are very difficult to distinguish phenetically and the latter should count as a cryptic species.

It is remarkable that *Laparocerus incomptus*, endemic to El Hierro, which is the youngest island (1.12 Ma), has an estimated age of 1.8 Ma and takes a basal position in the subsequent radiation that colonised several much older islands. A plausible explanation for this incongruence is a direct first colonisation of El Hierro from Tenerife, or that *Laparocerus incomptus* derived from the missing *Amyntas* of La Gomera that went extinct (or has not been yet discovered).

*Amyntas* are robust insects, and those species with black dull integument have a somewhat darkling beetle outlook. They are rather polyphagous and distributed in the xeric habitats of the islands leeward side (0–1000 m altitude) and in the more humid coastal zone of the windward side (0–400 m). Only *Laparocerus bellus* and *Laparocerus arrochai* feed on *Jasminum odoratissimum*, a bush species linked to the sub-humid sclerophyllous forest. They are sister species, one in Tenerife, the other one in La Palma.

The mean p-distance within *Amyntas* is 3.8%.

**Subclade ‘Fernandezius’** (Fig. 8). This subclade (BPP 1) of Node D clusters all species of *Fernandezius*, a group with similar ecology and adaptations as *Mateuius*, but with a much younger and wider radiation event (2.2 Ma). It presumably started from Tenerife and reached the Western Canaries, except La Gomera, where its niche seems to be occupied by *Mateuius*. Their morphological convergence to ground life was misinterpreted by Roudier (1957) who described and attributed both subgenera to *Lichenophagus* from Madeira, at that time a separate genus from *Laparocerus*.

In a recent revision (Machado 2015) of the now three subgenera of *Laparocerus*, five new species of *Fernandezius* were described. However, their species status, thirteen species/subspecies in total, all single island endemics, was assigned by the author as an eclectic solution until further and more expanded molecular analyses clarify the real underlying relationships within this complex. We have included type-locality individuals for *Laparocerus tesserula* (Puerto de la Cruz) and *Laparocerus subnodosus* (Aguamansa) in our phylogram and additional specimens from Anaga at the NE of the same island in order to illustrate the problem. Different morphological taxa from a given region group among them and not with their corresponding taxon from other regions. This happens in Tenerife and in La Palma (with *Laparocerus campestris*). Such a repeated geographical influence requires a plausible explanation better than occasional peripheral isolate speciation, admixture in budding species, or a set of cryptic species. New advanced techniques for extracting information out of the nuclear genome, like RAD-seq (Restriction-site associated DNA sequencing), could be possibly the best solution to gain understanding of this species complex, which is also noticeable for the frequency of malformations observed, cases of plausible hybridisation, and the evidence of a successful mutation in *Laparocerus impressicollis* that may represent the initial stage of a new species formation in sympatry (Machado 2015).

The mean p-distance within *Fernandezius* is 2.9%; the lowest in all established subgenera.

**Subclade E & *incertae sedis*** (Fig. 10). The well supported Node E (BPP 1) branches from Node D (5.3 Ma) at ca. 4.6 Ma ago, with Tenerife or La Gomera as plausible origins. It groups four monophyletic subgenera that follow, and a few unplaced species, here considered taxonomically as *incertae sedis*. Judging from their morphology, some of these latter species could be related among them, but others not, and differences are remarkable. They represent lineages that failed in radiating as their sister groups did, or maybe they are the last survivors of once richer lineages that suffered partial extinction.

From the morphological point of view *Laparocerus heres* (with subspecies in La Gomera and Tenerife) is totally independent. It resembles somewhat a narrow *Mateuius* or *Fernandezius* (eyes placed at mid-face, dull integument, etc.) but it does not comply with their other diagnostic characters, and feeds on bushes like *Chamaecytisus*, *Erica*, *Cistus* or *Adenocarpus*. It denotes its own differentiated lineage.

*Laparocerus depilis* Roudier, 1957 is also a remarkable and isolated species, living in the central mixed forest of Tenerife, and unusually rare being a silvicole *Laparocerus*. By shape and tegument vestiture, it resembles somewhat a bald *Laparocerus lepidopterus* and was originally described as a subspecies of this species. However, it has a rather unique aedeagus with the penis strongly sclerotised and almost closed dorsally, ending in an abrupt double square step (in lateral view), with two little acute dorsal flaps on the wall on each side of the ostium. This combination of aedeagal features does not match with any other known *Laparocerus*.

The rest of ‘hanging’ species, basically from Tenerife and La Gomera, share some characters, like silky hairs on the elytra in the case of *Laparocerus ellipticus* and *Laparocerus inflatus* (both strict laurel forest insects), or pubescent coxae and thoracic sternites in *Laparocerus pilosiventris*, *Laparocerus bacalladoi*, and *Laparocerus sanctaecrucis* (all xerophylic species). Only the two latter species cluster as adelphotaxa, and show a p-distance divergence of 3.9%. They live allopatrically in the coastal lee side of Tenerife, one in the S and another in the NE, and their estimated separation point of 2.0 Ma greatly exceeds the age of the mega-landslide of Valle de Güímar (830.000 a f. Carracedo 2011) that could have divided the ancestral population.

*Laparocerus ellipticus* is the only not clearly differentiated species inhabiting five islands: common in La Palma and Tenerife, less common in La Gomera and El Hierro (not sequenced), and very rare in Gran Canaria, where only a few tiny areas of the original vast laurel forests remain. The estimated splitting time is 0.11- 0.17 Ma ago. However, considering that the maximum p-distance of 1.1% between specimens from Gran Canaria and from La Palma falls within the potential limits of local variation, a hypothetical recent introduction by anthropic activities cannot be disregarded. Sticks of laurel forest trees have been traditionally exported from La Palma to Tenerife and Gran Canaria for use in agriculture; La Gomera imported nursery forest plants from Tenerife, etc. This potential shuffle of populations is a question that merits being clarified with a more extensive analysis.

The mean p-distance within the set of *incertae sedis* species is 7.0%.

**Subclade ‘Guanchotrox’** (Fig. 10). This subclade of Node E has a support of BPP 0.94 and 0.98 when the 28S rRNA gene is added. It radiated in four branches ca. 4.2 Ma ago, with a total of 27 species (several polytypic). It is the species richest subgenus of *Laparocerus*, because the subclade ‘Pecoudius’ is not considered a formal subgenus (pending revision). Until now, only the type species *Laparocerus canariensis*, from the upper regions of Tenerife (above 1,800 m altitude) and its vicariant on La Palma *Laparocerus astralis* were attributed to *Guanchotrox*.

The group of *Laparocerus canariensis* inhabits Tenerife (three species), Gran Canaria (three species), and La Palma (one species), all single-island endemics covering all main habitats: the lowland xerophilous scrub formation (e.g. *Kleinia*, *Rubia*), the laurel and sclerophyllous forest (e.g. *Phyllis*, *Aeonium*), the pinewood (e.g. *Sideritis*, *Echium*) and high mountain leguminous scrub (e.g. *Spartocytisus*, *Adenocarpus*). It is a mixed case of vicariant and adaptive radiation.

The group of *Laparocerus tenellus* has four species in Tenerife and one in La Palma that do not all cluster together, but it is morphologically consistent (small, rounded species with strongly mid-constricted rostrum). *Laparocerus tenellus* lives on the north-side of Tenerife, in forest areas, while the other three local endemics are parapatrically distributed in the western and southern lee side of the island. The vicariant of *Laparocerus buenavistae* on La Palma, *Laparocerus palmensis*, is spot-located in the same type of semi-arid habitat, feeding on *Argyranthemum*.

The group of *Laparocerus obscurus* is formed by the nominate species and *Laparocerus dissimilis*; both have radiated within Tenerife, from the coast to the high mountains, covering the whole island. This complex of species with their subspecies merits a detailed phylogeographic study to elucidate the main speciation regions within the largest of the Canary Islands.

The group of *Laparocerus obtriangularis* is the widest spread, with ten species covering all the central and western Canaries. They are mid-sized *Laparocerus* with a shiny or brassy integument. Some species dwell on the bushy vegetation in the forest, and others in the more open scrub formations at lower and intermediate altitudes.

The mean p-distance within the *Guanchotrox* Alonso-Zarazaga & Lyal, 1999 is 5.1%.

**Figure 9.**
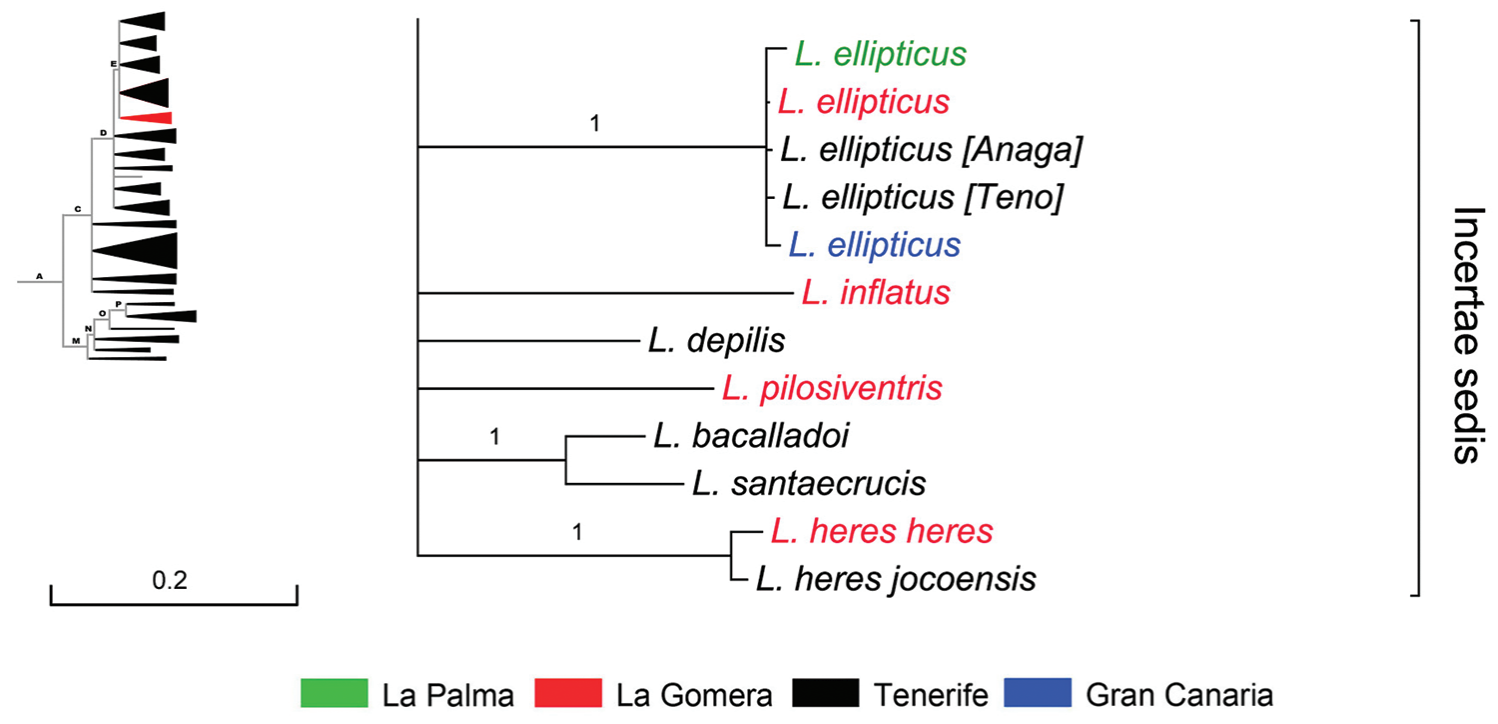
Expanded mitochondrial phylogram of *Laparocerus* Node E: *incertae sedis* species. Bayesian posterior probabilities above the branches. Genetic divergence in scale bar.

**Subclade ‘Canariotrox’** (Fig. 9). This sufficiently supported (BPP 0.96) subclade of Node E (4.6 Ma) branched ca. 3.3 Ma ago into three species groups. It is distributed in the central and western Canaries with a total of 14 species, half of them in Tenerife where it is likely to have originated. We tried hard to find evidence of its existence in La Gomera without success. The absence of *Canariotrox* in this island surrounded by others where it lives is still a mystery, like the case of the *Laparocerus tessellatus* group or *Amyntas*.

The oldest group is that of *Laparocerus occidentalis*. They are all large weevils inhabiting the understory of the laurel and pine forests, and marginal vegetation. Outstanding is the very scarce genetic divergence (p-distance 0.2%) between *Laparocerus aeneotinctus* and *Laparocerus femoralis*, two allopatric species from La Palma, but the morphological differences are clear enough (normal/inflated profemora, etc.) to justify a species or subspecies status and invoke an incomplete lineage sorting of the markers analysed. The four Tenerife species are parapatric, *Laparocerus rugosicollis*, from central parts of the island, being associated with *Laparocerus crassus* from Anaga and not with *Laparocerus aguiari*, its vicariant from Teno. *Laparocerus tauce* lives at high altitude in the scrub formations, on the western flank of the island.

The groups of *Laparocerus vestitus* (open scrub land) and of *Laparocerus inaequalis* (humid laurel forests) are clearly morphologically related, but do not cluster together because of low support (BPP 0.87). Both are present in three islands: La Palma, Tenerife, and Gran Canaria, with morphologically very similar populations within each lineage, and deserve a thorough revision underpinned by a phylogeographical analysis.

Mean p-distance within *Canariotrox* Machado, subg. n. is 3.9%. Its description follows in the next section.

**Subclade ‘Bencomius’** (Fig. 11). This subclade of Node E with BPP 0.94 cluster several species – mainly from Tenerife – originally attributed to *Machadotrox* due to the expanded apex of the male protibiae on both sides. They were removed from that subgenus by Machado (2013), and were pending a new placement.

**Figure 10.**
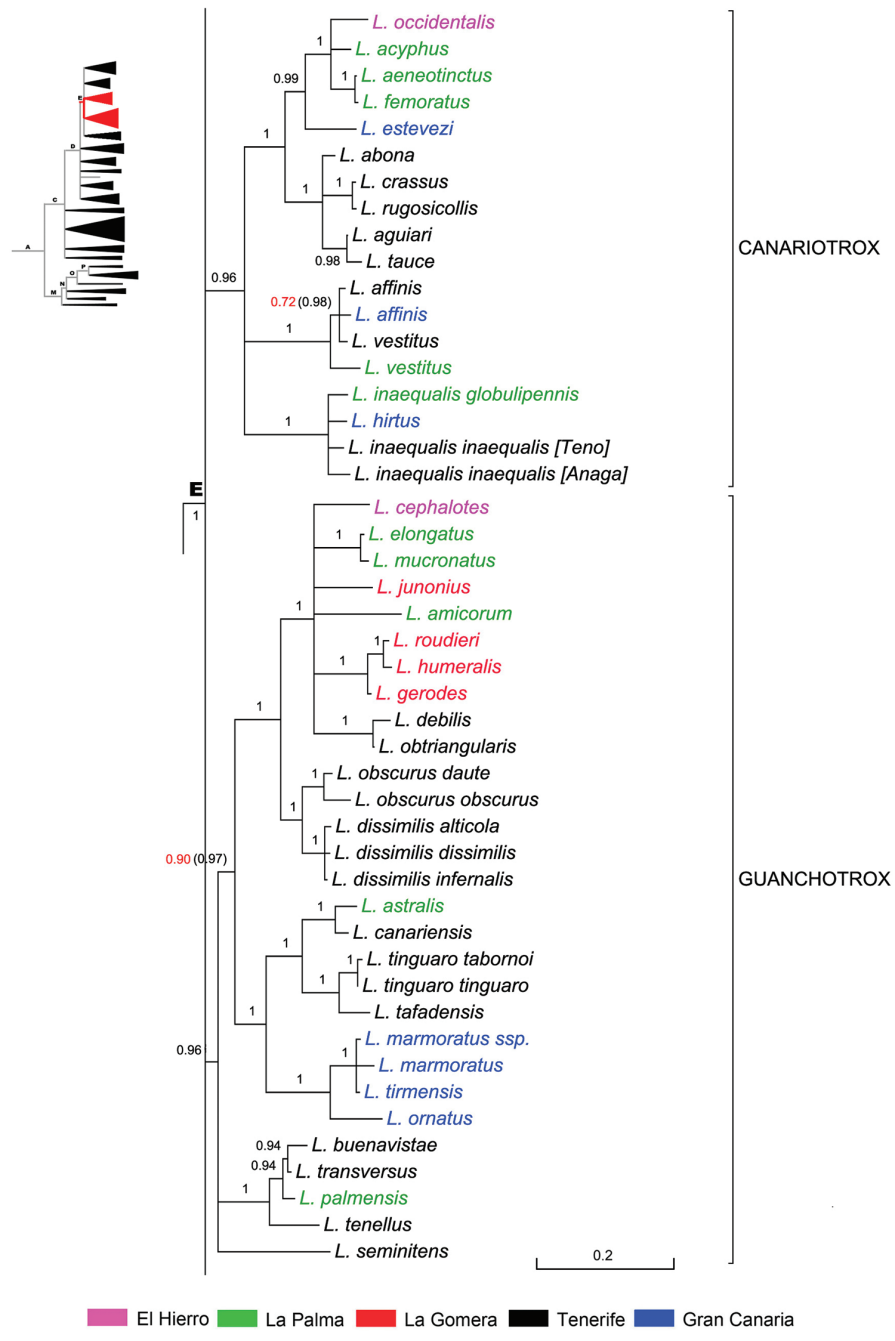
Expanded mitochondrial phylogram of *Laparocerus* Node E: subclades ‘Guanchotrox’ and ‘Canariotrox’. Bayesian posterior probabilities above the branches (in red < 0.95, in brackets when adding 28S rRNA to the analysis). Genetic divergence in scale bar.

**Figure 11.**
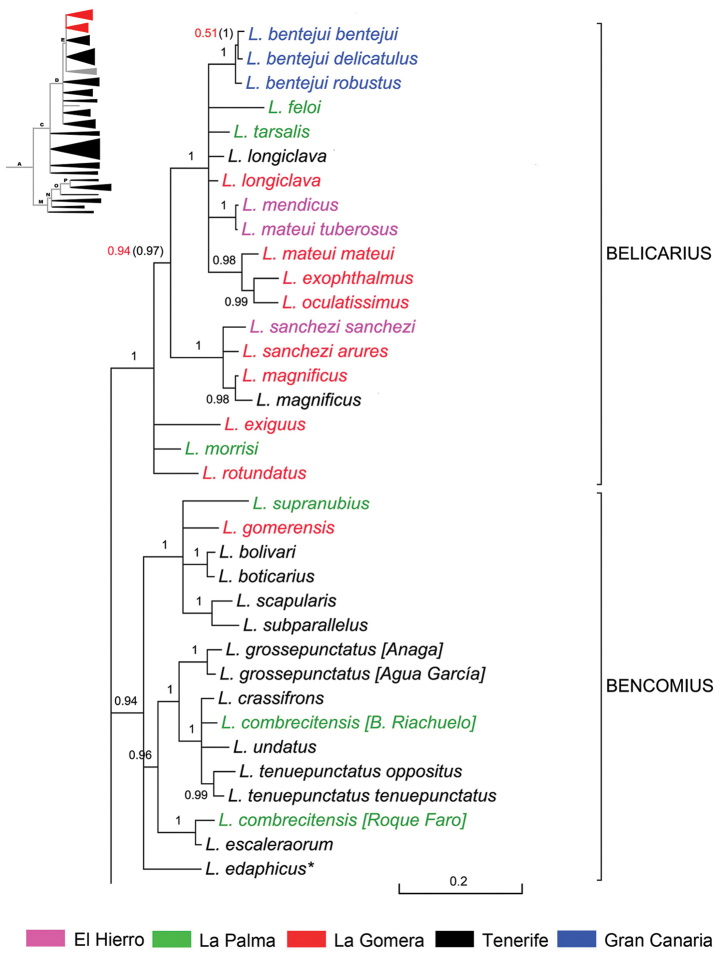
Expanded mitochondrial phylogram of *Laparocerus* Node E: subclades ‘Bencomius’ and ‘Belicarius’. Bayesian posterior probabilities above the branches (in red < 0.95, in brackets when adding 28S rRNA to the analysis). Genetic divergence in scale bar. Symbol * marks subterranean species.

*Bencomius* Machado, subg. n. branched ca. 3.1 Ma ago in two groups of species and the isolated *Laparocerus edaphicus*, which is the only known case of endogean adaptation (eyes and vestiture reduced, etc.) in this subgenus, likely from a common epigean ancestor.

The group of *Laparocerus grossepunctatus* (2.5 Ma) is formed by five species from Tenerife and a putative single vicariant, *Laparocerus combrecitensis*, in La Palma. It is remarkable that a specimen from the latter species collected in the type locality (Barranco del Riachuelo) in the middle of the island clusters (BPP 1) with species from the N and NE of Tenerife and not with another specimen, sharing the same morphology, from the north of La Palma (Roque Faro), which clusters (1 BPP) with *Laparocerus escaleraorum*, endemic to the Teno massif (NW Tenerife). The genetic divergence between the two specimens is 4.2%, which is indeed high, and even higher than 3.9% detected by Faria et al. (2015) within their *Laparocerus* sp 1 from La Palma (= *Laparocerus auarita* Machado, 2016). Multiple colonising events and admixture has been reported for the formation of both *Laparocerus auarita* and *Laparocerus bimbache* from El Hierro (Faria et al. 2015). This could be a plausible explanation for *Laparocerus combrecitensis*, but also the presence of an unnoticed cryptic species.

The group of *Laparocerus scapularis* is younger and radiated 1.5 Ma ago producing four species in Tenerife and single representatives in La Gomera and La Palma. The majority live in open leguminous scrub or on understory plants (e.g. *Adenocarpus*, *Lotus*) of pine woodlands. Only *Laparocerus bolivari* from Tenerife seems to be related to humid forests.

Mean p-distance within the *Bencomius* Machado, subg. n. is 4.1%. Its description follows in the next section.

**Subclade ‘Belicarius’** (Fig. 11). This subclade of Node E with BPP 1 is the last to undergo radiation (estimated 2.8 Ma) among those subclades recognised as subgenera. It is formed by thirteen species distributed throughout the western and central Canary Islands. Some species inhabit two islands, and the concentration of eight species in La Gomera suggests an origin in this island, completing the general shift from East to West already commented upon. Nonetheless, a phylogeographic analysis with more individuals would be needed to confirm this hypothesis and clarify the jumping pattern among the islands (see discussion).

The set of *Laparocerus exiguus*, *Laparocerus morrisi*, and *Laparocerus rotundatus* arise independently from the basal ‘Belicarius’ node due to low support. At least, the first two species, which are very small and live among terophytes on the ground (>1000 m altitude), are clearly related from the morphological point of view, and replace each other in La Gomera and La Palma. *Laparocerus rotundatus* inhabit intermediate zone scrublands in the lee side of La Gomera, feeding on *Rumex lunaria* and *Argyranthemum*.

*Laparocerus sanchezi* and *Laparocerus magnificus* form a clear separate recent group (split 0.64 Ma ago) with subspecies in La Gomera and El Hierro in the first case. *Laparocerus magnificus* is widespread in the north-western parts of La Gomera, but a spot population has been found in the facing Teno massif on Tenerife, in similar habitat – remnants of the sclerophyllous forest – which are not prone to an introduction of anthropic origin. Specimens from both islands look morphologically identical, and the divergence of the individuals sequenced is 1.1%. We postulate a jump from La Gomera to Tenerife followed by no differentiation.

The group of *Laparocerus mendicus* radiated to five islands even more recently (0.8 Ma) and is well supported (BPP 1). *Laparocerus robustus* has three vicariant subspecies in the lofty elevations of Gran Canaria, *Laparocerus longiclava* is present in La Gomera and Tenerife with no apparent differentiation (p-divergence 0.8%), *Laparocerus feloi* and *Laparocerus tarsalis* are endemics to La Palma living in different sides of the island (W/E), and *Laparocerus mendicus* is exclusive to El Hierro. These five taxa have in common the presence of a small preapical tumefaction in the female elytra.

*Laparocerus exophthalmus* and *Laparocerus oculatissimus* are allopatric Gomeran endemics (wet forest/drier open scrubland), share very protruding eyes and are closely related in the tree. However, *Laparocerus mateui mateui* from La Gomera clusters with the previous pair (BPP 0.97) and *Laparocerus mateui tuberosus* clusters with *Laparocerus mendicus*, both from El Hierro (BBP 1). The species is well characterised by elytra beset of big protruding tubercles, a feature that is unique in climbing *Laparocerus* (a parallel case is known in *Rhyncogonus tuberosus* van Dyke, from Tahiti (*vide* Machado 2007a), also a forest living weevil). Sequencing was repeated with the same and other individuals, with equal results. The nuclear 28S rRNA did not show any resolution power either when analysed separately or added to the mitochondrial matrix. However, the nuclear elongation factor (EFα) alone groups both subspecies with BPP 0.92, and then with *Laparocerus mendicus* (BPP 0.86), pointing to a mitochondrial introgression from either *Laparocerus exophthalmus* or *Laparocerus oculatissimus* into *Laparocerus mateui mateui* in La Gomera.

Mean p-distance within *Belicarius* Machado, subg. n. is 3.4%. Its description follows in the next section.

## Descriptions

All the species described before 2012 and species here assigned to the following new subgenera were listed in the *Catalogue of Palearctic Coleoptera* as *incertae sedis* (Machado 2013).

### Subgenus Aridotrox

Taxon classificationAnimaliaColeopteraCurculionidae

Machado subg. n.

http://zoobank.org/5C122A11-A9FB-47DC-A19C-2759DB03E1A9

#### Type species.

**Figure 12.**
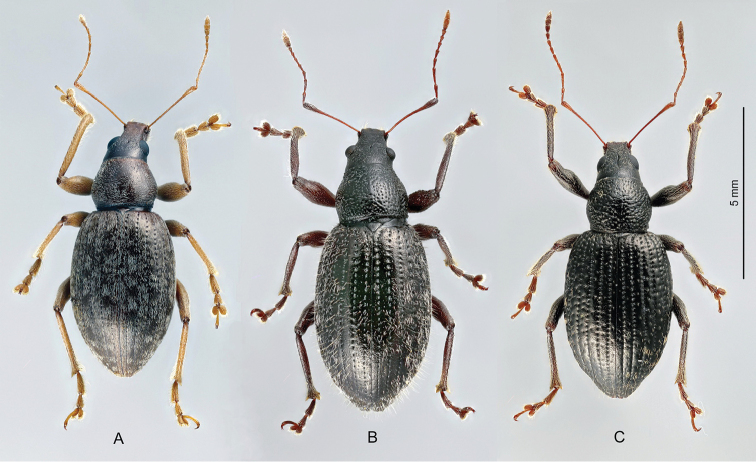
*Laparocerus* subgenus type species. **A** *Laparocerus* (*Aridotrox* subg. n.) *rasus rasus* Wollaston, 1864 **B** *Laparocerus* (*Purpuranius* subg. n.) *maxorata* Machado, 2011 **C** *Laparocerus* (*Bencomius* subg. n.) *grossepunctatus* Wollaston, 1864.

*Laparocerus rasus* Wollaston, 1864, by present designation. Fig. 12A.

#### Etymology.

The name is a combination of the Latin ‘aridus’, meaning arid and the latinisation of the Greek term ‘trōx’, meaning gnawer, applied to weevils. Gender masculine.

#### Species assigned.

*Laparocerus colonnellii* Machado, 2011; *Laparocerus dispar* Wollaston, 1864; *Laparocerus inexpectatus* Machado, 2011; *Laparocerus rasus* Wollaston, 1864; *Laparocerus susicus* (Escalera, 1914); and *Laparocerus xericola* Machado, 2011.

#### Diagnostic remarks.

*Laparocerus* endemic to the eastern Canary Islands and to western Morocco, of small to large size (3.9–8.5 mm) and rather uniform outlook with elongate-ovate elytra in males and ovate in females. The integument is dull and brown with cover of lanceolate scales and no erect hairs (except in *Laparocerus colonnellii*, shiny with long separate hairs). Antennae are slender with thin and briefly capitate scape.

Protibiae straight, with outer apical angle blunt; male metatibiae with a short and deep preapical notch (Fig. 2F) in the outer face shaping the mucro as a flat transversal blade, except in *Laparocerus dispar*, *Laparocerus xericola* and two subspecies of *Laparocerus susicus*. Its presence in *Laparocerus susicus inexpectatus* may be a reason to promote this subspecies to species status once the relationships among the species complex has been clarified.

Aedeagus with several double-rows of denticles in the internal sac of penis (2 apical, 2 median and 4 basal, reduced in *Laparocerus colonnellii* and *Laparocerus dispar*) with a saddle-shaped sclerite (not much sclerotised) in pre-middle position: gonoporal diverticulum tubular and long, not much longer than blind diverticulum. Female gonostyli long and cylindrical placed subapically.

Unique to this subgenus is an isoleucine triplet coding instead of phenylalanine (both non-polar amino acids) in position 51 of the mitochondrial COII gene.

### Subgenus Purpuranius

Taxon classificationAnimaliaColeopteraCurculionidae

Machado subg. n.

http://zoobank.org/CCD69019-A58D-4E32-9B25-469F275F9FDD

#### Type species.

*Laparocerus maxorata* Machado, 2011, by present designation (Fig. 12B).

#### Etymology.

The names derives from ‘Insula Purpurariae’, the Latin ancient name given to the eastern Canaries where Romans and Phoenitians obtained the natural red dye ‘purpura’ from marine molluscs. Gender masculine.

#### Species assigned.

*Laparocerus calvus* Machado, 2011; *Laparocerus curvipes* Lindberg, 1950: *Laparocerus fraterculus* Machado, 2011; *Laparocerus longipennis* Machado, 2011, and *Laparocerus maxorata* Machado, 2011.

#### Diagnostic remarks.

Medium sized *Laparocerus* species (5.0–8.5 mm) endemic to the eastern Canary Islands (Lanzarote and Fuerteventura) with the exception of the nominal subspecies of *Laparocerus curvipes* present in Tenerife. Antennae capitate and male protibiae bent backwards at apical third (maximum in *Laparocerus curvipes*, least in *Laparocerus calvus*); tibial apex may be blunt, incurved or expanded to both sides. Body shape varied and integument either covered with scales and hairs, or totally bare. For instance, *Laparocerus calvus* looks like a bald *Aomus* and has a more robust scape than the other species, while the body of *Laparocerus longipennis* is small and narrow, with normal vestiture of scales and erect setae, but setae on the apex of elytra are shortly bifid at their tip, which is unique within *Laparocerus*.

Penis with two parallel rows of denticles along the internal. Gonostyli long and tubular placed apically on the hemisternites. Female urosternite VIII varied: the apical lamina is transversal in *Laparocerus curvipes*, liguliform in *Laparocerus maxorata*, and in *Laparocerus calvus* spearheaded like in species of *Canariotrox*, showing a case of functional convergence presumably related to oviposition.

Such remarkable morphological differences within this small monophyletic group can be related to long lasting individual anagenetic evolution or that they are a few extant species from a much richer and diverse group in the past. *Laparocerus calvus* and *Laparocerus longipennis* shared a common ancestor ca. 4.9 Ma ago. This is the oldest Canarian group as noted in the previous section, and it may be in the final phase of its taxon cycle (cf. Wilson 1961). Nonetheless, it would also be no surprise if some new *Purpuranius* are discovered in the future.

### Subgenus Bencomius

Taxon classificationAnimaliaColeopteraCurculionidae

Machado subg. n.

http://zoobank.org/7A190562-DDA4-471D-9B87-12BD549E3C44

#### Type species.

*Laparocerus grossepunctatus* Wollaston, 1864, by present designation (Fig. 12C).

#### Etymology.

The name derives from Bencomo, the ‘mencey’ or aboriginal king of Taoro (Orotava Valley) at the times of the conquest of Tenerife. Gender masculine.

#### Species assigned.

*Laparocerus bolivari* Uyttenboogaart, 1957; *Laparocerus boticarius* Machado, 2007; *Laparocerus combrecitensis* Roudier, 1957; *Laparocerus crassifrons* Wollaston, 1864; *Laparocerus edaphicus* Machado, 2008; *Laparocerus escaleraorum* Uyttenboogaart, 1937; *Laparocerus gomerensis* Lindberg, 1953; *Laparocerus grossepunctatus* Wollaston, 1864; *Laparocerus scapularis* Wollaston, 1864; *Laparocerus subparalellus* Machado, 2007; *Laparocerus supranubius* Machado, 2009; *Laparocerus tenuepunctatus* Roudier, 1957 and *Laparocerus undatus* Wollaston, 1864.

#### Diagnostic remarks.

*Laparocerus* endemic to Tenerife, with single vicariants in La Gomera and La Palma. They are in general large, slender, robust, and of piceus colour, with sparse cover of scales or very few and hardly conspicuous (except in species living at high altitude like *Laparocerus crassifrons* or *Laparocerus subparalellus*). The interstriae of elytra beset with a regular row of separate erect whitish setae, which are much reduced only in *Laparocerus undatus* One species, *Laparocerus edaphicus*, is adapted to edaphic life and has reduced eyes.

Head slightly or not depressed dorsally at eye level. Antennae robust, with briefly and thinly capitated scape. Apex of tibiae expanded almost symetrically to both sides (fan-like), depending on the development of the mucro (in *Machadotrox* the outer expansion is much less marked than the inner expansion).

Female hemisternites narrowing apicad (not truncated as in *Machadotrox*) with very few or no setae; gonostyli very short, nipple-like (Fig. 2D), placed laterally at a distance from apex longer than their length. Gonoporal diverticulum of the internal sac of penis shorter than the blind diverticulum.

A detailed morphological study of *Laparocerus undatus* is provided in Machado (2010).

### Subgenus Belicarius

Taxon classificationAnimaliaColeopteraCurculionidae

Machado subg. n.

http://zoobank.org/3A7B1C47-7594-484E-B081-05B9126F3CB7

#### Type species.

**Figure 13.**
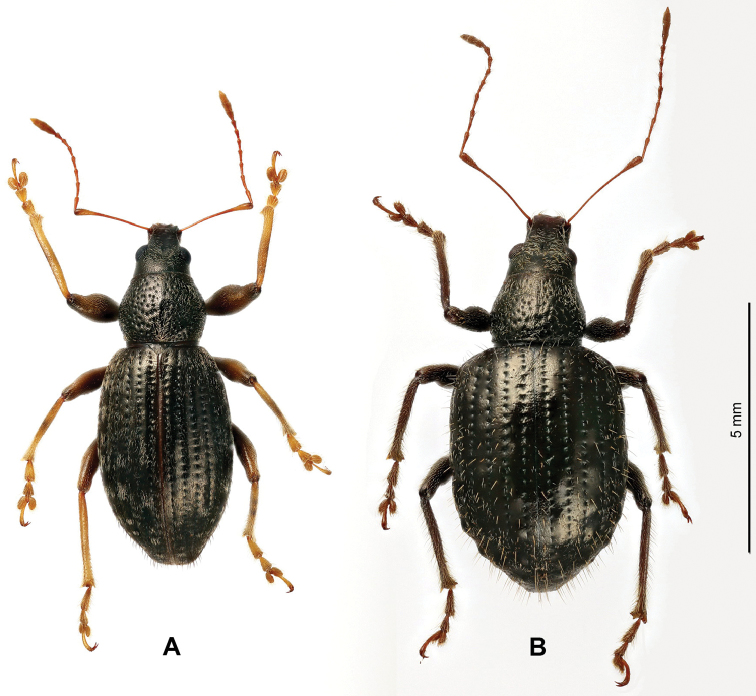
*Laparocerus* subgenus type species. **A** *Laparocerus* (*Belicarius* subg. n.) *mendicus* Wollaston, 1864 **B** *Laparocerus* (*Canariotrox* subg. n.) *inaequalis inaequalis* Wollaston, 1864.

*Laparocerus mendicus* Wollaston, 1864, by present designation (Fig. 13A).

#### Etymology.

The name derives from Belicar, the ‘mencey’ or aboriginal king of Icod at the times of the conquest of Tenerife. Gender masculine.

#### Species assigned.

*Laparocerus bentejui* Machado, 2012; *Laparocerus exiguus* Machado, 2007; *Laparocerus exophthalmus* Machado, 2007; *Laparocerus feloi* Machado, 2009; *Laparocerus longiclava* Lindberg, 1953; *Laparocerus magnificus* Machado, 2011; *Laparocerus mateui* Roudier, 1954; *Laparocerus mendicus* Wollaston, 1864; *Laparocerus morrisi* Machado, 2009; *Laparocerus oculatissimus* Machado, 2007; *Laparocerus rotundatus* Machado, 2011; *Laparocerus sanchezi* Roudier, 1957 and *Laparocerus tarsalis* Machado, 2009.

#### Diagnostic remarks.

*Laparocerus* endemic to the central and western Canaries, varied in size (3.5–8.2 mm) and body shape (slender, ovate, roundish), all having elytra with moderate dull integument beset with abundant black suberect setae, which are usually short, but also of moderate size in some species. In the *Laparocerus exiguus* group (*Laparocerus morrisi*, *Laparocerus exiguus* and *Laparocerus rotundatus*) the body is roundish and of small size (< 5 mm). In *Laparocerus mateui* the density of setae is much lower due to the bulky surface of the elytra, and in the group of *Laparocerus sanchezi* and *Laparocerus magnificus* the integument has additionally long hairs extending to pronotum and head.

Head dorsally depressed at level of eyes. Antennae slender, with thin capitate escape, except in the *Laparocerus exiguus* group where it is more robust and the terminal joints of the funiculum are moniliform in *Laparocerus exiguus* and *Laparocerus morrisi*. Male protibiae have rounded outer apical angle, and in many species the mucro on the inner angle is strongly protruding and sharp, thus the tibia ending hook-like.

The aedeagus has denticles also in the blind diverticulum of the internal sac (except in *Laparocerus exiguus*), which is longer than the gonoporal diverticulum and distally bilobed in the majority of species. The temones are short, nearly 1/3 of the length of the median lobe, except in the *Laparocerus exiguus* group and in *Laparocerus occulatissimus*. Female hemisternites slender with tubular gonostyli inserted subapically.

### Subgenus Canariotrox

Taxon classificationAnimaliaColeopteraCurculionidae

Machado subg. n.

http://zoobank.org/50C6DB37-CDB5-4EE8-A0CE-219BCBC56914

#### Type species.

*Laparocerus inaequalis* Wollaston, 1864, by present designation (Fig. 13B).

#### Etymology.

The name is a combination from the Modern Latin demonym ‘canarius‘ (inhabitant of the Canary Islands) and the latinisation of the Greek term ‘trōx’, meaning gnawer, applied to weevils. Gender masculine.

#### Species assigned.

*Laparocerus abona* Machado, 2016; *Laparocerus acyphus* Machado, 2009; *Laparocerus aeneotinctus* Machado, 2009; *Laparocerus aguiari* Machado, 2007; *affinis* Wollaston, 1864; *Laparocerus crassus* Roudier, 1957; *Laparocerus estevezi* Machado, 2012; *Laparocerus femoralis* Machado, 2009; *Laparocerus hirtus* Wollaston, 1864; *Laparocerus inaequalis* Wollaston, 1864; *Laparocerus occidentalis* Wollaston, 1864; *Laparocerus rugosicollis* Uyttenboogaart, 1937; *Laparocerus tauce* Machado, 2016; and *Laparocerus vestitus* Wollaston, 1864.

#### Diagnostic remarks.

*Laparocerus* of squarish, rounded or elongated appearance, endemic to the central and western Canary Islands. Species of the *Laparocerus inaequalis* group (+ *Laparocerus vestitus* and *Laparocerus affinis*) may be small (4.2–8.2 mm), have shiny or metallic integument, and elytra bearing long silky hairs, while the rest of species (*Laparocerus occidentalis* group) are of larger size (6.2–1.2 mm), with matt integuments, and elytra beset with small setae more or less protruding from the vestiture of scales. Antennae thin and long, with capitated escape. Apex of male protibia incurved with blunt outer angle (except in *Laparocerus vestitus* and *Laparocerus affinis*).

Gonoporal diverticulum of the internal sac of penis as long or longer than the blind diverticulum. Gonostyli tubular inserted at apex of hemisternites. Female terguite VIII ending sharp-pointed (plough-like) and spiculum ventrale (sternite VIII) very robust, spearheaded, with lamina as long as apodeme and with short marginal cirri. This feature is surely related to a special case of oviposition (punching a hard substrate?) and is a good diagnostic character, but not exclusive to this subgenus. Within *Laparocerus*, the same plough-like structure is present in Laparocerus (Purpuranius) calvus, and to some extent in Laparocerus (Atlantis) clavatus. It is also known from other weevil genera.

## General discussion

The purpose of this and the previous study (Machado et al. 2008) was to use molecular information to gain insight of the distribution and relationships of taxa presently attributed to the genus *Laparocerus* in order to assist in its taxonomic revision. The key dilemma is if they should stay as a single genus or if they are better conceived as an aggregate of several related genera of the tribe Laparocerini.

We would favour the single genus option if all *Laparocerus* are monophyletic deriving from a single colonisation event followed by subsequent radiation within Macaronesia. The outstanding morphological differentiation achieved by the local subclades has less importance if we adopt a concept of genus as a unit with biogeographical significance and not just reflecting morphological disparity. Conversely, if the set of *Laparocerus* is the aggregation of several continental lineages that colonised Macaronesia, this evolutive phenomenon could be better expressed in recognising each independent lineage as a separate genus.

If the continental source area is not as large as in other really remote archipelagos like Hawaii or Galapagos, one would expect colonisation events not to be uncommon. The present distance between NW Africa and Fuerteventura is approximately 100 km and this distance was even lower in the past (García-Talavera 1999). Unfortunately, we do not know any *Laparocerus* species – or closely related Entimini – from Africa or the Iberian Peninsula that could represent the ancestral lineage that colonised Macaronesia. *Laparocerus susicus*, endemic to NW-Africa, has been confirmed in this study as a case of back-colonisation. Such back-colonisation events have been reported at least for some plants; e.g. *Aeonium* (Mort et al. 2002), *Convolvulus* (Carine et al. 2004), and *Lotus* (Allan et al. 2004), all of them related to the same Moroccan region known as the ‘Macaronesian enclave’ (Peyerimhof 1946). Consequently, if the ancestral lineage or lineages of *Laparocerus* have not been discovered or went extinct in the continent during the Pliocene-Pleistocene climate changes or even before (*vide* Feaking and de Menocal 2010), the result is that all extant Macaronesian *Laparocerus* will show up as monophyletic, independently of a single or multi colonisation past. Without continental close relatives it is impossible to test monophyly of any insular group (Emerson 2002, Herben et al. 2005), so we have to assume it.

Nonetheless, if we can date the basal nodes in our phylogram and their ages exceed that of the emerged archipelagos – or now sunken seamounts –, the main split(s) must have happened in the continental source area prior to the multiple colonisations. On the contrary, if the split ages fall within the archipelagos ages, a single colonisation becomes a plausible hypothesis, though, we cannot assure it. Our results point in this direction, and without further evidence, we can only gain confidence in such an hypothesis by comparison with other studies in search of coherency, analysing the divergence patterns within the putative genus, and considering the accuracy of our phylogeny and dating estimates.

### Phylogram consistency and cohesiveness

The limitations imposed to our phylogenetic study ‒ by having selected basically only one specimen per species or subspecies ‒ have greater relevance at the tips of the phylogenetic tree, preventing us from detecting cases of mitochondrial polyphyly and paraphyly, or from invoking the appropriate mechanism involved in those contradictory cases that showed up. Funk and Omland (2003) reported species-level paraphyly or polyphyly patterns in 26.5% of 2,319 assayed arthropod species. The phenomenon is taxonomically widespread, and our partial results – and those already registered by Faria et al. (2015) – suggest that it is indeed a common phenomenon in the explosive radiation of *Laparocerus*, and possibly more frequent than in continental taxa. However, our main concerns in this contribution are the basal nodes, where admixture, incomplete lineage sorting and introgressions should have less impact. Adding a third mitochondrial marker (12S rRNA) to our first Madeiran two-gene approach, has increased the support of all basal branches in the general phylogram, ratified with the addition of the nuclear 28S rRNA and its coherent outcome. Therefore, we can trust the first split and subsequent solid basal polytomies (PPB > 0.94) of nodes A-E and nodes M-P as being consistent. These polytomies come from collapsing very close unresolved branchings that ought to represent star-shaped radiations, an evolutionary process that should not be uncommon in oceanic archipelagos if several islands or vacant niches are available for colonisation.

When discussing paraphyly and endemic plant genera of oceanic islands, Stuessy et al. (2014) concluded that genera should be based on cohesiveness, distinctiveness, and monophyly in a wide sense (including paraphyly and holophyly). *Laparocerus* shows strict monophyly, and the overall mean p-distance (3-gene dataset) divergence in the Madeiran (8.6%) and Canarian clades (7.3%) is rather similar. More meaningful for cohesiveness, are the homogeneous divergence values within the named subclades of each clade; 4.8%, and 4.5%, respectively (see Appendix 2). There are no big differences between the two basal clades contradicting cohesiveness in Macronesian *Laparocerus*.

Being the Madeiran and the Canarian clades both monophyletic and sister groups, as they show up, there is an option for establishing two separate genera. However, there are no obvious features for distinguishing or characterising these genera morphologically, and the question of distinctiveness can be even more striking. Species of subgenera from Madeira and from the Canaries like *Lichenophagus* and *Mateuius*, or *Pseudatlantis* and *Aridotrox*, may look more similar among them, than species of sister subgenera from the same archipelago. This is because the explosive radiation of *Laparocerus* (237 species and subspecies) has been triggered both geographically (vicariance radiation) and ecologically (adaptive radiation), producing in the latter case derived forms adapted to different niches – underground environment, leaf-litter, cloud forest trees, high mountains, etc. – which are not free from adaptive morphological convergence. Similar cases have been reported for Canarian *Nesotes* in the Tenebrionidae (Rees et al. 2001a; b). In our results we have included many comments regarding ecology and distribution of species to illustrate these circumstances.

Stüben and Astrin (2010) presented the summarised phylogeny of the Atlantic Clade of Cryptorhynchinae (Curculionidae) using fewer but similar markers to ours (COI and 16S rRNA) and slightly different methodology, although comparable. This Macaronesian group of weevils, which are also flightless, is composed of 95 endemic and 2 introduced species (54 analysed), distributed in the Azores, Madeira, and the Canaries. They concluded that the Canarian and Madeiran archipelagos were colonised by continental Cryptorhynchinae at least seven times. Besides the two introduced species (*Dichromacalles dromedarius* and *Echinoacalles franzi*), four other lineages represent established genera (*Calacalles*, *Onyxacalles*, *Echinodera*, and *Torneuma*), and only one lineage underwent radiation forming a separate Macaronesian clade. This group of formerly *Acalles* is segregated in one new genus for Madeira (*Madeiracalles*) and nine genera and two subgenera within their Canarian clade. This profusion of taxa will reflect shifts to new habitats, like climbing forest trees (e.g. *Dendroacalles*, *Silvacalles*) or switching to different host-plant groups, even if there is only one representative of it (e.g. *Echiumacalles*, *Ficusacalles*, *Pseudodichromacalles*, or subg. *Tolpiacalles*). The argument is that in such endophytic larval dwelling weevils, the shift to a new host plant implies a parallel cladogenesis (Stüben 2000). That may be the case, even for the monotypic genera assuming they will success in the future, and a justification of splitting genera in *Acalles*, but it does not apply to *Laparocerus*, which have free-living larvae and are not host-plant specific.

### Dating confidence

More relevant for testing the single/multiple colonisation alternative hypothesis of the Macaronesian *Laparocerus* is the accuracy of the chronogram obtained in absence of fossils, particularly having acknowledged very disparate evolutionary rates among the different markers and between the different subclades/subgenera of *Laparocerus*. There are many inter-island and intra-island vicariant species that could have been chosen for calibration by taking the age of the island or of the disrupting geological event (e.g. lavaflow age) as a maximum time constraint; the problem lies in establishing a minimum time constraint. The shorter the timeframe, the smaller the uncertainty; but if it is too short, the risk of catching initial increased divergence arises (Penny 2005). Moreover, with vicariant sister species it is assumed that the split happened with or after the colonisation or geological event, and not before.

To circumvent these potential pitfalls, and after cross-validating different potential calibration points, we selected the radiation of *Machadotrox* in La Palma (maximum age 1.72 Ma), two epigean and five modified subterranean species (two sequenced), and tuned its minimum age constraint in function of not allowing the split of Node P ‘Atlantis-Pseudatlantis’ in Madeira to be older than the island age of 5.2 Ma (see methodology).

Stüben and Astrin (2010) used La Palma (2.0 Ma) and Madeira (4.8–5.2 Ma) as calibration constraints for their phylogeny of the Atlantic Cryptorhynchinae, and ended up with 11.6 Ma estimated for the split of Madeiran-Canarian *Acalles*-like genera. This age is very similar to the 11.2 Ma obtained for *Laparocerus*, whereas in the case of Cryptorhynchinae the Madeiran clade (*Madeiracalles*) with 7.3 Ma showed to be younger than the Canarian clade (several genera), with 10.9 Ma.

On the other hand, we have calculated an overall pairwise divergence rate per Ma, obtaining 3.1% for the combined set. These values are higher than the generally accepted standard of 2.34% from Brower (1994), based on uncorrected distances (our rate drops down to 2.3% if we use uncorrected distances), or the 2.1% used by Amorim et al. (2012) as the median value of the range of substitution rates they compiled for Coleoptera (0.7–3.5%). Pons et al. (2010) reported 2.6% just for protein-coding mtDNA.

Bromham and Woolfit (2004) explicitly tested if island radiations can speed up the molecular clock in a range of data sets, including *Tarphius* ironclad beetles, *Pimelia* darkling beetles and *Dysdera* spiders from the Canary Islands, but did not find support for that hypothesis. However, Papadopoulou et al. (2010) designed a careful study to estimate substitution rates in darkling beetles using the mid-Aegean trench separating of the western and eastern Aegean archipelagos (9–12 Ma), and obtained a divergence rate of 3.53% Ma^-1^ for the COI gene and of 2.69% Ma^-1^ when combined with the 16S rRNA gene. They used preferred partitioning scheme and a substitution model selected using Bayes mode, and also removed intraspecific divergence from the analysis as it may introduce higher divergence rates (Ho et al. 2005).

It seems that the rates obtained for different coleoptera or insect groups vary depending on the methodology used and the accuracy of calibration points. Some authors have applied directly the standard mutation rate of Brower, while most of the timetrees recently published for Macaronesia have used BEAST (Drummond et al. 2006), a software program that was originally designed for analysing population level genetic variations and possibly inflates age estimates when dealing only with species representatives and long timeframes. Calibration seems to have been problematic in many cases where very old colonisation events were forced to happen within a given island age, thus falling in tautology.

In this context, our divergence rate estimates can be considered as sound, thus giving additional confidence to the chronogram obtained.

## Colonisation

A mean value age estimate of 11.2 Ma was obtained for Node A, representing the split of the Madeiran and the Canarian clades, an age at which several islands of Macaronesia were already emerged: Porto Santo (14.3 Ma), Selvagens (14 Ma), Fuerteventura (20.2 Ma), Lanzarote (15.5 Ma) and Gran Canaria (14.6 Ma). However, without representatives of the ancestral continental lineage(s), Iberian or African, it is impossible to elucidate if each archipelago was colonised from the continent separately, or if Madeira was colonised first and from that archipelago it jumped to the Canaries. The age estimate for Node M (Madeira) is 8.5 Ma and for Node C (Canaries) is 7.7 Ma, slightly younger.

The ‘radiation delays’ or time-gaps between the first split (Node A) and the first archipelago radiation events is of 2.7 Ma for Node M and 3.4 Ma for Node C, not significantly higher than the average radiation delays 2.1 Ma registered for the other nodes and named subclades, with ages that rank from 0.4 to 5.2 Ma, so as to favour the idea of a split in the continent. Both hypothesis, single or double colonisation of Macaronesia, are thus equally plausible, and from that point of view, setting a genus for each clade would be as sound as keeping both clades within a single genus as we have done for practical reasons (see previous comments on distinctiveness).

Amorim et al. (2012) estimated the colonisation of Macaronesia by *Tarphius* beetles ca. 21 Ma ago, with TMRCA of 7 Ma for the Azores and of 13.5 Ma for the Canarian and Madeiran clade. Hernández-Teixidor et al. (2016) found two clear cut clades of the Cossonine weevil *Rhopalomesites* which should have colonised Madeira and the Canaries in the late Pliocene, ca. 5.3 Ma ago. One clade is associated with *Euphorbia* plants and has one vicariant species inhabiting each archipelago; the other clade has two species in the Canaries, one in Madeira and one in the Azores and Atlantic western Europe (possibly introduced). These species are more generalist (feed on forest trees) and have moved clearly from one archipelago to the other. Stüben and Astrin (2010) estimated the radiation point (TMRCA) in Macaronesia for *Echinodera* at ca. 9.7 Ma ago, 8.7 Ma for *Torneuma* (Porto Santo), 7.1 Ma for Acalles subg. Origoacalles, and 5.3 Ma for *Onyxacalles*. With an estimated split at 7.3 Ma, the species *Madeiracalles portosantoensis* (Stüben, 2002) is the adelphotaxon of the rest of *Madeiracalles* (5.0 Ma) suggesting also that colonisation started in Porto Santo, the same as with *Laparocerus*. The only main difference is that the Cryptorhynchinae Canarian clade (10.9 Ma) is older than the Madeiran clade (7.3 Ma) and two lineages dispersed more recently (1.7 Ma and 0.5 Ma) from the Canaries to Madeira producing respectively vicariant species: *Silvacalles lunulatus* (Wollaston, 1854) and *Dendroacalles ornatus* (Wollaston, 1854).

Colonisation varies greatly depending on the group, and these examples are just a few from the increasing number of phylogeographic studies in Macaronesia, especially with plants (Caujapé-Castells 2011).

**Inter-island dispersal**. At the island scale, it is impossible to assess if any local species lineage of *Laparocerus* derives from a single or from multiple colonisation events without increasing the number and distribution of specimens per species analysed. A few cases tested (cf. Faria et al. 2015, Jordal et al. 2006) suggest that this may have been a common phenomenon in weevils. Nonetheless and despite the fact that our our phylogram is based almost on a single specimen per taxon, it provides a first template of possible colonisation patterns of each archipelago. At least, we can state that there is not a single *Laparocerus* species present simultaneously in Madeira and the Canaries, or any a lineage having vicariants between both archipelagos.

The colonisation of the Madeiran archipelago by *Laparocerus* is likely to have started in Porto Santo, and from there they colonised Madeira and the Desertas, or conversely, with a particular role of the Ponta de São Lourenço in the extreme East of Madeira (Machado 2008a). Humid-forest dwellers (*Atlantis* and *Pseudatlantis*) are exclusive to Madeira, with one case of back-colonisation to Porto Santo in Laparocerus (Pseudatlantis) schaumi (Fig. 4). *Lichenophagus* is the only subgenus present in Porto Santo and Desertas that apparently did not reach Madeira Island, and *Wollastonius* is the oldest lineage in the Madeiran clade, but the extant species formed recently in Madeira and its putative Porto Santo ancestral species is unknown. We have no information of how Porto Santo looked like 8.8 Ma ago, but the ancestral *Laparocerus* was likely not linked to forest or rather humid environments that may have existed in the past.

The tree topology and our timing data (Table 2) suggest that the colonisation of the Canaries by *Laparocerus* weevils follows a shift from East to West (sequential polytomies at 7.7 Ma > 5.3 Ma > 4.6 Ma) in coherence with the pattern of increasing distance to continental Africa and with the decreasing age of the islands originated by a hot-spot of the mantle (Carracedo 2011). Basal subgenera like *Purpuranius* and *Aridotrox* inhabit the eastern islands, which are the oldest, while younger subgenera (e.g. *Bencomius*, *Belicarius*) are restricted to the Western Canaries. This fits the progression rule pattern of island colonisation frequently associated with mantle hot-spot generated archipelagos (Funk and Wagner 1995, Cowie and Holland 2006, Whittaker and Fernández-Palacios 2007, Shaw and Gillespie 2016). However, *Laparocerus* show a more complex pattern than a simple forward stepping-stone progression as reported for some Canarian groups like *Brachyderes* (Emerson et al. 2000), *Hegeter* (Juan et al. 1996), *Nesotes* (Rees et al. 2001a), *Acrostira*/*Purpuraria* (López et al. 2007) or many plant genera (Marrero 2004). If we disregard multiple lineages arriving from the continent, all other possibilities of intra-archipelago colonisation (cf. Funk and Wagner 1955, Sanmartín et al. 2008) can be recognised single or combined in *Laparocerus* subgenera, as we have already highlighted in the comments to each subclade.

**Figure 14.**
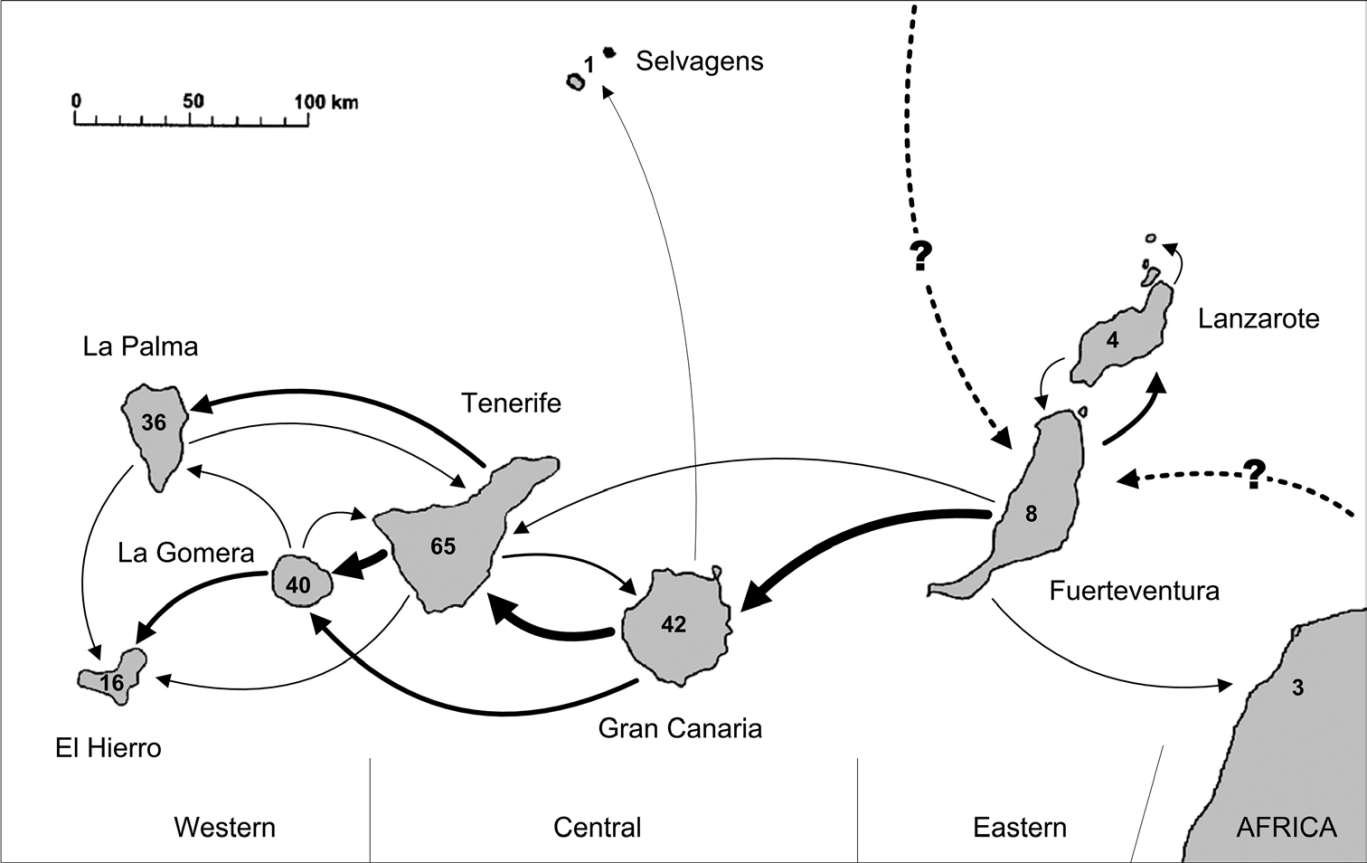
Hypothetical colonisation pathways of *Laparocerus* weevils in the Canary Islands with numbers of species level taxa known from each island. Thick lines, main dispersal routes.

In Figure 14 we illustrate a hypothetical colonisation scenario in the Canaries based on a parsimonious interpretation of our chronogram and known distribution of species. It is very speculative since the directions of dispersal have not been properly analysed with phylogeographic tools. Moreover, extinction must have played an important role masking connections that existed, and we do not know if marine banks that reached the ocean surface may have acted as stepping stones or island refugia in the past (cf. Fernández-Palacios et al. 2010).

In *Laparocerus*, the main progression seems to have moved from Fuerteventura to Gran Canaria, then to Tenerife or to La Gomera, each of the islands acting as successive platforms for dispersal to other islands. This same pattern has been reported, for instance, for *Gallotia* lizards (Cox et al. 2010; Thorpe et al. 1994), whereas there is some disagreement among authors on the order of Tenerife and La Gomera. In *Laparocerus* it seems that *Laparocerus mulagua* of the ‘Pecoudius’ subclade reached La Gomera directly from Gran Canaria, and such an origin cannot be disregarded as an alternative for *Belicarius* or *Machadotrox*. In *Trechus* ground-beetles (16 spp. analysed) one lineage is linked to the laurel forest of Tenerife and La Gomera, and another lineage is a rich species complex in Tenerife and the western islands, including two troglobites, with a single sister species in Gran Canaria (Contreras-Díaz et al. 2007). Unfortunately, *Trechus detersus* from the eastern island was not properly analysed. In *Tarphius* beetles (32 spp.) the diversity in the central and western islands increased due to some intra-island colonisations (Emerson and Oromí 2005), and the putative missing species in Fuerteventura has only been recently discovered in Jandía (Machado 2012b) and is not analysed.

Obviously, in *Laparocerus* the scenario gains in complexity with the profusion of successful internal colonisations and the bonus of several back- dispersals to the parental island (e.g. *Amyntas*, *Belicarius*, *Canariotrox*, and *Guanchotrox*). The result is the set of 196 species and subspecies in at least 13 monophyletic subgenera distributed in several islands, except *Faycanius* and *Pecoudius* s. str. restricted to Gran Canaria, and *Mateuius* which is almost confined to La Gomera but generated one species in El Hierro.

There is not a single subgenus distributed in all the Canaries, and none of the lineages that started in the central or western islands managed to colonise back the eastern islands. *Laparocerus garretai*, endemic to the Selvagens, is morphologically related (aedeagus included) with the group of *Laparocerus tessellatus* and it clusters basally with it within the solid subclade ‘Pecoudius’, suggesting that these old islets were colonised from Gran Canaria.

The absence of *Amyntas*, *Fernandezius*, *Canariotrox* and representatives of the *Laparocerus tessellatus* group in La Gomera is remarkable. When commenting the results by subclades we suggested some plausible explanations, but perhaps the strong reduction in size of this old island, a 38% judging from its ocean platform, may also have played a role in losing fauna. These gaps in distribution are as intriguing as the presence of seven species of *Calathus* groundbeetles in La Gomera and none in La Palma (cf. Machado 1992), whereas this latter island concentrates ten subgeneric lineages of *Laparocerus*. In the case of *Laparocerus*, the youngest islands, El Hierro and La Palma, received almost all available lineages (Tenerife, in the core of the archipelago, has eleven).

**Mass dispersal.** Colonisation of isolate islands has been postulated by active dispersal (flying animals and/or their guts) or by passive wind and sea dispersal. Vectores such as hurricanes and `floating islands’ pushed by rivers are traditionally called upon, and the classic image of a lizard grasping a rafting log used to illustrate oversea dispersal (Carlquist 1965: 19), suggests an occasional but isolate phenomenon. Nothing could be more contrary than drifting hectares or square kilometers of floating debris with all sorts of creatures generated after millions of tons of earth flushed into the sea; plus the effects of the generated tsunami if it hits near islands, wiping part of the coastal areas and, again, adding more rafters to the episode. Such chances, although stochastic, imply massive colonisation essays with many more species and individuals than our deceptive lizard.

Gravitational avalanches are not uncommon in oceanic islands, particularly during rapid growing phases (Whittaker and Fernández-Palacios 2007), and in our opinion their role in shaping the biota of volcanic archipelagos has been undervalued. Seventeen mega-landslides have been recognised in the Canary Islands (Canals et al. 2000), and this counts only for the big and more recent ones, still traceable in the orography of the islands or in the blocks accumulated at the bottom of the sea.

Obviously, island flank collapses may break the continuity of populations and promote vicariant speciation, but more relevant should be their role in island colonisation within a given archipelago.

García-Olivares et al. (in press) present evidence of the colonisation of the island of La Palma by the *Laparocerus tessellatus* lineage departing from the valley of La Orotava avalanche in Tenerife. There are many *Laparocerus* species that could be used for testing homologous cases in this and other islands and unveil the impact of mega-landslides in configuring island biota in the Canaries.

**Origins**. Kim et al. (2008) studied putative rapid radiations of several monophyletic endemic plant lineages in Macaronesia and concluded that the opportunity for island colonisation and successful radiation may have been limited to discrete time periods of profound climatic and geological changes in North African and the Mediterranean. They found three discrete windows of colonisation: in the Middle Miocene (15.3 Ma in *Aeonium*), Late Miocene (8.5 Ma *Sonchus*, 8.2 Ma *Crambe*, 7.5 Ma *Limonium*) and Early Pliocene (3.7 Ma *Echium*, 3.3 Ma *Sideritis*). Plants have more dispersal capacity than wingless weevils, but overall colonisation scenarios should not disagree much among vegetal and animal biotas, especially if the latter are phytophagous. Actually, arrival of the *Laparocerus* lineage could only fit in the Late Miocene window, while the Early Pliocene and its climate changes seem to have had more importance in the diversification of subclades within the archipelagos (average radiation age 3.8 Ma).

The overall picture discussed here fits well the hypothesis of a single lineage colonisation of *Laparocerus* into the Canary Islands. Hence, being the distance to continental Africa nearly 100 km or even less in the past, why are there no more obvious colonisations? Alternative explanations postulated for homologous cases are competition by niche preemption (Silvertown 2004), extensive hybridisation masking late colonisation events (Herben et al. 2005), or extinction of the source lineage in the source area (Whittaker and Fernández-Palacios 2007). The continental lineage of *Laparocerus* may indeed have been a remote Tertiary lineage of Tethian or African Entiminae that did not survive shortly after colonizing Macaronesia. The presence of primitive characters in the larvae, pupae and the genitalia of adult *Laparocerus* (Machado 2010) suggest an old lineage within Entiminae weevils. The lineage itself may be relict, but the extant species living in the archipelago would all be neoendemisms.

## Diversification

Genetic differences among conspecific individuals from different regions or even islands might simply reflect divergence since they became established, with no further cladogenetic significance. This is apparently the case of at least ten *Laparocerus* species that live in more than one island and in the same habitat type (e.g. *Laparocerus magnificus*, *Laparocerus longiclava*, *Laparocerus ellipticus*, *Laparocerus tanausu*, etc.). Their divergence ranks from 0.2% to 1.9%. The proportion of single island endemics, either species or subspecies, is utmost high in *Laparocerus*: 95% (see Table 3), an excellent argument for consolidating the idea of oceanic archipelagos as speciation machines (Rosenzeig 1995).

**Table 3.** Analysis of *Laparocerus* lineages colonisation in Macaronesia (incl. Moroccan enclave). Eight *incertae sedis* taxa have been counted as one lineage.

Island	Lineages (subg.)	Species & subspecies	Shared endemics	Island endemics	Island endemicity	Spp./Subg. Ratio
**Porto Santo**	5	8	2	6	75%	1.6
**Dezertas**	3	4	2	2	50%	1.3
**Madeira**	6	27	3	24	89%	4.5
**Selvagens**	1	2	-	2	100%	2.0
[**Morocco**]	1	3	-	3	100%	3.0
**Lanzarote**	2	4	1	3	75%	2.0
**Fuerteventura**	2	8	1	7	88%	4.0
**Gran Canaria**	7	42	2	40	95%	6.0
**Tenerife**	11	65	8	57	88%	5.9
**La Gomera**	8	40	4	36	90%	5.0
**La Palma**	10	36	5	31	86%	3.6
**El Hierro**	10	16	2	14	88%	1.8
**Macaronesia**	21	237	12	225	95%	11.3

In oceanic islands, species diversification is likely linked to processes of dispersal, vicariance and habitat shifts (Sanmartín et al. 2008). The examples in the Canarian biota are manifold. The daisy genus *Argyranthemum*, for instance, radiated in all islands with two clades, one in the arid zones, pine forest and upper-mountains, and another one in the humid laurel forest and lowland scrub (Francisco-Ortega et al. 1996). In *Laparocerus*, again, the panorama is more complicated as it involves many lineages. Exposing the allopatric speciation patterns and other aspects linked to the ecology goes beyond the purposes of this study and will be duly treated in the monograph of the genus that is in preparation (it includes detailed species distribution). However, some conspicuous patterns inferred from the species phylogeny here proposed are worth commenting to reinforce the idea of congruency with results obtained by other authors.

In phylogeographic studies of other Macaronesian insects, including some Curculionidae: *Brachyderes* (Emerson et al. 2000, Emerson et al. 2006), *Liparthrum* (Jordal et al. 2004), *Aphanarthrum* (Jordal and Hewitt 2004), *Rhopalomesites* (Hernández-Teixidor et al. 2016), strong geographic structuring of population within the islands has been reported, with allopatric patterns that repeat. These studies relate usually to conspecific populations or a few species, but similar patterns can be recognised in *Laparocerus* at species level, with the bonus of redundancy, reflecting the importance of the geological history and ecological diversity of each island in the segregation and diversification of its fauna.

**Fuerteventura and Lanzarote**. There are two *Laparocerus* species exclusive to the oldest massif of Jandía in Fuerteventura (*Laparocerus maxorata*, *Laparocerus calvus*) and another (*Laparocerus rasus*) with vicariants in the central massif of Betancuria, and further north in Lanzarote. This pattern from older to younger territories that joined up to build the present two islands is shared by the darkling beetle *Hegeter deyrollei* (Wollaston, 1864) (Juan et al. 1998) or *Dysdera* woodlouse-hunter spiders (Macías et al. 2013). The scarcity of *Laparocerus* species in Lanzarote may relate to the actual reduced habitat diversity (maximum altitude 671 m), but also to the Pleistocene and recent volcanism that devastated large portions of this island covering them with lava or ash (Carracedo 2011). The interchange of species (e.g. *Laparocerus xericola*) as an effect of sea level changes during the Pleistocene may have had some influence as Fuerteventura and Lanzarote repeatedly connected and disconnected, but nothing really significant in speciation as has been globally suggested (Fernández-Palacios 2016).

**Gran Canaria**. Most subclades present in Gran Canaria show a radiation younger than 3.4 Ma, in contrast to the older age of the island (14.6 Ma). Similar cases reported for several insects, reptiles and plants (Juan et al. 1995, Marrero and Francisco-Ortega 2001, Emerson 2003) have been associated with a hypothetical mass extinction between 4.5 and 3.5 Ma caused by the violent emissions of volcanic agglomerates over great part of the islands (Pérez-Torrado and Mangas 1994). The small group of *Laparocerus vicinus* within subclade ‘Pecoudius’ shows a basal position and may represent direct survivors of the original colonising lineage, and the rest of the species are possibly the result of a generalised recolonisation process from local refugia as has been postulated for plants (Marrero 2004).

The group of *Laparocerus compactus* (= *Pecoudius* s. str.) and *Faycanius* developed in the island and radiated ca. 1.2 and 2.0 Ma ago, respectively. The group of *Laparocerus grayanus*, somewhat loosely related in our phylogram, is also a local lineage. Other lineages, like *Guanchotrox*, which produce three species, and *Amyntas*, which did not radiate locally, arrived from the neighbour islands of Tenerife at 1.3 Ma and 0.5 Ma ago, respectively. Localised species with more amply distributed vicariants seem to concentrate in the areas not affected by the volcanic cataclysm, like in the Tamadaba massif at the NW (*Laparocerus crassirostris*, *Laparocerus grayanus*, *Laparocerus propinquus*, *Laparocerus microphthalmus*, etc.), La Isleta (*Laparocerus franzi*), the ravines of Fataga (*Laparocerus dissidens*, *Laparocerus anniversarius*), Tasarte-Tasartico complex (*L squamosus tasarticus*), etc. The role of the last eruptive cycle (< 2.8 Ma) in their vicariance should be analysed in a thorough phylogeographic study, as some distribution patterns agree with those found in the recent expansion (1.9–2.3 Ma) of *Brachyderes rugatus* on this island (Emerson et al. 2000).

The Gran Canaria record ratio of seven species per lineage shown in Table 3 is possibly inflated. If the subclade ‘Pecoudius’ would be separated in five subgenera, for instance, the ratio would reduce to 4.2.

**Tenerife**. The central and major part of this high island (3.717 m) is covered by Pleistocenic materials produced by the Teide-volcano complex. From the old original shields three main parts remained untouched and are intensively eroded: Roque del Conde (Adeje) in the SW, the Teno massif in the NW and Anaga massif in the NE (Carracedo and Pérez-Torrado 2013). Allopatric Teno-Anaga vicariance in species have been copiously reported and studied in many groups (Cobolli Sbordoni et al. 1990, Emerson et al 1999, Moya et al. 2004, Macías et al. 2013, etc.), in most cases, being related to separate proto-islands that fused later with the volcanic activity of the central parts (Ancochea et al. 1990; Ancochea et al. 2006). With such a scenario we would expect former (proto) island endemics to be much older than 1.5 Ma. Conversely, in a ‘kipuka scenario’ as postulated by Machado (1976: 393), lineages start to diverge once isolated in these refugia due to the volcanic activity, and should be younger than 1.5 Ma. The splitting age of *Laparocerus* with sister species, subspecies or populations in Teno and Anaga are 0.5 Ma in *Laparocerus aguiari*/*Laparocerus crassus*, 1.5 Ma in *Laparocerus tenicola*/*Laparocerus anagae*, 0.6 Ma in *Laparocerus obscurus daute*/*Laparocerus obscurus obscurus*, 0.6 M in *Laparocerus inaequalis* Teno/Anaga, etc. Our results do not agree with the proto-island hypothesis, despite its popularity. Moreover, differentiation of the species or subspecies in the intermediate zone should be even younger, and that is the case, for instance, for *Laparocerus rugosicollis* derived from *Laparocerus crassus* in Anaga (0.1 Ma), or subspecies of *Laparocerus dissimilis* derived from *Laparocerus dissimilis infernalis* (0.2 Ma), a spot-endemic to the Adeje outcrop. Obviously, the colonisation/recolonisation of the intermediate zones could have started from any of the refugia.

There are other geographical speciation zones that can be inferred from *Laparocerus* species, like an eastern and a western sector within Anaga, the south and southwestern leeside of Tenerife, the Teno Bajo platform, the summits of the islands (>1800 m a.s.l.) etc. They ought to be related to eruptive events or ecological differentiation (e.g. the summit environment), but also to the several mega-landslides that occasionally have wiped out a large part of the island in the last million years (Orotava Valley, Icod Valley, Güímar Valley, etc.).

**La Gomera.** Despite having missed some lineages that are present in surrounding islands, La Gomera has a high ratio of 5 species per lineage: *Belicarius*, *Fortunotrox* and *Mateuius* have radiated profusely in a blend of geographical and ecological circumstances. The geographical stamp shows a radial pattern of valley isolated species in each major watershed (e.g. *Laparocerus orone*, *Laparocerus acutipennis*, *Laparocerus benchijigua*) while the habitat segregation is clearly linked to the laurel forest, the lowland succulent belt, and an obscure role of the sclerophyllous forest. The niche shift from the semi-arid belt to the humid laurel forest mirrored by Cryptorhynchinae (Stüben and Germann 2005: 48) is clear in subgenus *Mateuius* as inferred from the basal position of *Laparocerus teselinde*, which feeds on dead leaves of *Euphorbia* and *Rubia*. In other groups it is not so obvious and species may have been associated with the sclerophyllous forest vegetation. La Gomera lacks pine forest and high mountain scrub communities, which reduces the options of niche shifts.

**La Palma**. The geological structure of this young island (1.72 Ma) is very marked, with its north part being older, and the south being younger and still growing (last volcanic eruptions in 1949 and 1971). The hypothesis that each part has been colonised from different source regions of Tenerife is gaining credit with our data on *Laparocerus combrecitensis* and those of *Laparocerus auarita* published as *Laparocerus* sp1 by Faria et al. (2015). The expansion corridors of the Canary pine weevil, *Brachyderes rugatus* shown by network-based analysis of intraspecific DNA (Emerson et al. 2006) can be roughly recognised in the distribution of *Laparocerus*. Sister species show north/south splits, but also east/west (humid/dry) splits. There are also some intermediate ravines (e.g. La Galga) in the NE or mega-landslides (Cumbre Nueva) that may have played a special role in diversification within *Amyntas* and *Belicarius*. The summits above 1900–2000 m offer the same type of habitat as in Tenerife, and La Palma vicariants share the same ecology (e.g. *Laparocerus astralis*, *Laparocerus supranubius*) as their sister species (windborne dispersal?). Some subgenera have colonised the islands with more than one lineage (e.g. *Amyntas*, *Guanchotrox*, *Canariotrox*) and ecological shifts have also played a role in diversifying groups like *Fernandezius* (succulent bush/laurel forest) or *Machadotrox* (pine forest/laurel forest). Outstanding are the five *Machadotrox* species adapted to the underground environment (Machado and García 2010), in a combination of ecological and geographical radiation (see further comments under ‘Habitat shifts’).

Considering the composition of the *Laparocerus* fauna of La Palma, ten lineages, 36 species, it looks as if there has been an initial explosion of subspecies and species fostered by sequential redundant colonisations, vicariance events, peripheral isolates, and niche shifts. It is likely that after such a `boom and bust’ speciation phase the fauna will settle with time as a more reduced set of ‘winners’ tuned by extinction, dilution after secondary sympatry, or admixture, once the island geologic ‘turbulence’ has calmed. This view agrees somewhat with the island immaturity-speciation pulse model of island evolution (Whittaker and Fernández-Palacios 2007) with perhaps a greater role of geological turbulence, e.g., eruptions, island flank collapses, in triggering natural speciation essays.

**El Hierro.** As in the case of La Palma, El Hierro has been colonised by ten lineages, missing *Bencomius* but adding *Mateuius*. However, with only 16 island endemics, it has the lowest species per lineage ratio (1.6) in the Canaries, likely related with its younger age (1.12 Ma), smaller size, and having lost great part of the volcanic edifice in three mega-landslides (Carracedo et al. 1999). El Hierro *Laparocerus* fauna is mostly composed of single Gomeran or La Palma vicariant species (e.g. *Laparocerus auctus*, *Laparocerus cephalotes*, *Laparocerus occidentalis*, etc.). Local radiation seems to have happened only in *Machadotrox*, starting with the epigean *Laparocerus aethiops* line from La Gomera that split in a local epigean subspecies and two strongly adapted subterranean species which are separated by the rim of El Golfo mega-landslide: *Laparocerus hypogeus* in the north, living below humid laurel forest, and *Laparocerus cavernarius* (not sequenced) in the south, found in a volcanic cave under an arid environment.

### Habitat shifts

In the previous paragraphs, a combined history of multi-vicariant and ecology-driven speciation and radiation events is envisaged for *Laparocerus*. Habitat shift is, among other factors, a process invoked for radiation success in oceanic islands, especially if niches are vacant (Juan et al. 2000, Emerson 2002). *Otiorhynchus* weevils are likely to represent the ecological counterpart of *Laparocerus* in neighbouring continental habitats, but no native species are known from the Canaries. Other potentially competing Entiminae are a few *Sitona* species that feed on leguminosae (*Adenocarpus*, *Chamaecytisus*, *Cytisus*) or *Bituminaria* (some are possibly introduced) and the large and endemic *Herpisticus* weevils (five species) present in semiarid habitats in all islands. More prone to sharing part of the feeding niche of *Laparocerus* are the darkling-beetle *Nesotes* (19 species and two subspecies), that climb the vegetation at night to feed, and have radiated similarly to *Laparocerus*, but with less success (Rees et al. 2001c). However, in these putative competitors the number of individuals per plant is far from being as high as it can be in *Laparocerus* (Machado and Aguiar 2005).

It is noticeable that if several sympatric *Laparocerus* species are beaten from the same plant – up to five species – they normally belong to different subgenera. A clear exception are three species of *Purpuranius* in Fuerteventura (to be discussed later), and it may happen occasionally in *Bencomius* (*Laparocerus undatus* and *Laparocerus grossepunctatus*) and in *Belicarius*. This empirical evidence suggests that several lineages underwent the same habitat shifts, and that competition among *Laparocerus* seems not to be a serious problem.

**Original habitat.** Excepting water and non-vegetated sand, lava and ash fields, *Laparocerus* are present in all natural habitat types of the islands: dune ecosystems (only in Madeira *Laparocerus mendax* and *Laparocerus prainha*), xeric *Launaea* steppes, semi-arid succulent shrub land, sclerophyllous forest, azonal cliff vegetation, laurisilva (laurel and heath forests), pine forest, high mountain scrublands and grasslands (including meadows in Madeira), and the subterranean environment. However, without knowing the ecology of the ancestral continental lineage and the paleo-environment of the islands in the late Miocene, it remains speculative to assess which was the original habitat.

The gradual closure of the Panama Straight (3.8–3.4 Ma ago), with its final closure nearly 2.5 Ma ago, modified the Gulf-Stream North Atlantic circulation which is responsible for the humid trade winds that arrive at Madeira and the Canaries, as well as for the colder sea-waters that contribute to ameliorate the climate extremes in these archipelagos (Haug and Tiedemann 1998, Molnar 2008). The trade-winds opened the ‘ecological window’ in Macaronesia for habitats like laurel forests. However, this climatic change also transformed the character of the vegetation in Africa and the Mediterranean (Blondel and Aronson 1999). Fossil gastropods found in Fuerteventura and dated in 4.8 Ma point towards a paleoclimate of oceanic-equatorial character (like in the Gulf of Guinea) much warmer than at present (Meco 2008). If *Laparocerus* arrival to the Canaries has been postulated at about 7.7 Ma ago, we simply do not know the habitat types available in the Canary Islands. It is easy to presume that they were not as dry as one tends to imagine judging from the present state. A recent reconstruction of the climatic conditions of Europe during the Tortonian (7.2–11.6 Ma) reveal mainly humid and subhumid summers, and no trace of a summer-dry Mediterranean climate even along the southern coasts (Quan et al. 2014).

The oldest Canarian *Laparocerus* group is *Purpuranius* (5.9 Ma) and its ecology could give some clues about the original habitat. *Laparocerus longipennis* dwells in arid scrubland on Chenopodiaceae (e.g. *Salsola vermiculata*), but *Laparocerus calvus*, *Laparocerus maxorata* and *Laparocerus curvipes* live sympatrically in the summits of the old Jandía massif (807 m), feeding mainly on the same woody plant, *Asteriscus sericeus*. There is a narrow habitat with relictual soil containing alophanes and maintained by the humidity of the tradewinds. The presence of isolated specimens of tree species known from the sclerophyllous forest and laurisilva (Rodríguez Delgado 2005) suggest that forests may have had a much larger distribution in the past, when the climate was more humid and the island much higher. An ancestral association of *Laparocerus* with some kind of forest cannot be disregarded.

**Sclerophyllous forest**. The sclerophyllous forest of the Canary Islands – also termed thermophyllous forest – is considered a species rich community of Mediterranean origin that occupied the transition zone between the succulent belt and the more humid laurel-forest, at 0–200 m and 500 m altitude in the windward side of the islands, and 300–500 and 700–900 m in the lee side (Fernández-Palacios et al. 2008). Unfortunately, we do not know what it looked like in natural conditions, as it has been almost completely destroyed by anthropic pressure.

Several *Laparocerus* species feed on plants and shrubs associated with this type of forest that survive scattered in cliffs and ravines (e.g. *Convolvulus floridus*, *Carlina salicifolia*, *Bupleurum salicifolium*, *Maytenus canariensis*, etc.). Good examples are *Belicarius* species in La Gomera (e.g. *Laparocerus crotchi*, *Laparocerus subnodosus*, *Laparocerus gerodes*, *Laparocerus humeralis*, *Laparocerus magnificus*) or Laparocerus (Amyntas) bellus from Tenerife and its vicariant in La Palma, *Laparocerus arrochai*, which feed on *Jasminum odoratissimum*, the only apparent case of monophagy recorded in *Laparocerus* (Machado 2003, 2009b) . However, the sclerophyllous forests are supposed to have emerged in the Mediterranean under hot dry summers and cool wet winters approximately 3.4 Ma ago (Jiménez-Moreno et al. 2010), under the influence of the climate change triggered by the closure of the Straight of Panama and well after the Messinnian Salinity Crisis in the late Miocene. In addition, *Amyntas* and *Belicarius* are recent lineages (< 2.8 Ma), and the number of characteristic plant components of this community that are not accepted as food by *Laparocerus* is much larger and significative: *Bosea*, *Dracaena*, *Juniperus*, *Lavatera*, *Marcetella*, *Olea*, *Phoenix*, *Pistacia*, *Rhamnus*, *Ruta*, etc. If there was an original association of *Laparocerus* with forest habitat, it probably was of a different type, and their presence in the sclerophyllous forest reflects a habitat shift or a survival strategy in the case of Fuerteventura.

**Laurisilva**. The Macaronesian laurisilva, traditionally termed ‘monteverde’ in the Canaries, covers laurel forests, heath forests and all their variations, but is generally referred to as laurel forest, in a wide sense. Stüben and Germann (2005:48) postulated for Cryptorhynchinae a start in the conspicuously more arid habitats of the coastal succulent belt, and continued much later (*Dendroacalles* and *Silvacalles*) to the shady and moist laurel forests (= laurisilva). Several lineages of *Laparocerus* have colonised the laurisilva (*Bencomius*, *Guanchotrox*, *Machadotrox*, *Fernandezius*, some groups of *Pecoudius*, etc.) also within the timeframe of the ecological window generated by the trade-winds. It has been confirmed that laurisilva has a Plio-Pleistocene origin, with a few older species from the Upper Miocene (Kondraskov et al. 2015). It is not a relict sample of the Tertiary flora as has been largely thought (Ciferri 1962, Axelrod 1975). *Laparocerus* can be found high in the canopy of trees (*Laurus*, *Persea*, *Ocotea*, *Prunnus*, *Myrica*, etc.), in bushes of all sizes, on the lower plants that grow in the shade or more exposed on the cliffs and openings (*Aeonium*, *Cedronella*, *Geranium*, *Hypericum reflexum*, *Phyllis*, *Ranunculus*, *Rubus*, *Senecio*, etc.). Very few of the twenty laurisilva tree species are left aside by *Laparocerus* (e.g. *Arbutus*, *Visnea*). The conquest of this habitat has been almost complete.

**Pine forest.** *Laparocerus* can be found only occasionally on *Pinus canariensis* (e.g. *Laparocerus combrecitensis*, *Laparocerus tenuepunctatus*), with a remarkable exception: *Laparocerus crassirostris* from Gran Canaria feeds almost exclusively on *Pinus canariensis* (also on *Cistus*, but less) and its flattened body resembles that of the Canary pine weevil *Brachyderes rugatus*, adapted to hide in the cracks of the bark. Both species coexist in the Tamadaba massif and may share the same tree.

The habitat shift to the pine forest has focused on the species growing in the understory (*Chamaecytisus proliferus*, *Lotus* spp., *Cistus monspeliensis*, *Cistus symphytifolius*, *Adenocarpus foliolosus*, etc.) where *Laparocerus* can be really frequent and extraordinarily abundant. There are no records of native pine trees, living or fossil, in the eastern Canary Islands, so it is likely that the habitat shift started early in Gran Canaria.

**Subsurface habitat.** The subterranean environment has been colonised by one specific lineage in Madeira (*Anillobius*) with two endogean single island endemics (not included in the phylogram), and by three lineages in the Canary Islands, which include epigean counterparts. The basal position of *Laparocerus oromii* from La Gomera in *Machadotrox* and of *Laparocerus edaphicus* from Tenerife in *Bencomius* suggest that the dispersal to the underground environment happened early after colonisation of the island. The isolated position of these species would represent the result of a long-lasting anagenetic evolution, or that we are missing other derived subterranean species, either because they have not been yet discovered, or because they went extinct. The concentration of hypogean forms in young islands, La Palma (four spp.) and El Hierro (two spp.), which are mainly large forms adapted to caves and the MSS or mesocavernous shallow substratum (Oromí et al. 1986) suggest that radiation happens in the early stages of island building, when this micro-cave environment is open for life before it gets filled by earth as the island becomes older and erosion prevails. An exception to this rule is *Laparocerus idafe* (not sequenced) from La Palma, which is small and has the typical subcylindrical body of edaphic forms (Villani et al. 1999), that are normally present in mature soil of old islands (Tenerife, La Gomera, Gran Canaria, Madeira and Porto Santo). Subterranean species have been marked with an asterisk in the phylograms, but we have been able to sequence only half of the 14 species known.

Subterranean insect life in volcanic terrains is much richer than originally attributed to oceanic islands. Far from being sterilised, the number of species found in lava tubes and the MSS in the Canary Islands is increasing constantly (Oromí 2010), despite the difficulties to prospect in these confined environments. If the hypothesis of an initial boom and bust speciation phase after island emergence is true, the volcanic mesocavernous shallow substratum (MSS) would be an ideal empty niche for occupation, and *Laparocerus* have shown adaptation capacity for doing so. Indeed, the presence of big, MSS/cave dwelling species only in El Hierro/La Palma and the presence of small, soil-dwelling species in older islands (plus one in La Palma) suggest a substitution of endogean species by hypogean ones as the islands get older. We are possibly facing a richer initial hypogean fauna, and the corollary hypothesis of a greater progressive extinction of *Laparocerus* under the ground rather than above the ground seems plausible, albeit hard to test.

**Figure 15.**
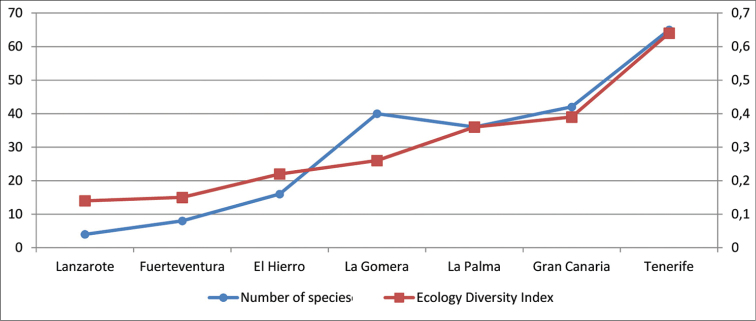
Plot of number of *Laparocerus* species and ecological diversity of each island. Ecological Diversity Index taken from Machado (1998).

**Ecological diversity**. Island size, distance from continent, island age and other factors have been traditionally analysed in shaping oceanic island faunas since the McArthur and Wilson (1967) seminal work on island biogeography. We have found a clear correlation (multiple R = 0.94, R^2^ = 0.88) only between the ecological richness of the island and the number of *Laparocerus* species living in it. The slight deviations observed in Figure 15 are congruent with circumstances previously described, such as the size reduction of the island of La Gomera, or the plausible extinctions of species in Lanzarote and Fuerteventura.

This data suggest that, despite the historical background of colonisation and speciation on each island, it is ecological diversity that controls the extant local fauna.

### Species boundaries

Species are conceptually easy to understand as unitary lineages in evolution, but difficulties arise when it comes to establishing boundaries recognising species in practice. Homologies, cryptic species and budding species are usual nightmares of taxonomists, and *Laparocerus* covers the whole panorama. Molecular techniques have been welcomed to assist in systematic work, especially for unveiling real relationships and by offering quantitative data to fix taxonomic criteria, despite that a common ancestor is not the same for all molecular markers, especially if there was some kind of hybridisation during the segregation process. Species trees and gene trees are rarely identical at leaf levels (Nichols 2001).

It would have been practical to obtain some guiding ranks of genetic p-divergence for delimiting subspecies or species, but the reality in *Laparocerus* is more complex. Divergence may be very high in a species formed by admixture (e.g. 3.9 % in *Laparocerus auarita*); cryptic species may differ strongly in their divergence (e.g. 7.8% in *Laparocerus cryptus*/*Laparocerus morio* or 5.0% in *Laparocerus tibialis*/*Laparocerus tanausu*); conspecific specimens coming from two islands may show divergence reflecting only anagenetic evolution (0.2–1.9%); morphologically distinct species can show almost no divergence (e.g. 0.7% in *Laparocerus depressus*/*Laparocerus gracilis*); or the maximum divergence within species of a given group (e.g. 1.7% in the group of *Laparocerus lepidopterus*) may be less than intraspecific divergence or divergence in sister-species of other groups. There is no common criterion. Genetic divergence can assist taxonomic decisions about species boundaries in *Laparocerus* at most within a given group, and only in the integral context of morphological, biogeographical, and ecological information.

### Why are there so many species of *Laparocerus*?

The species swarm of 236 extant *Laparocerus* in Macaronesia is the result of a blend of adaptive and non-adaptive evolution in old oceanic archipelagos with plenty of environmentally dissected islands with a dynamic and complex volcanic history of construction and deconstruction. Contingencies like sterilising eruptions and mega-landslides shall have played a decisive role in segregation, promoting allopatric and peripheral isolate intra-island speciation, as well as in punctuated dispersal. Oceanic islands are indeed species producing machines (cf. Rosenzweig 1995).

An obligatory question is: why has *Laparocerus* by large the record of island endemics in Macaronesia and not other groups that are almost as old, having evolved in the same scenario and are also flightless? The next in the list are *Napaeus* gastropods with 74 species (updated), *Dysdera* spiders with 72 species (including 16 pending description, Oromí pers. com., Crespo 2013), *Tarphius* beetles with 57 species, *Dolichoiulus* millipedes with 56 species, etc. (Fernández-Palacios 2011).

It has been argued, that some taxa show a greater inherent degree of genetic or morphological plasticity than others, or that they possess traits related to their breeding systems that favour rapid evolutionary change on islands (Whittaker and Fernández-Palacios 2007). Indeed, insects are known to be a successful group in evolution of life, Curculionidae within insects, and Entiminae within Curculionidae (Oberprieler et al. 2007). Entimine weevils are not prone to chemically mediated co-adaptations with new host plants as it occurs in other weevils with endophytic larvae, but they may have some pre-adaptive capacity to colonise isolated oceanic archipelagos, *Laparocerus* being the most striking case (237 species). Parallel but less spectacular radiative speciation is reported for some 130 species of *Rhyncogonus* in the Pacific (Machado 2007a, Gillespie et al. 2008), and nearly 100 species of *Cratopus* (mostly winged!) in the Indian Ocean (Kitson et al. 2013).

If oceanic islands have been traditionally considered as laboratories of evolution, *Laparocerus* will become the ideal guinea-pig for broadening in speciation processes of all kinds, and for studying the role that ‘geological turbulence’ has played in vicariant speciation or massive dispersal. They are flightless, monophyletic, have many endemic species in many islands, and are easy to find. Working with such a group is like getting a picture of nature with more pixels. We hope that several highlighted cases in this discussion (e.g. *Atlantis*, *Aridotrox*, *Fenandezius*, etc.) become stimuli for more intensive sampling and further phylogeographic research in this group. The answer to why there are so many *Laparocerus* is more or less clear; the how is now the challenge.

We are confident that in the near future *Laparocerus* will merit sharing the podium with Darwin´s finches or *Drosophila* in the studies of island evolution.

## Conclusions

Species presently attributed to the genus *Laparocerus* form two monophyletic clades that originated ca. 11.2 Ma ago: the Madeiran clade (TMRCA 8.5 Ma) and the Canarian clade (TMRCA 7.7 Ma). *Laparocerus garretai* from the Selvagens Islands belongs to the Canarian clade. The original continental lineage is presumably extinct, and *Laparocerus susicus* present in the so-called Macaronesian enclave in NW Africa (Morocco), is a back-colonisation from the Canaries, if we accept the hypothesis of an original African source.

The separation of the Madeiran and Canarian clades may have happened in the continent (each archipelago colonized independently) as well as in Madeira (single colonisation), and from there to the Canaries. We keep both clades within the single genus *Laparocerus* in absence of diagnostic features to separate them, and because of similarity in their genetic structuring. A total of 19 monophyletic subclades (six Madeiran, 13 Canarian) has been recognised as subgenera, plus subgenus *Atlantis* from Madeira which shows paraphyly. Successive adaptive and non-adaptive radiation events took place between and within the islands during the Late Miocene and Early Pleistocene, starting in Porto Santo, in the case of the Madeiran Archipelago, and with a general shift from the eastern to the western islands in the Canaries, coincident with the decreasing age of the islands. Fuerteventura, Gran Canaria, Tenerife and La Gomera ‒ or La Gomera and Tenerife ‒ acted sequentially as dispersal platforms, and species radiated profusely within most of the islands. The ancestral ecology of *Laparocerus* remains elusive. Colonisation could have started in some kind of extinct forest or in the semi-arid belt of the islands, and thereafter shifting to the sclerophyllous forest, humid laurisilva, the Canary pine forest and upper mountain vegetation, not necessarily in this order. The genus *Laparocerus*, with 237 species level taxa (36 Madeiran archipelago, two Selvagens, three Morocco, and 196 the Canary Islands), represents an absolute record in species richness in Macaronesia. It is, with the Canarian endemic genus *Moreiba*, the only confirmed representatives of the tribe Laparocerini.

## Author contributions

AM conceived and planned the study, collected and identified the samples, checked the alignments, conducted the analysis, described the new taxa, and wrote the manuscript; MR and ER-E carried out extractions and amplifications of DNA, and MH supervised the molecular analysis, checked results and joined in their interpretation and general discussion.

## Supplementary Material

XML Treatment for Aridotrox

XML Treatment for Purpuranius

XML Treatment for Bencomius

XML Treatment for Belicarius

XML Treatment for Canariotrox

## Appendix 1

**Table:** Specimens used in the phylogenetic analysis.

Taxon	Seq.	Coll code	Specimen origin
MADEIRAN CLADE			
*Laparocerus abditus* Roudier, 1963	4	AMC4143	Madeira: Bco. Joao Gomes,300–450 m
*Laparocerus aenescens* (Wollaston, 1854)	4	AMC4004	Madeira: Ribeiro Frío, 780 m
*Laparocerus angustulus* (Wollaston, 1857)	4	AMC4008	Madeira: Pico do Ariero, 1700 m
*Laparocerus calcatrix* (Wollaston, 1854)	4	AMC3397	Madeira: Montado dos Peçegueiros, 1300 m
*Laparocerus chaoensis cevadae* Roudier, 1961	4	AMC4935	Madeira: Ilheu do Desembarcadouro, 25 m
*Laparocerus chaoensis chaoensis* Uyttenboogaart, 1940	4	AMC5101	Desertas: Ilheu de Châo, 60 m
*Laparocerus chaoensis* ssp.?	[4]	AMC5108	Desertas: Ilheu Bugio, 170 m (leg. I. Silva)
*Laparocerus clavatus* Wollaston, 1854	5	AMC0840	Madeira: Ribeiro Frio, 780 m
*Laparocerus colasi* Roudier, 1958	5	AMC0841	Madeira: Ribeira de Porto Novo, 40 m
*Laparocerus cryptus* Machado, 2008	[4]	AMC0277	Desertas: Deserta Grande, Châo da Doca, 250 m
*Laparocerus cryptus* Machado, 2008	[4]	AMC0252	Madeira: Punta de Sâo Lourenço, 120 m
*Laparocerus cryptus* Machado, 2008	4	AMC305	Porto Santo: Pico do Castelho, 395 m
*Laparocerus distortus* (Wollaston, 1854)	5	AMC0304	Madeira: Encumeada: Folhadal, 100 m (leg. P. Stüben)
*Laparocerus excelsus* (Wollaston, 1854)	5	AMC0292	Madeira: Ponta do Tristâo, 350 m
*Laparocerus fritillus* (Wollaston, 1854)	5	AMC4518	Porto Santo: Pico de Ana Ferreira, 215 m
*Laparocerus hobbit* Machado, 2008	2	AMC5091	Madeira: Faja da Nogueira, 713 m
*Laparocerus inconstans* (Wollaston, 1854)	4	AMC0852	Porto Santo: Calheta, 5 m
*Laparocerus instabilis* (Wollaston, 1854)	5	AMC0846	Porto Santo: Capela da Graça, 133 m
*Laparocerus lamellipes* (Wollaston, 1854)	5	AMC0248	Madeira: Balçoes, 810 m
*Laparocerus lauripotens* (Wollaston, 1854)	[4]	AMC0245	Madeira: Santana, 457 m
*Laparocerus lauripotens* (Wollaston, 1854)	4	AMC0270	Madeira: Curral dos Romeiros, 820 m
*Laparocerus lindbergi* Roudier, 1963	5	AMC4147	Madeira: Paul da Serra: Campo Grande, 1430 m
*Laparocerus madeirensis* Machado, 2008	4	AMC4003	Madeira: Ribeiro Frío, 780 m
*Laparocerus mendax* (Wollaston, 1854	5	AMC3418	Porto Santo: Playa de Ponta da Calheta, 5 m
*Laparocerus morio* Boheman, 1834	5	AMC4013	Madeira: Encumeada, 1030 m
*Laparocerus noctivagans* ssp.?	[4]	AMC5096	Madeira: Calheta, Faja da Ovelha, 400 m
*Laparocerus noctivagans* (Wollaston, 1854)	4	AMC0266	Madeira: Rabaçal, 1060 m
*Laparocerus prainha* Machado, 2008	4	AMC3382	Madeira: Prainha, 30 m
*Laparocerus schaumii* (Wollaston, 1854	4	AMC3413	Porto Santo: Pico do Castelho, 395 m
*Laparocerus serrado* Machado, 2008	4	AMC3406	Madeira: i. Eira do Serrado, 915 m
*Laparocerus silvaticus* Machado, 2008	4	AMC0267	Madeira: Rabaçal, 1060 m
*Laparocerus solifuga* (Fauvel, 1997)	2	AMC5064	Madeira: Sâo Vicente: Grotte, 85 m (leg. P. Süben)
*Laparocerus stuebeni* Machado, 2008	4	AMC5097	Madeira: Calheta: Faja da Ovelha, 400 m
*Laparocerus undulatus* Wollaston, 1862	5	AMC3398	Madeira: Barranco do Inferno, 200 m
*Laparocerus ventrosus* (Wollaston, 1854)	5	AMC3422	Madeira: Achada Grande, 1410 m
*Laparocerus vespertinus* (Wollaston, 1854)	5	AMC0259	Madeira: Pico Ruivo, 1850 m
*Laparocerus waterhousei* (Wollaston, 1854)	5	AMC4020	Madeira: Rabaçal, 1060 m (leg. H. López)
CANARIAN CLADE			
*Laparocerus abona* Machado, 2016	4	AMC3150	Tenerife: Vilaflor: Las Quemadas, km 9,1
*Laparocerus acutipennis* Machado, 2007	4	AMC0225	La Gomera: Bco. de Almagro, 1000 m
*Laparocerus acyphus* Machado, 2009	4	AMC5174	La Palma: El Paso: Mña Don Mendo, 1075 m
*Laparocerus aeneotinctus* Machado, 2009	4	AMC2150	La Palma: Breña Alta, Pared Vieja, 1350 m
*Laparocerus aethiops aethiops* Wollaston, 1864	4	AMC3338	El Hierro: Frontera: Hoya del Pino, 1020 m
*Laparocerus aethiops garajonay* Machado, 2007	4	AMC3371	La Gomera: Apartacaminos, 1030 m
*Laparocerus affinis* Wollaston, 1864	[4]	AMC4533	Gran Canaria: Barranco de los Cernícalos, 1400 m
*Laparocerus affinis* Wollaston, 1864	4	AMC0388	Tenerife: Santa Cruz: Los Campitos, 350 m
*Laparocerus aguiari* Machado, 2007	4	AMC2818	Tenerife: Teno: Las Portelas W, 800 m
*Laparocerus alluaudi alluaudi* Uyttenboogaart, 1940	4	AMC5828	Gran Canaria: Santa Lucía: Mirador Tederas, 818 m
*Laparocerus alluaudi aytamis* Machado, 2012	4	AMC5923	Gran Canaria: Agüimes: Aldea Blanca, 85 m
*Laparocerus alluaudi* spp.?	[4]	AMC6700	Gran Canaria: Presa Niñas: Mña Las Monjas, 958 m
*Laparocerus amicorum* Machado, 2009	4	AMC4066	La Palma: Barranco de los Hombres, 50 m
*Laparocerus amplificatus* (Wollaston, 1865)	4	AMC4754	La Gomera: Enchereda, 725 m
*Laparocerus anagae* Machado, 2015	4	AMC4165	Tenerife: Anaga: Hoya de Ijuana, 600 m
*Laparocerus anniversarius* Machado, 2012	5	AMC2655	Gran Canaria: Barranco de Fataga km 9,2; 460 m
*Laparocerus arcanus* Machado, 2012	4	AMC7077	Gran Canaria: Tufia, 39 m (leg. J. Pelikán)
*Laparocerus arrochai* Machado, 2009	5	AMC3986	La Palma: Franceses: Las Piedras, 440 m
*Laparocerus astralis* Machado, 2009	4	AMC0554	La Palma: Roque de Los Muchachos km 34, 2100 m
*Laparocerus auarita* Machado, 2016	4	AMC2161	La Palma: Garafía: Las Moradas, 2000 m
*Laparocerus auarita* Machado, 2016	[4]	AMC5009	La Palma. Mazo. Venijobre, 830 m
*Laparocerus auctus* (Wollaston, 1864)	5	AMC4787	El Hierro: Tamaduste, 61 m
*Laparocerus bacalladoi* Machado, 2005	5	AMC2580	Tenerife: Valle de San Lorenzo, km 4; 170 m
*Laparocerus bellus* Roudier, 1957	4	AMC2650	Tenerife: Barranco de Tahodio, 600 m
*Laparocerus benchijigua* Machado, 2007	4	AMC4750	La Gomera: Barranco Benchijigua, 675 m
*Laparocerus bentejui bentejui* Machado, 2012	5	AMC0386	Gran Canaria: Barranco de los Cernícalos, 1500 m
*Laparocerus bentejui delicatulus* Machado, 2012	4	AMC2660	Gran Canaria: San Bartolomé: Bco. Tirajana, 900 m
*Laparocerus bentejui robustus* Machado, 2012	4	AMC5942	Gran Canaria: Degollada de Tirma, 676 m
*Laparocerus bimbache* Machado, 2011	4	AMC3360	El Hierro: Tiñor, 1000 m
*Laparocerus bolivari* Uyttenboogaart, 1937	4	AMC3997	Tenerife: Monte del Agua, 900 m
*Laparocerus boticarius* Machado, 2007	4	AMC0886	Tenerife: Los Carrizales, 650 m
*Laparocerus brunneus* Lindberg, 1953	5	AMC0387	Gran Canaria: Barranco de los Cernícalos, 1400 m
*Laparocerus buccatrix* (Wollaston, 1865)	4	AMC4777	La Gomera: Vallehermoso: Piedra Encantada 790 m
*Laparocerus buenavistae* Roudier, 1957	4	AMC3159	Tenerife: Taucho, Barranco de Yé, 900 m
*Laparocerus calvus* Machado, 2011	5	AMC0860	Fuerteventura: Cumbres de Jandía, 600 m
*Laparocerus campestris* Machado, 2015	4	AMC6648	La Palma: Mazo: Lomo Oscuro, 500 m
*Laparocerus canariensis* Boheman, 1842	5	AMC1886	Tenerife: El Portillo, 2000 m
*Laparocerus canescens* Machado, 2016	4	AMC5213	Tenerife: Arico: Contador, 1200 m
*Laparocerus cephalotes* Machado, 2011	5	AMC3347	El Hierro: Frontera: El Lunchón 470 m
*Laparocerus chasnensis* Machado, 2007	5	AMC3140	Tenerife: Ctra. El Frontón - Vilaflor, km 7, 1060 m
*Laparocerus colonnellii* Machado, 2011	4	AMC4996	Fuerteventura: Pájara: Fayagua km 8, 190 m
*Laparocerus combrecitensis* Roudier, 1957	4	AMC0594	La Palma: Barranco del Riachuelo, 1100 m
*Laparocerus combrecitensis* Roudier, 1957	[4]	AMC4063	La Palma: Roque Faro, 1000 m
*Laparocerus compactus* Wollaston, 1864	5	AMC2766	Gran Canaria: San Pedro, Casa del Camino, 200 m
*Laparocerus confusus* Machado, 2011	4	AMC0222	La Gomera: Laguna Grande, 1250 m
*Laparocerus crassifrons* Wollaston, 1863	4	AMC0009	Tenerife: Montaña Blanca, 2500 m
*Laparocerus crassirostris* Wollaston, 1864	5	AMC0366	Gran Canaria: Tamadaba NW, 1200 m
*Laparocerus crassus* Roudier, 1957	4	AMC0893	Tenerife: Anaga: El Pijaral S, 800 m
*Laparocerus cristatus* Machado, 2009	4	AMC0569	La Palma: Infra Jedey, 540 m
*Laparocerus crotchi* Machado, 2016	4	AMC6695	La Gomera: San Sebastián: El Langrero N, 110 m
*Laparocerus curvipes curvipes* Lindberg, 1950	4	AMC5904	Tenerife: San Miguel, 659 m
*Laparocerus curvipes espanoli* Roudier, 1954	5	AMC0861	Fuerteventura: Cumbres de Jandía, 600 m
*Laparocerus curvipes famarae* Machado, 2011	4	AMC0858	Lanzarote: Ermita de las Nieves, 450 m
*Laparocerus dacilae* García, 1988	4	AMC0574	La Palma: Fuencaliente: Cueva Machacadora, 900 m
*Laparocerus debilis* Wollaston, 1865	4	AMC0997	Tenerife: Barranco de Ruiz, 120 m
*Laparocerus decipiens* Machado, 2009	4	AMC2159	La Palma: Garafía: Las Moradas, 2000 m
*Laparocerus depilis* Roudier, 1957	4	AMC4851	Tenerife: Tacoronte; Pista El Rayo, 1427 m
*Laparocerus depressus* Machado, 2007	4	AMC4085	La Gomera: Vegaipala, 870 m
*Laparocerus dilutus* Machado, 2015	4	AMC4784	La Gomera: Barranco Benchijigua, 675 m
*Laparocerus dispar* Wollaston, 1864	4	AMC2170	Lanzarote: Los Valles, 300 m
*Laparocerus dissidens* Machado, 2012	4	AMC5201	Gran Canaria: Arteara, Ctra 48,5 km, 425 m
*Laparocerus dissimilis alticola* Machado, 2016	4	AMC4806	Tenerife: El Portillo, 2000 m
*Laparocerus dissimilis dissimilis* Lindberg., 1950	5	AMC3132	Tenerife: San Miguel 1 km NE, 630 m
*Laparocerus dissimilis infernalis* Machado, 2016	4	AMC3157	Tenerife: Adeje: Barranco del Infierno, 550 m
*Laparocerus edaphicus* Machado, 2008	4	AMC4903	Tenerife: Anaga: Barranco de Ijuana, 780 m
*Laparocerus eliasenae* (Uyttenboogaart, 1929)	5	AMC4164	Gran Canaria: Valsendero: Bco. Cazadores, 1080 m
*Laparocerus ellipticus* Wollaston, 1863	[4]	AMC5949	Gran Canaria: Tiles de Moya, 446 m (leg. P. Stüben)
*Laparocerus ellipticus* Wollaston, 1863	[4]	AMC5181	La Gomera: Los Aceviños, 992 m (leg. P. Stüben)
*Laparocerus ellipticus* Wollaston, 1863	[4]	AMC0033	La Palma: Cubo de La Galga, 600 m
*Laparocerus ellipticus* Wollaston, 1863	4	AMC0177	Tenerife: Anaga, km 5,5 a Chamorga, 800 m
*Laparocerus ellipticus* Wollaston, 1863	[4]	AMC3998	Tenerife: Teno, Monte del Agua, 900 m
*Laparocerus elongatus* Machado, 2009	4	AMC2149	La Palma: Breña Alta, Pared Vieja, 1350 m
*Laparocerus escaleraorum* Uyttenboogaart, 1937	4	AMC0153	Tenerife: Monte del Agua, 900 m
*Laparocerus estevezi* Machado, 2012	4	AMC4580	Gran Canaria: Valsendero, Bco. Cazadores, 1080 m
*Laparocerus excavatus* Wollaston, 1863	5	AMC2135	Tenerife: Anaga: Chinobre, 900 m
*Laparocerus excavatus* Wollaston, 1863	[4]	AMC3999	Tenerife: Teno, Monte del Agua, 900 m
*Laparocerus exiguus* Machado, 2007	5	AMC2179	La Gomera: Laguna Grande, 1250 m
*Laparocerus exophthalmus* Machado, 2007	5	AMC2181	La Gomera: Pajarito, 1360 m
*Laparocerus feloi* Machado, 2009	4	AMC2155	La Palma: Puntagorda: Barranco Herrero, 450 m
*Laparocerus femoralis* Machado, 2009	4	AMC4069	La Palma: Roque Faro, 1080 m
*Laparocerus fernandezi* Roudier, 1957	[4]	AMC5439	La Palma: Todoque: Las Norias 358 m
*Laparocerus fernandezi* Roudier, 1957	4	AMC1885	Tenerife: San Miguel km 49- E, 600 m
*Laparocerus franzi* Machado, 2012	4	AMC4878	Gran Canaria: La Isleta, llano interior, 122 m
*Laparocerus fraudulentus* Machado, 2012	4	AMC6145	Gran Canaria: Agaete: Piso Firme, 110 m
*Laparocerus freyi* Uyttenboogaart, 1940	4	AMC2141	Tenerife: Las Cañadas: El Portillo, 2000 m
*Laparocerus garretai* garretai Uyttenboogaart, 1940	5	AMC0056	Salvajes: Selvagem Grande, 95 m (leg.M. Arechavaleta)
*Laparocerus gerodes* Machado, 2016	4	AMC5903	La Gomera: San Sebastián: La Gerode 630 m
*Laparocerus gomerensis* Lindberg, 1953	4	AMC2178	La Gomera: Laguna Grande, 1250 m
*Laparocerus gracilis* Wollaston, 1864	5	AMC2176	La Gomera: Bco. de la Villa, S 20 m
*Laparocerus grayanus* (Wollaston, 1865)	5	AMC0057	Gran Canaria: Tejeda-Artenara, 1150 m
*Laparocerus grossepunctatus* Wollaston, 1864	5	AMC0109	Tenerife: Anaga, Ctra. Las Carboneras km 1; 700 m
*Laparocerus grossepunctatus* ssp.?	[4]	AMC0190	Tenerife: Agua García, 800 m
*Laparocerus heres* heres Machado, 2007	4	AMC2792	La Gomera: Las Hayas, N. 800 m
*Laparocerus heres* jocoensis Machado, 2007	4	AMC2777	Tenerife: Montaña de Joco, 1958
*Laparocerus hirtus* Wollaston, 1864	4	AMC0356	Gran Canaria: Barranco Oscuro, 900 m
*Laparocerus humeralis* Machado, 2007	4	AMC4776	La Gomera: Hermigua: s. Ermita de San Juan, 650 m
*Laparocerus hupalupa* Machado, 2007	5	AMC0209	La Gomera: Las Hayas, 800 m
*Laparocerus hypogeus* Machado, 2011	4	AMC5435	El Hierro: Frontera: Pista Derrabado, 750 m (leg. GIET)
*Laparocerus hystricoides* Machado, 2012	5	AMC2656	Gran Canaria: San Bartolomé, km 1; 940 m
*Laparocerus impressicollis* (Wollaston, 1864)	4	AMC6809	Tenerife: Anaga: El Pijaral, 800 m (leg. H. López)
*Laparocerus inaequalis globulipennis* Wollaston, 1864	4	AMC0031	La Palma: Cubo de La Galga, 600 m
*Laparocerus inaequalis inaequalis* Wollaston, 1863	5	AMC2138	Tenerife: Anaga: Chinobre, 900 m
*Laparocerus inaequalis inaequalis* Wollaston, 1863	[4]	AMC0158	Tenerife: Teno, Monte del Agua, 900 m
*Laparocerus incomptus* (Wollaston, 1865)	4	AMC0064	El Hierro: Pozo de La Salud, 10 m
*Laparocerus inconspectus* Roudier, 1957	5	AMC0335	Gran Canaria: Brezal del Palmital, 525 m
*Laparocerus indutus* Wollaston, 1865	5	AMC5900	La Gomera: Tamargada: Lomo Palomos, 600 m
*Laparocerus inermis* Machado, 2007	4	AMC4875	La Gomera: Degollada de Peraza, 939 m
*Laparocerus inflatus* Wollaston, 1865	4	AMC4207	La Gomera: El Cedro, 900 m
*Laparocerus juelensis* Machado, 2011	4	AMC6062	La Gomera: Hermigua: Cruz de Juel, 690 m
*Laparocerus junonius* Machado, 2007	4	AMC0221	La Gomera: Chorros de Epina, 800 m
*Laparocerus laevis* Roudier, 1957	[4]	AMC556	La Palma. Pinar de Garafía, 1900 m
*Laparocerus laevis* Roudier, 1957	4	AMC2779	La Palma: Barranco del Riachuelo, 1100 m
*Laparocerus lepidopterus lepidopterus* Wollaston, 1864	[4]	AMC4025	Tenerife: Anaga: Zapata, 980 m
*Laparocerus lepidopterus lepidopterus* Wollaston, 1864	4	AMC0066	El Hierro: Tiñor, 1050 m.
*Laparocerus lepidopterus lepidopterus* Wollaston, 1864	[4]	AMC3369	La Gomera: Apartacaminos, 1030 m
*Laparocerus lepidopterus lepidopterus* Wollaston, 1864	[4]	AMC0036	La Palma: Montaña de Tagoja, 1250 m
*Laparocerus lepidopterus pecoudi* Roudier, 1957	4	AMC0336	Gran Canaria: Brezal del Palmital, 525 m
*Laparocerus longiclava* Lindberg, 1953	4	AMC2680	La Gomera: Degollada de Peraza N, 950 m
*Laparocerus longiclava* Lindberg, 1953	[4]	AMC3158	Tenerife: Taucho: Barranco de Ye, 900 m
*Laparocerus longipennis* Machado, 2011	4	AMC4995	Fuerteventura: Tarajalejo: La Lajita FV 56 km 5.2
*Laparocerus lopezi* Machado, 2008	4	AMC5929	Gran Canaria: Los Tiles de Moya, 524 m
*Laparocerus macilentus* Machado, 2016	4	AMC6852	Tenerife: Adeje: Barranco del Infierno, 490 m
*Laparocerus magnificus* Machado, 2011	4	AMC5901	La Gomera: Tamargada: Lomo Palomos, 600 m
*Laparocerus magnificus* Machado, 2011	[4]	AMC4860	Tenerife: Buenavista N: El Pleito, 200 m
*Laparocerus marmoratus* Machado, 2012	4	AMC2668	Gran Canaria: San Juan: La Montañeta, 320 m
*Laparocerus marmoratus* ssp.?	[4]	AMC2679	Gran Canaria: Agaete - La Aldea km 46.5, 200 m
*Laparocerus mateui mateui* Roudier, 1954	5	AMC3370	La Gomera: Apartacaminos, 1030 m
*Laparocerus mateui tuberosus* Machado, 2011	5	AMC0078	El Hierro: El Gretime, 825 m
*Laparocerus maxorata* Machado, 2011	5	AMC1267	Fuerteventura: Cumbres de Jandía, 600 m
*Laparocerus mendicus* Wollaston, 1864	5	AMC0085	El Hierro: El Fayal, 1350 m
*Laparocerus merigensis* Machado, 2015	4	AMC6652	La Gomera: Caserío de Meriga, 800 m
*Laparocerus microphthalmus* Lindberg, 1950	4	AMC0370	Gran Canaria: Tamadaba NW, 1200 m
*Laparocerus morrisi* Machado, 2009	5	AMC4039	La Palma: Roque de los Muchachos, 2300 m
*Laparocerus mucronatus* Machado, 2009	4	AMC0032	La Palma: Cubo de La Galga, 600 m
*Laparocerus mulagua* Machado, 2007	4	AMC2206	La Gomera: Playa de Hermigua, 10 m
*Laparocerus notatus* Machado, 2015	4	AMC6658	La Gomera: Arguamul: Guillama, 175 m
*Laparocerus obscurus daute* Machado, 2016	4	AMC0867	Tenerife: Teno Bajo, 50 m
*Laparocerus obscurus obscurus* Wollaston, 1864	5	AMC0892	Tenerife: Ctra. a Taganana km 4; 300 m
*Laparocerus obsitus* Wollaston, 1864	4	AMC0382	Gran Canaria: Cuevas Blancas, 1650 m
*Laparocerus obtriangularis* Wollaston, 1864	5	AMC0157	Tenerife: Anaga Km 14, 800 m
*Laparocerus occidentalis* Wollaston, 1864	5	AMC3342	El Hierro: Sendero de Jinamar, km 1.7; 800 m
*Laparocerus oculatissimus* Machado, 2007	4	AMC4742	La Gomera: San Sebastián: Loma del Camello, 350 m
*Laparocerus ornatus* Machado, 2012	4	AMC6032	Gran Canaria: Ayacata km 12 Lomo Aserrador 1440 m
*Laparocerus oromii* Machado, 2008	5	AMC2778	La Gomera: Reventón Oscuro, 1090 m (leg. P. Oromí)
*Laparocerus orone* aridane Machado, 2009	4	AMC0568	La Palma: Barranco de Las Angustias, 200 m
*Laparocerus orone* hierrensis Machado, 2011	4	AMC3351	El Hierro: Valverde –N, 650 m
*Laparocerus orone* orone Machado, 2007	5	AMC2189	La Gomera: Arure, loma del tunel 800 m
*Laparocerus osorio* Machado, 2012	5	AMC1268	Gran Canaria: Valsendero: Bco. Cazadores, 1080 m
*Laparocerus palmensis* Lindberg, 1953	4	AMC4545	La Palma: Los Llanos: Montaña Tenisca, 370 m
*Laparocerus persimilis* (Wollaston, 1864)	4	AMC6789	Tenerife: Icod El Alto, 502 m
*Laparocerus pilosiventris* Machado, 2011	4	AMC5417	La Gomera: Infra Alajeró, 675 m
*Laparocerus propinquus* Lindberg., 1953	4	AMC0367	Gran Canaria: Tamadaba NW, 1200 m
*Laparocerus puncticollis* Wollaston, 1864	5	AMC0067	El Hierro: Tiñor, 1050 m.
*Laparocerus punctiger* Machado, 2016	4	AMC0012	Tenerife: Fuente Joco, 1850 m
*Laparocerus rasus betancor* Machado, 2011	4	AMC0864	Fuerteventura: Morro Velhosa, 640 m
*Laparocerus rasus jandiensis* Machado, 2011	4	AMC0862	Fuerteventura: Cumbres de Jandía, 600 m
*Laparocerus rasus* rasus Wollaston, 1864	5	AMC0859	Lanzarote: Famara, Ermita de las Nieves, 450 m
*Laparocerus rasus* rasus Wollaston, 1864	[4]	AMC2592	Montaña Clara: Caldera 230 m (leg. A.J. Pérez)
*Laparocerus rotundatus* Machado, 2011	4	AMC5416	La Gomera: Infra Alajeró, 675 m
*Laparocerus roudieri* Machado, 2007	4	AMC3364	La Gomera: Vallehermoso: Bco. del Clavo, 365 m
*Laparocerus rugosicollis* Uyttenboogaart, 1937	4	AMC2630	Tenerife: Barranco de Caramujo 1660 m, km 25.1
*Laparocerus rugosivertex* Machado, 2012	4	AMC5170	Gran Canaria: Aldea - Agaete, km 8.5; 200 m
*Laparocerus ruteri* Roudier, 1957	4	AMC2651	Tenerife: Barranco de Tahodio, 575 m
*Laparocerus sanchezi arures* Machado, 2016	4	AMC2191	La Gomera: Cementerio de Arure, 850 m
*Laparocerus sanchezi sanchezi* Roudier, 1957	5	AMC3358	El Hierro: Tiñor, 1000 m
*Laparocerus sanctaecrucis* Machado, 2016	4	AMC4532	Tenerife: Santa Cruz-S: Boca Cangrejo, 132 m
*Laparocerus scapularis* Wollaston, 1864	5	AMC2628	Tenerife: s. Mña. Bermeja, 1600 m
*Laparocerus sculptipennis montivagans* Machado, 2013	4	AMC4680	La Palma: Refugio del Pilar, 1432 m (leg. P. Stüben)
*Laparocerus sculptipennis sculptipennis* (Wollaston, 1864)	5	AMC6447	Tenerife: Cubo de La Galga, 502 m
*Laparocerus sculptus* (Brullé, 1839	4	AMC0527	La Palma: Cubo de La Galga, 500 m
*Laparocerus seminitens* Lindberg, 1950	4	AMC3128	Tenerife: Roque de Jama, NW, 500 m
*Laparocerus semipilosus* Machado, 2012	4	AMC2678	Gran Canaria: Aldea - Agaete km 46.5, 200 m
*Laparocerus separandus* Lindberg, 1953	4	AMC0378	Gran Canaria: Cruz de Tejeda - E, 1500 m
*Laparocerus seriesetosus* (Wollaston, 1864)	4	AMC6889	La Palma: Supra La Caleta, 633 m
*Laparocerus sonchiphagus* Machado, 2015	4	AMC6919	Tenerife: Anaga: Camino a Tafada, 535 m
*Laparocerus soniae* Machado, 2016	4	AMC6698	Gran Canaria: Tenteniguada, 1105 m (leg. S. Abreu)
*Laparocerus spinimanus* Machado, 2007	4	AMC4572	La Gomera: Hermigua: El Tabaibal, 260 m
*Laparocerus squamosus squamosus* (Brullé, 1839	5	AMC0400	Gran Canaria: Tenteniguada W, 875 m
*Laparocerus squamosus* tasarticus Machado, 2012	4	AMC5198	Gran Canaria: Degollada de Tasartico, 560 m
*Laparocerus subcalvus* (Wollaston, 1864)	4	AMC4171	El Hierro: Tiñor, 1000 m
*Laparocerus subnebulosus* (Wollaston, 1864	4	AMC0422	Gran Canaria: El Sâo, 550 m
*Laparocerus subnodosus* (Wollaston, 1864	[3]	AMC6806	Tenerife: Los Rodeos, 638 m
*Laparocerus subnodosus* (Wollaston, 1864	4	AMC6853	Tenerife: Aguamansa, 1089 m
*Laparocerus subopacus* Wollaston, 1865	4	AMC0202	La Gomera: Agulo, 225 m
*Laparocerus subparallelus* Machado, 2007	4	AMC1295	Tenerife: Boca Tauce, 2000 m
*Laparocerus sulcirostris* Wollaston, 1864	4	AMC1272	Gran Canaria: Barranco de la Mina, 1200 m
*Laparocerus supranubius* Machado, 2009	4	AMC2162	La Palma: Garafía: Las Moradas, 2000 m
*Laparocerus susicus inexpectatus* Machado, 2011	4	AMC4105	Marruecos: Tiznit-Aglou, 150 m
*Laparocerus susicus montanus* Machado, 2011	4	AMC2707	Marruecos: Tiznit: Tasgrlt, 550 m
*Laparocerus susicus susicus* (Escalera, 1914)	4	AMC4103	Marruecos: Agadir: La Fortalesa, 60 m
*Laparocerus tafadensis* Machado, 2016	4	AMC6637	Tenerife: Anaga: Camino a Tafada, 584 m
*Laparocerus tanausu* Machado, 2009	5	AMC0051	La Palma: Las Caletas (Fuencaliente), 250 m
*Laparocerus tanausu* Machado, 2009	[4]	AMC1299	Tenerife: Anaga: Roque Fuera, 5 m (leg. M. Arechavaleta)
*Laparocerus tarsalis* Machado, 2009	4	AMC5387	La Palma: Entrada Marcos y Corderos, 1300 m
*Laparocerus tauce* Machado, 2016	4	AMC3135	Tenerife: Vilaflor, Las Quemadas, km 9.1; 1600 m
*Laparocerus teldensis* Machado, 2012	4	AMC4966	Gran Canaria: Telde: Bco de los Cernícalos, 500 m
*Laparocerus tenellus* Wollaston, 1864	4	AMC0749	Tenerife: La Orotava: Rosa de Piedra
*Laparocerus tenicola* Machado, 2015	4	AMC6684	Tenerife: Monte del Agua, 820 m
*Laparocerus tenuepunctatus oppositus* Machado, 2016	4	AMC3147	Tenerife: Vilaflor: La Florida, 1700 m
*Laparocerus tenuepunctatus tenuepunctatus* Roudier, 1957	4	AMC0013	Tenerife: Fuente Joco, 1850 m
*Laparocerus teselinde* Machado, 2015	4	AMC6660	La Gomera: Arguamul: Guillama, 152 m
*Laparocerus tessellatus* (Brullé, 1839)	5	AMC0181	Tenerife: Anaga, pista a Anambro, 810 m
*Laparocerus tesserula* (Wollaston, 1864)	[4]	AMC4167	Tenerife. Bajamar, 20 m
*Laparocerus tesserula* (Wollaston, 1864)	4	AMC6680	Tenerife: Puerto de la Cruz: Martiánez, 15 m.
*Laparocerus tetricus* (Boheman, 1834)	5	AMC0186	Tenerife: Malpaís de Güimar, 25 m
*Laparocerus tibialis* (Wollaston, 1864)	5	AMC0866	Tenerife: Teno Bajo, 50 m
*Laparocerus tinguaro tinguaro* Machado, 2007	5	AMC2140	Tenerife: Anaga: Cabezo de Paybo, 710 m
*Laparocerus tinguaro* tabornoi Machado, 2016	4	AMC0108	Tenerife: Pista Las Yedras, 740 m
*Laparocerus tirajana* Machado, 2012	4	AMC2657	Gran Canaria: San Bartolomé, km 1; 1940 m
*Laparocerus tirmensis* Machado, 2012	4	AMC5941	Gran Canaria: Degollada de Tirma, 676 m
*Laparocerus transversus* Lindberg, 1950	5	AMC4110	Tenerife: Teno, Los Carrizales, 650 m
*Laparocerus undatus* Wollaston, 1864	4	AMC2137	Tenerife: Anaga, Parque Forestal, 800 m
*Laparocerus uyttenboogaarti* Zumpt, 1940	4	AMC2616	Tenerife: Barranco de San Andrés, 200 m
*Laparocerus vestitus* Wollaston, 1864	[4]	AMC0522	La Palma: Montaña Loreal NE, 210 m
*Laparocerus vestitus* Wollaston, 1864	4	AMC5423	Tenerife: Puerto de la Cruz, Bco Las Arenas, 20 m
*Laparocerus vicinus* Lindberg, 1953	5	AMC2658	Gran Canaria: San Bartolomé, km 1: 940 m
*Laparocerus xericola* Machado, 2011	4	AMC0865	Fuerteventura: Rosa Los Negrines, La Oliva, 180 m
*Laparocerus xericola* Machado, 2011	[4]	AMC0856	Lanzarote: Femés, 320 m
*Laparocerus zarazagai subreflexus* Machado & García, 2010	5	AMC2154	La Palma: Bejenado, 1020 m (leg. R. García)
OUTGROUPS			
*Barypeithes indigens* (Boheman, 1834)	5	AMC100147	Madeira: Residencial Encumeada, 1000 m
*Brachyderes rugatus* Wollaston, 1864	5	AMC100228	Gran Canaria: Pinar de Tamadaba, 1200 m

Specimens collected by A. Machado unless otherwise stated. Number of markers sequenced: 5 = mtCOII, 16S rRNA, 12S rRNA, 28S DNA and EF1a; 4 = the same without EF1a, and 2 = only mtCOII and 16S rRNA. Numbers in brackets = sequences not included in the divergence analysis.

## Appendix 2

**Table:** Genetic divergence in Laparocerus weevils.

A. UNCORRECTED P-DISTANCES	COII	12Sr	16S	[28S]	COII+12S+16S	OTUs
Number of nucleotids (gaps deleted)	598	325	422	745	1345	224
Overall mean distance	11.7%	5.4%	5.2%	1.0%	8.2%	224
Within Madeiran clade mean distance	11.1%	7.4%	5.7%	0.8%	8.5%	30
Within Canarian clade mean distance	11.2%	3.9%	4.3%	0.9%	7.3%	194
Between clades mean distance	12.5%	10.2%	8.0%	1.4%	11.0%	224
Net between clade mean distance	2.4%	4.5%	3.0%	0.6%	3.1%	224
**Madeiran clade within group distances**	**COII**	**12Sr**	**16S**	[**28S**]	**COII+12S+16S**	**OTUs**
Atlantis	9.5%	5.7%	4.4%	0.4%	7.0%	10
Atlantodes	6.4%	3.4%	2.8%	0.3%	4.5%	5
Laparocerus	7.9%	4.1%	3.6%	0.4%	5.6%	5
Lichenophagus	n/c	n/c	n/c	n/c	n/c.	1
Pseudatlantis	4.9%	2.2%	2.9%	0.2%	3.6%	5
Wollastonius	4.4%	2.5%	2.4%	0.1%	3.3%	4
**Canarian clade within group distances**	**COII**	**12Sr**	**16S**	[**28S**]	**COII+12S+16S**	**OTUs**
Amyntas	6.6%	1.6%	1.6%	0.6%	3.8%	11
Aridotrox	8.0%	4.1%	2.4%	0.3%	5.3%	9
Belicarius	6.2%	1.0%	1.3%	0.4%	3.4%	17
Bencomius	6.8%	1.9%	2.0%	0.5%	4.1%	14
Canariotrox	6.8%	1.4%	1.8%	0.6%	3.9%	15
Faycanius	6.4%	1.1%	1.1%	0.3%	3.5%	7
Fernandezius	4.5%	1.4%	1.0%	0.3%	2.9%	13
Fortunotrox	8.3%	2.3%	3.8%	0.4%	5.4%	15
Guanchotrox	8.8%	1.8%	2.2%	0.6%	5.1%	30
[*Incertae sedis* (* not averaged)	10.7%	3.9%	4.0%	0.8%	7.0%	6]
Machadotrox	7.9%	2.1%	2.6%	0.4%	4.8%	12
Mateuius	7.7%	2.6%	3.0%	0.7%	5.0%	7
Pecoudius	8.4%	2.1%	2.5%	0.4%	5.1%	32
Purpuranius	7.7%	5.5%	3.0%	0.3%	5.7%	6
**Within group mean distance by clades***	**COII**	**12Sr**	**16S**	[**28S**]	**COII+12S+16S**	**OTUs**
Average within group mean distance	7.1%	2.6%	2.5%	0.4%	4.6%	224
Average in Madeiran Clade	6.6%	3.6%	3.2%	0.3%	4.8%	30
Average in Canarian Clade	7.2%	2.2%	2.2%	0.5%	4.5%	194
**Between group mean distance by clades**	**COII**	**12Sr**	**16S**	[**28S**]	**COII+12S+16S**	**OTUs**
Average between group mean distance	12.4%	7.3%	6.2%	1.1%	9.2%	224
Average in Madeiran Clade	12.0%	8.3%	6.6%	0.9%	9.4%	30
Average in Canarian Clade	11.5%	4.5%	4.6%	0.9%	7.6%	195
**B. CORRECTED DISTANCE**					**COII+12S+16S**	**OTUs**
Overall mean corrected distance					10.5%	224
Average between group mean distance					12.2%	224
Average group age					3.98	224
Divergence rate Ma^-1^					**3.1**%	224
Substitution rate Ma^-1^					1.53%	224

## Appendix 3

**Table:** Plant genera and their families (f. Mabberley 1997).

Genus	Family	Genus	Family	Genus	Family
*Adenocarpus*	Leguminosae	*Erica*	Ericaceae	*Persea*	Lauraceae
*Aeonium*	Crassulaceae	*Euphorbia*	Euphorbiaceae	*Phoenix*	Palmae
*Arbutus*	Ericaceae	*Geranium*	Geraniaceae	*Phyllis*	Rubiaceae
*Argyranthemum*	Compositae	*Jasminum*	Oleaceae	*Pinus*	Pinaceae
*Artemisia*	Compositae	*Juniperus*	Cupressaceae	*Pistacia*	Anacardiaceae
*Asteriscus*	Compositae	*Kleinia*	Compositae	*Prunus*	Rosaceae
*Bituminaria*	Leguminosae	*Launaea*	Compositae	*Ranunculus*	Ranunculaceae
*Bosea*	Amaranthaceae	*Laurus*	Lauraceae	*Rhamnus*	Rhamnaceae
*Bupleurum*	Umbelliferae	*Lavatera*	Malvaceae	*Rubia*	Rubiaceae
*Carlina*	Compositae	*Limonium*	Plumbaginaceae	*Rumex*	Polygonaceae
*Cedronella*	Labiatae	*Lotus*	Leguminosae	*Ruta*	Rutaceae
*Chamaecytisus*	Leguminosae	*Marcetella*	Rosaceae	*Salsola*	Chenopodiaceae
*Cistus*	Cistaceae	*Maytenus*	Celastraceae	*Salvia*	Labiatae
*Crambe*	Cruciferae	*Myrica*	Myricaceae	*Senecio*	Compositae
*Cytisus*	Leguminosae	*Ocotea*	Lauraceae	*Sideritis*	Labiatae
*Dracaena*	Dracaenaceae	*Olea*	Oleaceae	*Sonchus*	Compositae
*Echium*	Boraginaceae	*Periploca*	Asclepiadaceae	*Visnea*	Theaceae

## Supplementary materials

Supplementary material 1 Mitochondrial 3-gene phylogram of genus *Laparocerus* Schönherr, 1834 from Macaronesia (Coleoptera, Curculionidae, Entiminae) Data type: Portable document file Explanation note: Bayesian 50% majority rule consensus tree for COII, 16SrNA, and 12S rRNA: posterior probabilities above the branches. The analysis involved 256 nucleotide sequences. All positions containing gaps and missing data were eliminated except in the 12S rRNA sequence (gapcoded). There was a total of 1389 positions in the final dataset total OTUs = 256. Scale, genetic divergence. This dataset is made available under the Open Database License (http://opendatacommons.org/licenses/odbl/1.0/). The Open Database License (ODbL) is a license agreement intended to allow users to freely share, modify, and use this Dataset while maintaining this same freedom for others, provided that the original source and author(s) are credited.A. Machado, E. Rodríguez-Expósito, M. López, M. Hernández

Supplementary material 2 4-gene phylogram of genus *Laparocerus* Schönherr, 1834 from Macaronesia (Coleoptera, Curculionidae, Entiminae) Data type: Portable document file Explanation note: Bayesian 50% majority rule consensus tree for COII, 16SrNA, 12S rRNA, and 28S rRNA: posterior probabilities above the branches. The analysis involved 245 nucleotide sequences. All positions containing gaps and missing data were eliminated except in the 12S rRNA sequence (gapcoded). There was a total of 2153 positions in the final dataset. Scale bar = genetic divergence. This dataset is made available under the Open Database License (http://opendatacommons.org/licenses/odbl/1.0/). The Open Database License (ODbL) is a license agreement intended to allow users to freely share, modify, and use this Dataset while maintaining this same freedom for others, provided that the original source and author(s) are credited.A. Machado, E. Rodríguez-Expósito, M. López, M. Hernández

Supplementary material 3 Chronogram of genus *Laparocerus* Schönherr, 1834 from Macaronesia (Coleoptera, Curculionidae, Entiminae) Data type: Portable document file Explanation note: Timetree generated with MEGA7 (Kumar et al. 2016) using the RelTime method. Divergence times for all branching points in the Bayesian phylogram (COII, 16S rRNA, 12S rRNA) calculated using Maximum Likelihood method with the General Time Reversible model. Relative times were optimized and converted to absolute divergence times (shown next to branching points) based on not allowing species cluster marked with ♦ to be older than the age of the island of La Palma (1,72 Ma) or younger than 0.21 Ma. Bars around each node represent 95% confidence intervals which were computed using the method described in Tamura et al. (2013). The estimated log likelihood value is -32807.3327. A discrete gamma distribution was used to model evolutionary rate differences among sites (6 categories (+G, parameter = 0.5622)). The rate variation model allowed for some sites to be evolutionarily invariable ([+I], 42.7318% sites). The analysis involved 255 nucleotide sequences. All positions containing gaps and missing data were eliminated. There were a total of 1357 positions in the final dataset. Taxon codes include an abbreviation of species name, its code number, and the initial of the island of origin (see Appendix 1). This dataset is made available under the Open Database License (http://opendatacommons.org/licenses/odbl/1.0/). The Open Database License (ODbL) is a license agreement intended to allow users to freely share, modify, and use this Dataset while maintaining this same freedom for others, provided that the original source and author(s) are credited.A. Machado, E. Rodríguez-Expósito, M. López, M. Hernández
