# Supplementary figures and images for: Phylogenetic analysis of the genus Laparocerus, with comments on colonisation and diversification in Macaronesia (Coleoptera, Curculionidae, Entiminae)
Source: Zookeys. 2017 Feb 2;(651):1–77. doi: 10.3897/zookeys.651.10097 (PMC5345357; doi:10.3897/zookeys.651.10097)

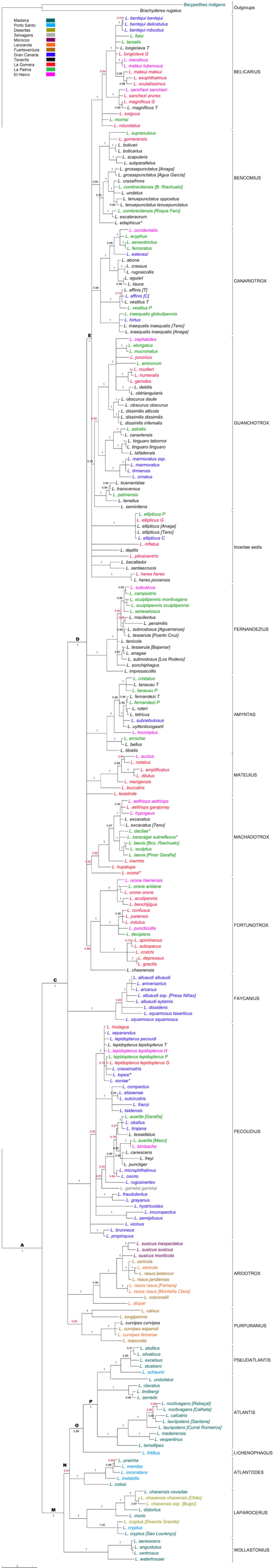

Supplement: Supplementary material 1 — Mitochondrial 3-gene phylogram of genus Laparocerus Schönherr, 1834 from Macaronesia (Coleoptera, Curculionidae, Entiminae) [file zookeys-651-001-s001.pdf]

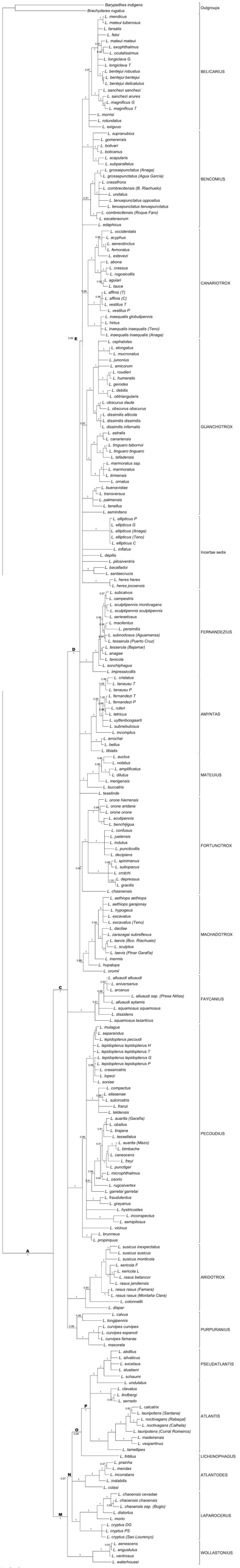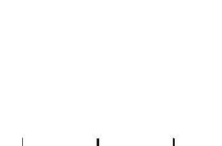

Supplement: Supplementary material 2 — 4-gene phylogram of genus Laparocerus Schönherr, 1834 from Macaronesia (Coleoptera, Curculionidae, Entiminae) [file zookeys-651-001-s002.pdf]
