# Supplementary material for: Phylogenetic analysis of the genus Laparocerus, with comments on colonisation and diversification in Macaronesia (Coleoptera, Curculionidae, Entiminae)
Source: Zookeys. 2017 Feb 2;(651):1–77. doi: 10.3897/zookeys.651.10097 (PMC5345357; doi:10.3897/zookeys.651.10097)

Chronogram of genus *Laparocerus* Schönherr, 1834 from Macaronesia (Coleoptera, Curculionidae, Entiminae)

A. Machado, E. Rodríguez, M. López, M. Hernández

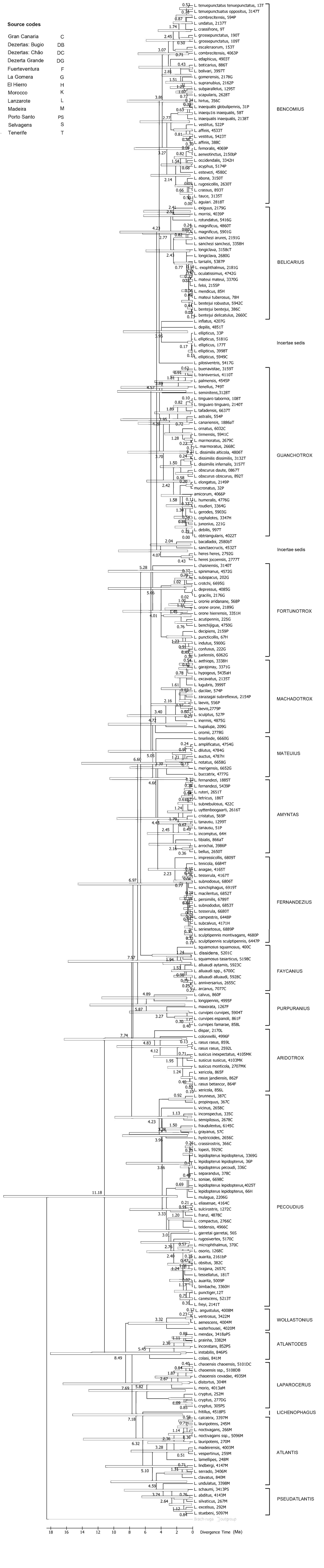

Supplement: Supplementary material 3 — Chronogram of genus Laparocerus Schönherr, 1834 from Macaronesia (Coleoptera, Curculionidae, Entiminae) [file zookeys-651-001-s003.pdf]
